# Thymol-based deep eutectic solvents: a comprehensive overview of classifications, preparation methods, stability, and thermophysical properties

**DOI:** 10.1039/d6ra05714d

**Published:** 2026-10-22

**Authors:** Rahmat Sadeghi, Nosaibah Ebrahimi

**Affiliations:** a Department of Chemistry, University of Kurdistan Sanandaj Iran rahsadeghi@yahoo.com rsadeghi@uok.ac.ir +988733624133 +988733624133

## Abstract

Thymol, a naturally occurring phenolic monoterpenoid predominantly found in *Thymus kotschyanus* and *Achillea ageratum*, has emerged as a key building block in the design of hydrophobic deep eutectic solvents (DESs). Owing to its strong ability to form stable eutectic systems with a wide variety of hydrogen bond donors and acceptors, including carboxylic acids, amides, amines and nitrogen-containing heterocycles, alcohols and diols, terpenoids (oxygenated derivatives of terpenes), quaternary ammonium salts, phenols and their derivatives, as well as other functional compounds, thymol has enabled the development of both binary and ternary DES formulations. These thymol-based DESs are increasingly recognized as a versatile class of green solvents, offering key advantages such as low toxicity, biodegradability, tunable physicochemical properties, and simple, energy-efficient preparation. This review provides a comprehensive and systematic overview of thymol-based DESs, with particular emphasis on their classification, preparation strategies, and phase behavior. Key thermophysical properties, including melting point, viscosity, density, and thermal stability, are critically analyzed, alongside the stability of these systems in aqueous environments to better understand their practical applicability. In addition, the broad application landscape of thymol-based DESs is briefly investigated, spanning extraction technologies for analytical, separation, and decontamination purposes; CO_2_ capture systems with tunable absorption performance; and bioactive roles, including antibacterial and antifungal activity. Emerging applications, such as hydrophobic eutectogels, biomass processing, transdermal drug delivery, green synthesis of functional nanomaterials, and bioactive plasticizers for sustainable food packaging, are also discussed to underscore their growing interdisciplinary relevance. By consolidating current knowledge and identifying existing research gaps, this review aims to provide valuable insights into the design, properties, and future potential of thymol-based DESs as multifunctional and sustainable solvents for advanced scientific and industrial applications.

## Abstract 2

Thymol, a naturally occurring phenolic monoterpenoid predominantly found in *Thymus kotschyanus* and *Achillea ageratum*, has emerged as a key building block in the design of hydrophobic deep eutectic solvents (DESs).

## Graphical abstract

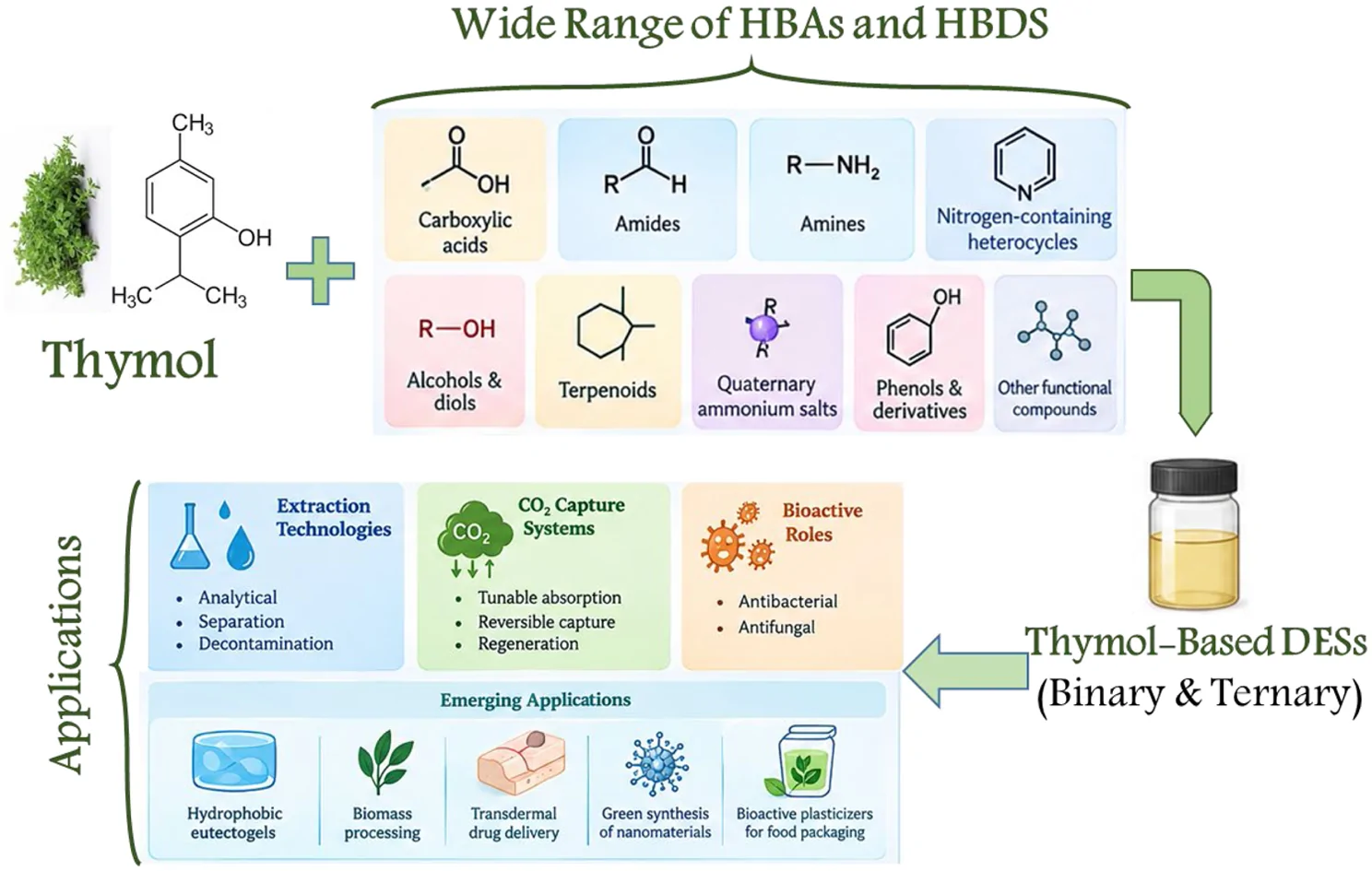

## 1. Introduction

The increasing demand for sustainable chemical processes has accelerated the development of environmentally benign solvent systems capable of replacing conventional volatile organic solvents. Among the emerging alternatives, ionic liquids (ILs), bio-based solvents, supercritical fluids, and deep eutectic solvents (DESs) have received considerable attention due to their tunable physicochemical properties and potential for greener processing. However, each solvent class presents specific limitations. Water, despite being abundant and non-toxic, is unsuitable for many organic transformations and extraction processes due to its high polarity and limited solvation capability for hydrophobic compounds. Supercritical carbon dioxide provides a clean extraction medium but requires high-pressure equipment, increasing operational complexity and cost. Although ILs exhibit remarkable thermal stability and structural tunability, their practical implementation is often restricted by high synthesis costs, purification requirements, persistence concerns, and potential toxicity associated with certain ionic structures.^1–4^ These limitations have stimulated interest in DESs as a simpler and more sustainable solvent platform.

DESs are liquid mixtures formed by combining two or more components, commonly a hydrogen bond acceptor (HBA) and a hydrogen bond donor (HBD), that exhibit a significant melting point depression compared with their individual constituents. This phenomenon originates from strong intermolecular interactions, particularly hydrogen bonding, charge delocalization, and van der Waals interactions, which disrupt the crystal lattices of the original compounds. Since the first report by Abbott *et al.*^5^ describing the choline chloride (ChCl)/urea system with a melting point of 12 °C compared with those of pure ChCl (302 °C) and urea (133 °C), DESs have rapidly evolved into a versatile family of designer solvents. Their low vapor pressure, simple preparation, compositional flexibility, and generally favorable environmental profile have promoted their application in extraction, catalysis, electrochemistry, biomass processing, pharmaceuticals, and separation technologies.^6–8^

Despite these advantages, DESs should not be considered universally sustainable solvents. Their environmental performance strongly depends on the chemical nature of their components, preparation route, viscosity, recyclability, and long-term stability. For example, some DESs exhibit high viscosity, which limits mass transfer and process efficiency, while others show insufficient water stability or incomplete biodegradation despite being prepared from bio-derived constituents. Therefore, rational molecular design and comprehensive characterization of DES properties are essential for evaluating their practical applicability rather than relying solely on their “green” designation. More broadly, although ILs and DESs have often been promoted as green solvent alternatives, their environmental benefits are not universal and have increasingly been questioned in the literature because of factors such as synthesis and purification costs, energy requirements, component degradation during use, toxicity, persistence, and challenges associated with recovery and recycling.

Since their introduction, DESs have been classified according to their chemical composition and interaction mechanisms. The classical classification includes Type I systems (quaternary ammonium salts and metal chlorides),^9^ Type II systems (quaternary ammonium salts and hydrated metal chlorides),^10^ Type III systems (quaternary ammonium salts with molecular hydrogen bond donors such as carboxylic acids, amides, and alcohols),^5^ and Type IV systems (metal chlorides combined with hydrogen bond donors).^11^ More recently, Type V DESs have emerged as an important class consisting exclusively of non-ionic molecular components, such as terpenes, fatty acids, phenolic compounds, and other hydrogen-bonding molecules.^7^ Unlike conventional DESs containing ionic species, Type V systems provide new opportunities for designing hydrophobic solvents with reduced ionic character and improved compatibility with nonpolar compounds. In parallel, specialized DES families, including natural deep eutectic solvents (NADESs), therapeutic deep eutectic solvents (THEDESs), polymeric DESs, and hydrophobic DESs, have expanded the application scope of these materials.^12,13^

Thymol has emerged as a promising bio-based component for hydrophobic DESs due to its phenolic structure, hydrogen-bonding ability, hydrophobicity, and intrinsic bioactivity. It forms stable eutectic systems with various compounds, enabling tunable polarity and selective interactions for applications in extraction, separation, gas absorption, and bioactive systems. However, current research on thymol-based DESs remains fragmented, with limited understanding of the relationships between molecular structure, thermophysical properties, stability, and practical performance. Moreover, their sustainability requires critical evaluation considering toxicity, biodegradability, and scalability. This review therefore systematically examines the classification, preparation, phase behavior, stability, and thermophysical properties of thymol-based DESs, while discussing their applications, limitations, and future prospects as multifunctional green solvents.

## 2. Preparation methods of DESs

Various methods have been developed for the synthesis of DESs, each designed to optimize different aspects of the process, including efficiency, scalability, and the physical properties of the resulting solvents.^14^Fig. 1 provides a graphical overview of all these synthesis methods.

**Fig. 1:**
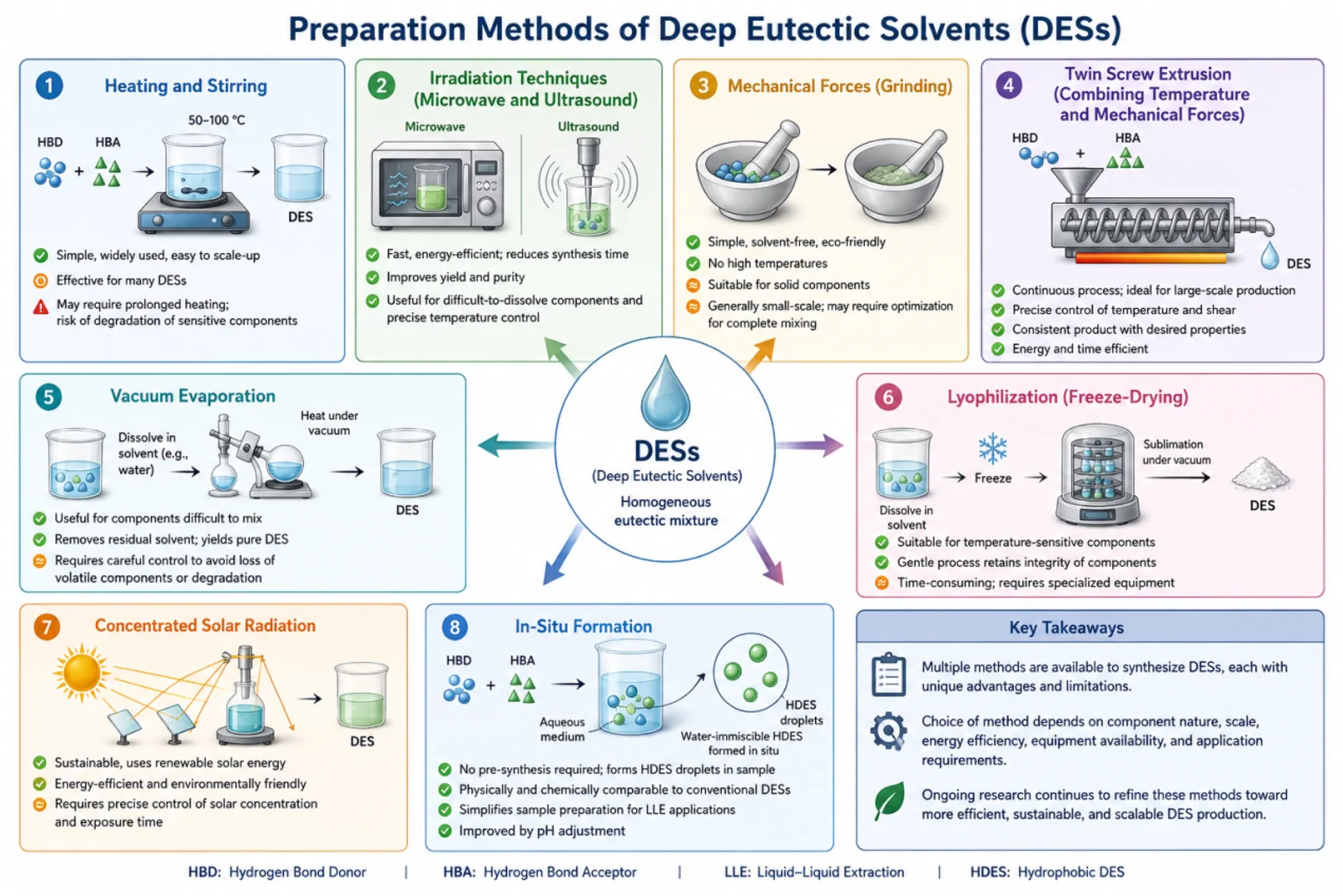
The methods developed for the synthesis of DESs.

### 2.1 Heating and stirring

One of the most straightforward and widely used methods for synthesizing DESs involves the simple combination of the components under controlled heating and stirring. In this method, HBD and HBA are mixed and heated at temperatures typically ranging from 50 to 100 °C while being stirred until a homogeneous liquid is formed. This approach is effective for many DESs and is favored for its simplicity and ease of scale-up. However, it may require prolonged heating for certain mixtures, potentially leading to degradation of sensitive components.^15^ For example, Abbott *et al.*^15^ prepared several ChCl : carboxylic acid DESs by stirring ChCl with the corresponding carboxylic acid at 100 °C until a homogeneous, colorless liquid was obtained, followed by cooling at a rate of 1 °C min^−1^.

### 2.2 Irradiation techniques (microwave and ultrasound)

Irradiation methods, such as microwave^16^ and ultrasound,^17^ offer a faster and more energy-efficient alternative to conventional heating. Microwave irradiation involves exposing the mixture of DES components to microwave energy, which rapidly heats the mixture due to the dielectric heating effect. This method significantly reduces the synthesis time and can improve the yield and purity of the final product. Ultrasound irradiation, on the other hand, uses high-frequency sound waves to induce cavitation in the liquid mixture, which enhances the molecular interactions between the components. Both microwave and ultrasound methods are particularly useful for synthesizing DESs with components that are difficult to dissolve or that require precise temperature control. For example, Yap *et al.*^18^ prepared a ChCl : malic acid DES using microwave irradiation, achieving a preparation time of approximately 5 min and a 92.8% reduction in energy consumption compared with conventional heating and stirring. Similarly, Bajkacz and Adamek^17^ prepared several NADESs based on ChCl, acetylcholine chloride, betaine, as HBA and several carboxylic acids as HBD using an ultrasound-assisted method, in which the components and a calculated amount of deionized water were sealed in a glass vial and sonicated at 37 kHz and 30 W at 50 °C for 10–15 min until a homogeneous liquid was formed.

### 2.3 Mechanical forces (grinding)

Mechanical grinding is another method for DES synthesis, particularly useful for solid components.^19^ In this process, the components are ground together using a mortar and pestle or a ball mill. The mechanical force applied during grinding facilitates the intimate mixing of the components and promotes the formation of a eutectic mixture. This method is advantageous for its simplicity and for avoiding the need for solvents or high temperatures, making it an eco-friendly and energy-efficient approach. However, it is generally more suitable for small-scale synthesis and may require optimization to ensure complete mixing. For example, Florindo *et al.*^19^ prepared ChCl : glutaric acid DES by grinding the components together at room temperature in a mortar until a homogeneous liquid phase was formed, highlighting the effectiveness of this solvent-free method, particularly for pharmaceutical applications.

### 2.4 Twin screw extrusion (combining temperature and mechanical forces)

Twin screw extrusion is an advanced method that combines both temperature and mechanical forces to synthesize DESs continuously.^20^ In this process, the components are fed into an extruder, where they are subjected to mechanical shear forces and controlled heating as they are mixed and conveyed along the screws. This method is particularly effective for large-scale production of DESs and offers precise control over the synthesis conditions, allowing for the consistent production of DESs with desired properties. Twin screw extrusion is also highly efficient in terms of energy consumption and time, making it a promising technique for industrial applications. A representative example of the twin screw extrusion method was reported for the continuous-flow synthesis of ChCl : urea (1 : 2), where the DES components were subjected to mechanochemical mixing and reaction within a twin screw extruder, producing a high-purity DES with substantially enhanced space-time yields compared with conventional batch preparation methods.^21^

### 2.5 Vacuum evaporation

The vacuum evaporation method involves dissolving the initial DES components in a suitable solvent, typically water, to form a homogeneous solution. The solution is then heated under reduced pressure (vacuum) to evaporate the solvent, leaving behind the DES.^22^ This method is particularly useful when dealing with components that are difficult to mix directly or require a solvent to facilitate the formation of the eutectic mixture. However, its applicability is limited by the solubility of the DES components in the selected solvent; poorly soluble components may not form a sufficiently homogeneous solution, thereby restricting the range of DES compositions that can be prepared by this approach. In addition, careful control of the evaporation conditions is required to ensure complete solvent removal while minimizing the loss of volatile components or thermal degradation of the DES. A representative example of the vacuum evaporation method was reported by Dai *et al.*,^22^ who synthesized various DESs, including ChCl-based organic acid, polyol, sugar, sugar alcohol, and amino acid systems (*e.g.*, ChCl : lactic acid, ChCl : glycerol, and ChCl : proline : malic acid), by dissolving the components in water followed by solvent removal at 50 °C using a rotary evaporator.

### 2.6 Lyophilization (freeze-drying)

Lyophilization, or freeze-drying, is another method used to synthesize DESs, especially when dealing with temperature-sensitive components.^23^ In this process, the components are first dissolved in a solvent and then frozen. The frozen mixture is subjected to sublimation under vacuum, where the solvent is removed as a gas, leaving behind the DES in a solid or semi-solid state. Lyophilization is particularly useful for preparing DESs that require gentle processing conditions to maintain the integrity of the components. However, this method can be time-consuming and may require specialized equipment. A representative example of the lyophilization (freeze-drying) method was reported for the preparation of ChCl-based DESs, including ChCl : urea and ChCl : thiourea systems. In this approach, aqueous solutions of urea (or thiourea) and ChCl at a 2 : 1 molar ratio were mixed, frozen at different temperatures (77 or 253 K), and subsequently freeze-dried to remove water, yielding clear viscous DES liquids denoted.^23^

### 2.7 Concentrated solar radiation

The use of concentrated solar radiation is an innovative and sustainable approach to DES synthesis.^24^ In this method, solar energy is concentrated using mirrors or lenses to generate high temperatures that drive the formation of the eutectic mixture. This method is highly energy-efficient and environmentally friendly, as it utilizes a renewable energy source. Concentrated solar radiation is particularly suitable for regions with high solar insolation and can be applied to both small-scale and large-scale synthesis. However, it requires precise control of the solar concentration and exposure time to achieve the desired synthesis conditions. Dazat *et al.*^25^ introduced a solar-assisted method for the preparation of DESs using a 3D-printed parabolic solar concentrator, enabling the synthesis of different DES types, including lactic acid : glucose : water, citric acid : glucose : water, and glycerol : ChCl : water systems, by direct solar heating of the mixed components to form homogeneous deep eutectic liquids.

### 2.8 *In Situ* formation

In addition to the aforementioned methods, another approach, known as the *in situ* preparation method, is also used for preparing the hydrophobic DESs (HDESs). The *in situ* formation of HDESs is a method where the individual components of the HDESs, which might be liquid or solid at room temperature, are directly added to an aqueous medium to form water-immiscible liquid droplet within the sample itself, rather than pre-synthesizing the HDES separately. Studies comparing the IR spectra of HDESs formed *in situ* in water with those of neat HDESs (prepared by heating and stirring) showed very similar features, indicating that the HDESs formed *in situ* are chemically comparable to those prepared conventionally. Similarly, there was no significant difference in the density of HDESs prepared by heating without water and those formed *in situ* in water, suggesting consistent physical properties.^26^ The *in situ* HDES formation is particularly valuable in liquid–liquid microextraction as it allows for the direct application of solid DES components into samples, simplifying the overall analytical procedure. An improvement in the *in situ* HDES formation has been achieved through pH Adjustment. Li *et al.*^26^ prepared HDESs directly in aqueous solutions from thymol, menthol, or camphor combined with fatty acids (C7–C10) or salol, yielding 17 different HDESs upon mild heating above 30 °C, with thymol : heptanoic acid (2 : 1) identified as the most effective system for extraction of fluoroquinolone antibiotics.

The various methods for synthesizing DESs offer a range of options depending on the specific requirements of the application. Heating and stirring, irradiation, mechanical grinding, twin screw extrusion, vacuum evaporation, lyophilization, and concentrated solar radiation each have their own advantages and limitations. The choice of method depends on factors such as the nature of the components, the desired scale of production, energy efficiency, and the need for specialized equipment. As research in this field continues, these methods are likely to be further refined, leading to even more efficient and sustainable processes for DES synthesis.

## 3. Solid–liquid equilibria phase diagram

The solid–liquid equilibria (SLE) phase diagram of DESs serves as a unique identifier, or “fingerprint,” for each specific DES, playing a crucial role in understanding the melting behavior and stability of these mixtures. Mastery of the SLE phase diagram is vital for optimizing both the synthesis and application of DESs, as it provides valuable insights into their thermal properties, stability, and suitability for temperature-sensitive applications. Typically, a phase diagram for DESs maps the relationship between temperature and composition, showing how the melting point of a mixture changes with varying component ratios (Fig. 2).

**Fig. 2:**
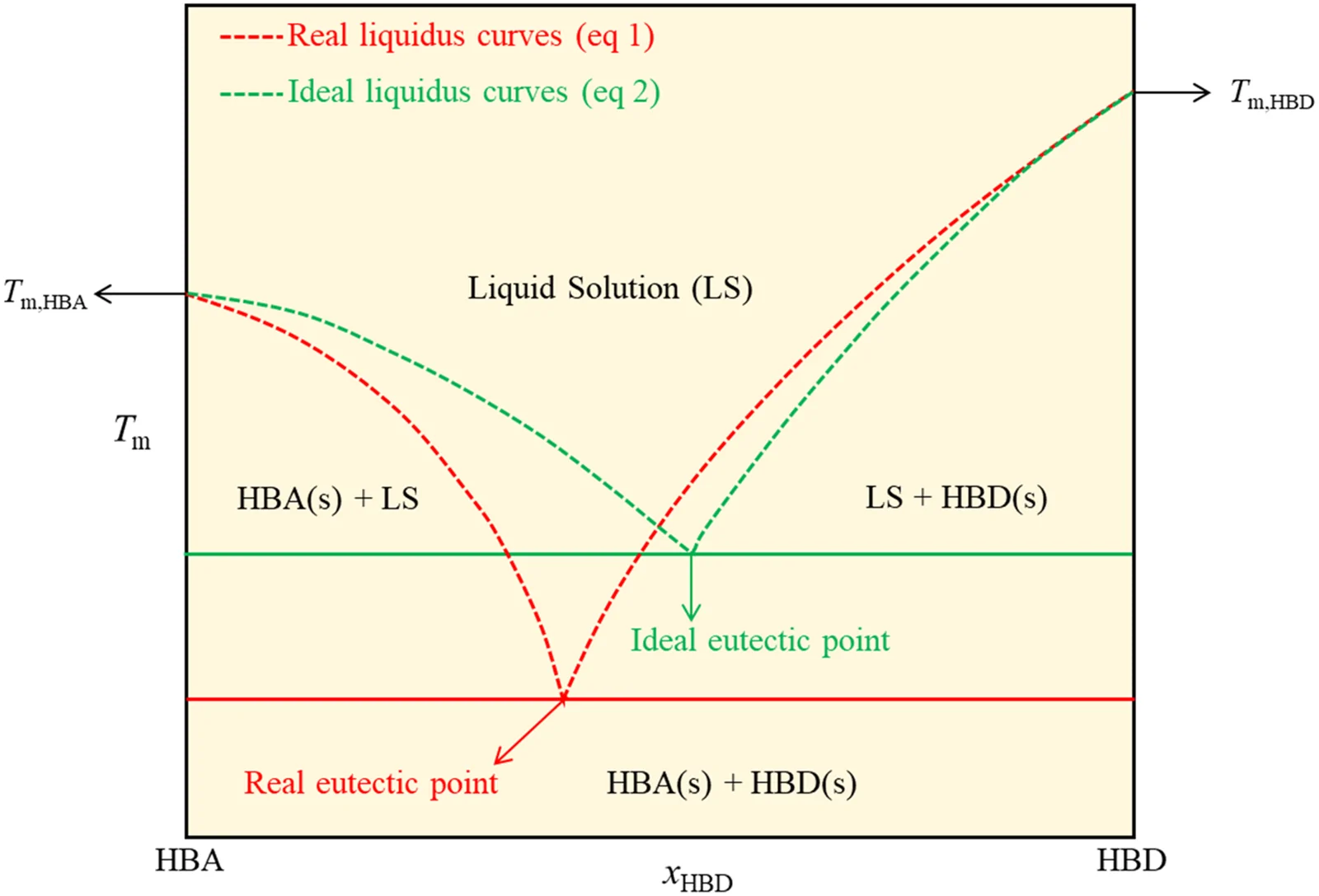
Solid–liquid equilibria phase diagram of typical ideal eutectic mixture (green) and a deep eutectic solvent (red).

When two compounds, HBA and HBD, are combined, their individual pure melting temperatures (*T*_m,HBA_ and *T*_m,HBD_) decrease until reaching a minimum at the eutectic temperature. The eutectic point marks the composition and temperature where the melting curves of the two components intersect. Assuming immiscibility between HBA and HBD in the solid phase (*x*^s^_*i*_*γi*^s^ = 1) and the absence of any solid–solid transitions, the melting curves can be described by the following thermodynamic equation:^27^.1
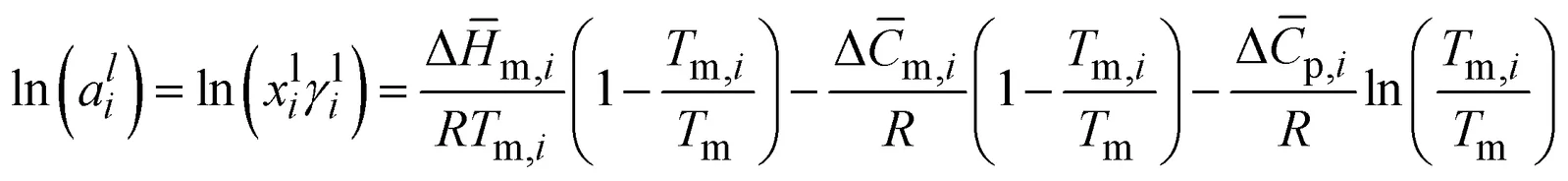
here*T*_m_,_*i*_,Δ*H̄*_m_,_*i*_ and Δ*C̄*_p,*i*_ represent the melting point, mole fraction, molar melting enthalpy, and molar melting heat capacity of component *i* (HBD or HBA), respectively, while *R* is the universal gas constant. The terms *γ*_*i*_ and *a*_*i*_ denote the activity coefficient and activity of component *i* in the liquid phase (*l*), and *T*_m_ is the melting point of a solution with mole fraction *x*_*i*_.

Each component (HBD or HBA) has its own liquidus curve, which intersects at the eutectic point where the mixture exhibits the lowest possible melting temperature, leading to co-crystallization in a specific proportion. This is often characterized by a sharp decline in the melting point as the components approach the eutectic ratio, which is a key indicator of DES formation. The significant reduction in melting point is due to favorable interactions between the HBA and HBD. While lowering the melting point relative to the pure components is necessary for eutectic formation, it is not sufficient on its own. For a DES to form, the mixture must show a substantial negative deviation from ideal behavior, resulting in a large decrease in the freezing temperature compared to the individual components.^28–31^ This deviation indicates that the HBA–HBD interactions are significantly stronger than those between similar molecules. A DES is thus defined as a mixture where the eutectic point temperature is lower than that of an ideal liquid mixture, demonstrating significant negative deviations from ideality. The temperature depression, which is the difference between the ideal and real eutectic points, distinguishes a deep eutectic mixture from a simple eutectic one.^31,32^

In an ideal solution, interactions between the components are uniform across all molecules, regardless of species. Neglecting the slight difference between the heat capacities of the liquid and solid phases (Δ*C̄*_p,i_), eqn (1) reduces to the following form for the melting temperature of an ideal mixture, *T*_m,ideal_:2
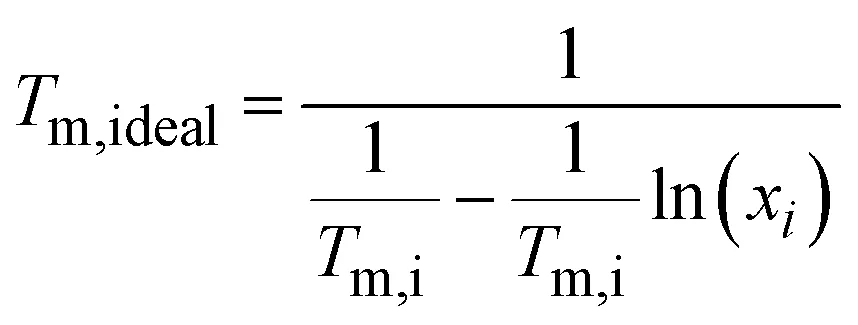


In Fig. 2, the liquidus curves for ideal behavior are also depicted. Beyond the eutectic composition, the phase diagram typically shows regions where one component remains in excess and begins to solidify separately, leading to distinct phase behaviors. Understanding this phase behavior is essential for correctly distinguishing between eutectic and deep eutectic mixtures and determining the operating temperature and composition ranges at which these mixtures remain in a liquid state during use.

The primary experimental method for investigating the SLE of these systems is differential scanning calorimetry (DSC), which detects thermal events such as melting and crystallization, providing melting temperatures and enthalpies for pure compounds and their mixtures. These data can be applied to the ideal phase behavior (eqn (2)) to predict phase behavior. Real phase behavior is characterized by determining activity coefficients from experimental SLE diagrams. Additionally, SLE can be predicted at the bulk level using semi-empirical methods and equation-of-state models such as the Statistical Associating Fluid Theory (SAFT), Perturbed Chain SAFT (PC-SAFT), Conductor-like Screening Model (COSMO), COSMO for realistic solvents (COSMO-RS), and Segment Activity Coefficients (COSMO-SAC).^33^

Based on deviations from ideal behavior, Cherniakova *et al.*^32^ proposed a classification of binary mixtures according to their eutecticity: normal eutectic solvents (NES) exhibit quasi-ideal behavior for both components; negative deviations in one or both components lead to the formation of a deep eutectic solvent (DES); and positive deviations characterize a shallow eutectic solvent (SES). These three types of phase behavior all belong to the broader class of eutectic solvents (ES).

## 4. History of thymol-based DESs

Terpenes and terpenoids, such as menthol, thymol, and citronellol, are derived from essential oils and offer a renewable source for creating DESs with unique characteristics. Terpene-based DESs have emerged as a notable advancement in green chemistry, leveraging the natural abundance and unique properties of terpenes to create environmentally friendly solvents. The concept of using terpenes in DESs gained traction in the early 2010s, with research highlighting their potential as sustainable alternatives to traditional solvents.^34^ These DESs are formed by combining terpenes with various HBDs and HBAs. Terpene-based DESs offer several advantages, including low toxicity, high biodegradability, and the ability to be customized for specific applications by adjusting their composition. Their natural origin and eco-friendly characteristics make them particularly attractive for sustainable industrial processes and green chemistry applications. The primary types include menthol-based DESs, which are effective in extraction and separation processes due to menthol's ability to dissolve a broad range of substances.^32,35^

Thymol-based DESs have attracted considerable interest in recent years owing to their unique physicochemical properties and wide-ranging applications. Thymol, a natural phenolic monoterpenoid derived from thyme oil, was first explored as a DES component in 2018. In that year, Martin *et al.*^34^ prepared and characterized hydrophobic binary DESs composed of thymol as the HBA and several long-chain monocarboxylic acids, namely caprylic, capric, lauric, myristic, palmitic, and stearic acids, as HBDs. Their work included the determination of SLE phase diagrams, density, viscosity, solvatochromic parameters at the eutectic composition, and mutual solubilities with water. Also in 2018, Schaeffer *et al.*^36^ employed thymol-based hydrophobic DESs (thymol : lauric acid, thymol : myristic acid, thymol : palmitic acid, and thymol : stearic acid) for the selective extraction and separation of Cu(ii) ions from other transition metals in mildly acidic aqueous media. In parallel, Makoś *et al.*^37^ synthesized thymol : capric acid and thymol : 10-undecylenic acid DESs, examined their physicochemical characteristics, and demonstrated their applicability as extraction solvents in ultrasound-assisted dispersive liquid–liquid microextraction for the separation and enrichment of polycyclic aromatic hydrocarbons from complex aqueous matrices. In the following years, a large number of thymol-based binary and ternary DESs were prepared. Binary systems involve thymol combined with a single HBD, such as menthol, camphor, or fatty acids, while ternary systems incorporate a third component, such as alcohols or organic acids, to further tailor the solvent's properties.^31^ The versatility of thymol-based DESs has led to their application in various fields, including pharmaceuticals, where they enhance drug solubility and stability, in the extraction and purification of bioactive compounds, and as green solvents in organic synthesis.^38^ Thymol-based DESs are also particularly effective in applications requiring antibacterial properties, as thymol itself possesses strong antimicrobial activity, making these solvents ideal for use in pharmaceutical formulations, surface disinfectants, and bioactive compound extraction.^39^Fig. 3 shows some of the properties and benefits of thymol as a DESs constituent. One of the key advantages of thymol-based DESs is their low toxicity and biodegradability, making them safer alternatives to conventional organic solvents. Additionally, their hydrophobic nature allows for the dissolution of a wide range of non-polar compounds, which is advantageous in applications where traditional hydrophilic DESs may be less effective. Most of the thymol-based DESs synthesized so far belong to type V, characterized by their nonionic nature, which offers distinct advantages over ILs and other types of DESs. These advantages include lower toxicity, enhanced biodegradability, and greater environmental compatibility. These characteristics position thymol-based DESs as promising tools in sustainable chemistry, offering eco-friendly and efficient solutions across multiple industries.

**Fig. 3:**
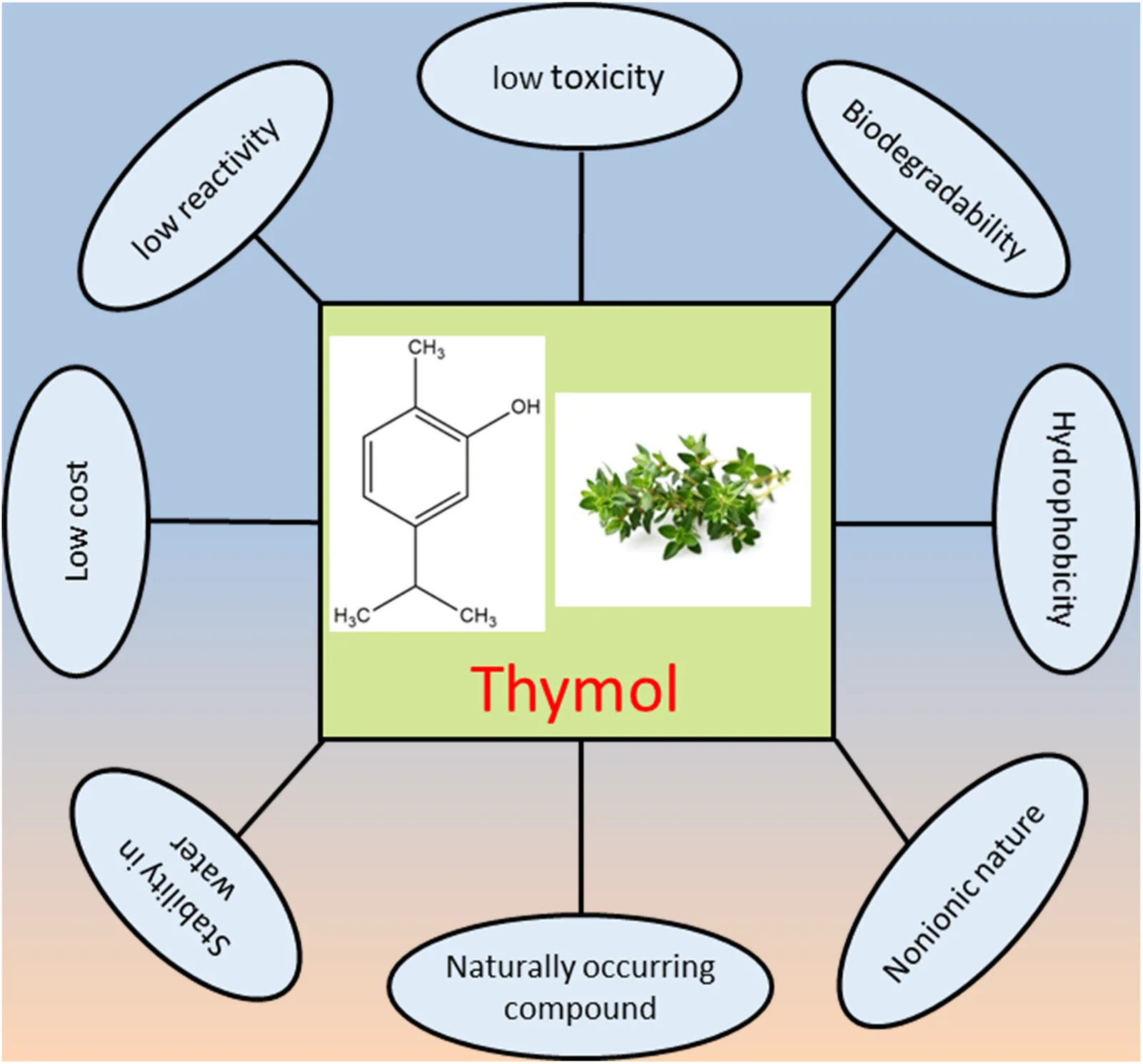
Advantages of thymol as DESs constituent.

Fig. 4 shows the number of publications over the past decade obtained from the *Web of Science* data based on the search for the terms “deep eutectic solvent”, “hydrophobic deep eutectic solvent”, “natural deep eutectic solvent” and {“thymol” and “deep eutectic solvent”} within the abstract field. Given that the main goal of this work is the study of thymol-based DESs, and considering that some of the articles retrieved from the initial search may not specifically focus on thymol-based DESs, while other relevant studies might not have been captured by the search, a more precise and comprehensive strategy was employed. This included conducting searches across multiple databases, examining the reference lists of the articles identified, and thoroughly reviewing the content of these articles to ensure that all relevant thymol-based DES studies were considered. In total, to our knowledge, there are 215 papers on the thymol-based DESs in the literature, which are distributed over the years as: 3 article in 2018, 8 in 2019, 12 in 2020, 25 in 2021, 40 in 2022, 47 in 2023, 41 in 2024 and 39 in 2025.

**Fig. 4:**
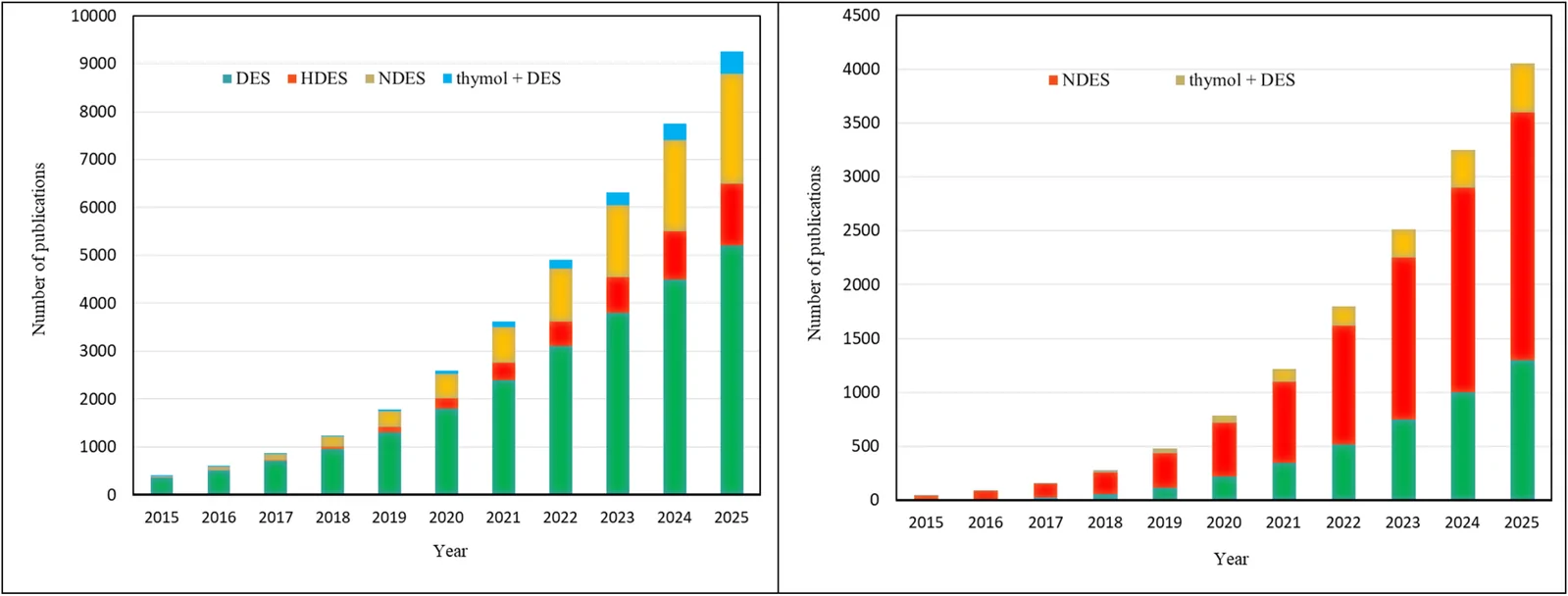
Number of publications on the search requests “deep eutectic solvent”, “hydrophobic deep eutectic solvent”, “natural deep eutectic solvent”, and “thymol” + “deep eutectic solvent” within last 10 years according to topic field of web of science.

An extensive review of the existing literature indicates that, despite the growing significance of thymol-based DESs, a comprehensive and systematic review covering all aspects of this field is still lacking. In response to this gap, the present paper aims to provide an in-depth review for a detailed analysis of binary and ternary thymol-based DESs, including the various thymol-based DESs synthesized to date, preparation methods, phase diagrams, thermophysical properties, stability in aqueous media, and their applications. This review is organized into nine comprehensive sections. Section 1 provides an introduction to the fundamental concepts of the field. Sections 2 and 3 detail the various preparation methods for DESs and the significance of solid–liquid equilibria (SLE) phase diagrams. The historical development and systematic classification of binary thymol-based DESs are discussed in Sections 4 and 5, respectively. Section 6 serves as a core technical chapter, examining the molar ratios, compositional diversity, physicochemical properties, and water stability of binary systems, with specific subsections (6.1–6.8) dedicated to different hydrogen bond donors/acceptors such as carboxylic acids, terpenoids, and nitrogen-containing heterocycles. The practical utility of these solvents across diverse fields, including analytical chemistry, environmental remediation, and biomedical engineering, is reviewed in Section 7. Section 8 extends the scope to ternary thymol-based DESs, exploring their unique compositions and emerging applications. Finally, Section 9 summarizes the key findings, addresses current challenges, and proposes strategic directions for future research.

## 5. Classification of binary thymol-based DESs

Thymol, a phenolic monoterpenoid, is a natural product extracted from the plant species *Thymus kotschyanus* and *Achillea ageratum*, and constitutes approximately 50% of the main composition of thyme oil. Its unique molecular architecture, combining a hydrophobic aromatic terpenoid backbone with a hydrogen-bond-active phenolic group, dictates its distinctive behavior as a DES-forming component. Thymol possesses several structural features that distinguish it from conventional HBDs and HBAs and explain its unique behavior as a component of DESs. Chemically, thymol is a naturally occurring phenolic monoterpenoid (2-isopropyl-5-methylphenol) consisting of a hydrophobic aromatic ring substituted with a phenolic hydroxyl group and two non-polar alkyl groups (methyl and isopropyl). The phenolic OH group provides HBD/HBA capabilities, enabling thymol to form diverse intermolecular interactions with the complementary components of DES. Although thymol's hydrogen-bonding capability is lower than that of highly polar HBDs/HBAs (such as sugars, polyols, amides, and carboxylic acids), the unique combination of its phenolic functional group and hydrophobic aromatic/terpenoid structure establishes a balance between hydrogen-bonding potential and non-polar interactions. This amphiphilic molecular architecture enables thymol to form hydrophobic Type V DESs, in which the melting point depression arises from the synergistic combination of hydrogen bonding, van der Waals interactions, and disrupted crystal packing, rather than from ionic interactions.^38,40,41^ Compared with IL-based hydrophobic solvents and many hydrophilic DESs, thymol-based DESs generally exhibit low water miscibility, negligible vapor pressure, simple preparation procedures, and favorable biodegradability, while maintaining strong affinity toward nonpolar and moderately polar compounds. These properties have enhanced their application in the liquid–liquid extraction of organic pollutants, bioactive compounds, metal ions, and pharmaceutical molecules from aqueous media, as well as in antimicrobial formulations and sustainable separation processes.^42,43^ Furthermore, compared to other hydrophobic DESs based on menthol, fatty acids, or other terpenoid compounds, thymol-based systems offer superior performance due to the inherent antioxidant and antimicrobial activities of the phenolic moiety, enabling the development of multifunctional solvents that combine efficient extraction performance with biological activity.^39^

The various materials that have been used so far to form DES with thymol can be divided into the following 7 categories: including: (i) carboxylic acids, (ii) amides/amines/nitrogen-containing heterocycles, (iii) alcohols/diols, (iv) terpenoids, (v) quaternary ammonium salts, (vi) phenolic compounds, and (vii) other functional compounds (Table 1 and Fig. 5).

**Table 1:** A classification of various materials used so far to form binary DESs with thymol

Material	Ref.	Material	Ref.
**Carboxylic acid**			
Methanoic acid (formic acid)	44	Decanoic acid (capric acid)	26, 34, 36, 37 and 45–95
Ethanoic acid (acetic acid)	44, 68, 72, 96 and 97	Undecanoic acid	92
2-Hydroxypropanoic acid (lactic acid)	98–102	10-Undecylenic acid	37 and 103
Propenoic acid (acrylic acid)	104 and 105	Dodecanoic acid (lauric acid)	34, 36,75,85,88,48,50,52,57–59, 64 and 69
4-Oxopentanoic acid (levulinic acid)	80,100 and 106–109	Tetradecanoic acid (myristic acid)	34,36, 88, 104 and 105
Hexanoic acid (caproic acid)	49, 50, 104, 105, 108, 110–112, 57, 59, 61, 68, 77, 78, 81 and 84	Hexadecanoic acid (palmitic acid)	34, 36 and 88
Heptanoic acid (enanthic acid)	26, 49, 61, 73, 79, 81, 84, 89, 112 and 113	Octadecanoic acid (stearic acid)	34, 36, 88, 104 and 105
Octanoic acid (caprylic acid)	26, 34, 56–64, 68, 36, 69, 71–73, 77–79, 81, 84, 85, 43, 88–92, 95, 104, 105, 108, 110, 46, 111, 112, 114–121, 48, 122, 49, 50, 52 and 55	Octadecenoic acid (oleic acid)	67, 96, 104, 105 and 123
Nonanoic acid (pelargonic acid)	26, 49, 81, 84, 89, 104, 105, 124, 55, 56, 59–62, 73 and 79	Octadecadienoic acid (linoleic acid)	96
Nonanedioic acid (azelaic acid)	111 and 125	2-Hexyldecanoic acid	126
Hydrocinnamic acid	127	Phenylacetic acid	128
Citric acid	129 and 130	Butyric acid	68 and 119
Pyruvic (2-oxopropanoic) acid	131	Methacrylic acid	132
Polyacrylic acid	133		
Maleic acid	89		

**Amides/amines/nitrogen-containing heterocycle**			
Urea	128 and 129	4-(Dimethylamino)benzaldehyde	134
Acetamide	135	1,10-Phenanthroline	136 and 137
Acetanilide	135	Lidocaine	108 and 138–142
Triethylenetetramine HCl	143 and 144	Atropine	138
Tetraethylpentamine HCl	144	Caffeine	127
Monoethanolamine	145	1-(*o*-Tolyl)thiourea	146
*N*-Methylethanolamine	145	*N*,*N*-Dimethylformamide	147

**Alcohol/diol**			
Ethylene glycol	133, 148 and 149	Tetradecanol (myristyl alcohol)	150–152
Hexafluoroisopropanol	153	Hexadecanol	130 and 150
Isobutanol	43	Octadecanol	150
Octanol	55,90, 124 and 154	Benzyl alcohol	43, 55 and 124, 154 and 155
Decanol	55, 122 and 147	2-Hydroxyethyl methacrylate	156
1,2-Decanediol	138	Polyethylene glycol 400 (PEG400)	43
1-Dodecanol (lauryl alcohol or laurinol)	44 and 157	Polyethylene glycol 200 (PEG200)	133
1,4-Butanediol	71		

**Terpenoid** (**oxygenated derivative of terpenes)**			
Borneol	111 and 158–163	Sobrerol	159
Fenchol	164	Camphor	26, 37, 154, 159, 160, 162–168, 47, 169–178, 53, 65, 66, 80, 108, 113 and 122
Terpineol	161 and 164	Carvone	101, 173, 176, 177, 179 and 180
β-Citronellol	181	Cineole	178–180
Menthol	26, 38, 71, 74, 82, 85, 91, 100, 101, 103, 106, 108, 43, 112, 113, 117, 118, 120, 122, 124, 131, 138, 147, 45, 152, 154, 159, 161–164, 166, 167, 169, 51, 171, 175, 182–189, 54, 190–199, 55, 200–209, 65, 210, 211, 66 and 69	Linalool	101
Geraniol	101	Carvacrol	212

**Quaternary ammonium salt**			
Tetrabutylammonium bromide (TBABr)	67	Methyltrioctylammonium chloride (MTOACl)	67, 121 and 213
Tetrabutylammonium chloride (TBACl)	70, 71 and 214	Tetraethylammonium chloride (TEACl)	215
Tetramethylammonium chloride (TMACl)	216	1,1,3,3-Tetramethylguanidine chloride (TMGHCl)	213
Choline chloride (ChCl)	55, 71, 102, 213 and 217–225	*N*,*N*-Dimethyl-*N*,*N*-didodecylammonium chloride (DDDACl)	226
Trimethylglycine (betaine)	227	Tetraheptylammonium bromide (THABr)	43
Benzyltriethylammonium chloride (BTEACl)	121, 221–223, 228 and 229	Aliquat 336	230 and 231

**Phenol and phenolic derivatives**			
Phenol	232	2,6-Dimethoxyphenol (syringol)	232 and 233
2-Chlorophenol	65	Eugenol	127, 175 and 234
2,6-Xylenol	197	Phenyl salicylate (salol)	26 and 154
Coumarin	91, 103, 172, 174, 196, 211, 235–240, 108, 113, 127, 138, 164, 168, 170 and 171	Flavone	241
Vanillin	72, 111 and 242–246	Flavanone	241
6-Methylcoumarin	236, 247 and 248	benzophenone	249

**Others**			
Water	128 and 129	Trioctylphosphine oxide (TOPO)	108, 127 and 250
Triisobutylphosphine sulfide	251	Bis(2-ethylhexyl) phosphoric acid	252
Poly(methacrylic acid-*co*-ethylene glycol dimethacrylate)	132	3-Hydroxyhexanoate	253
Butyl lactate	253	Methyl salicylate	253
4-Methoxybenzaldehyde	246	2,4,5-Trimethoxybenzaldehyde	246
4-Methylbenzaldehyde	246		

**Fig. 5:**
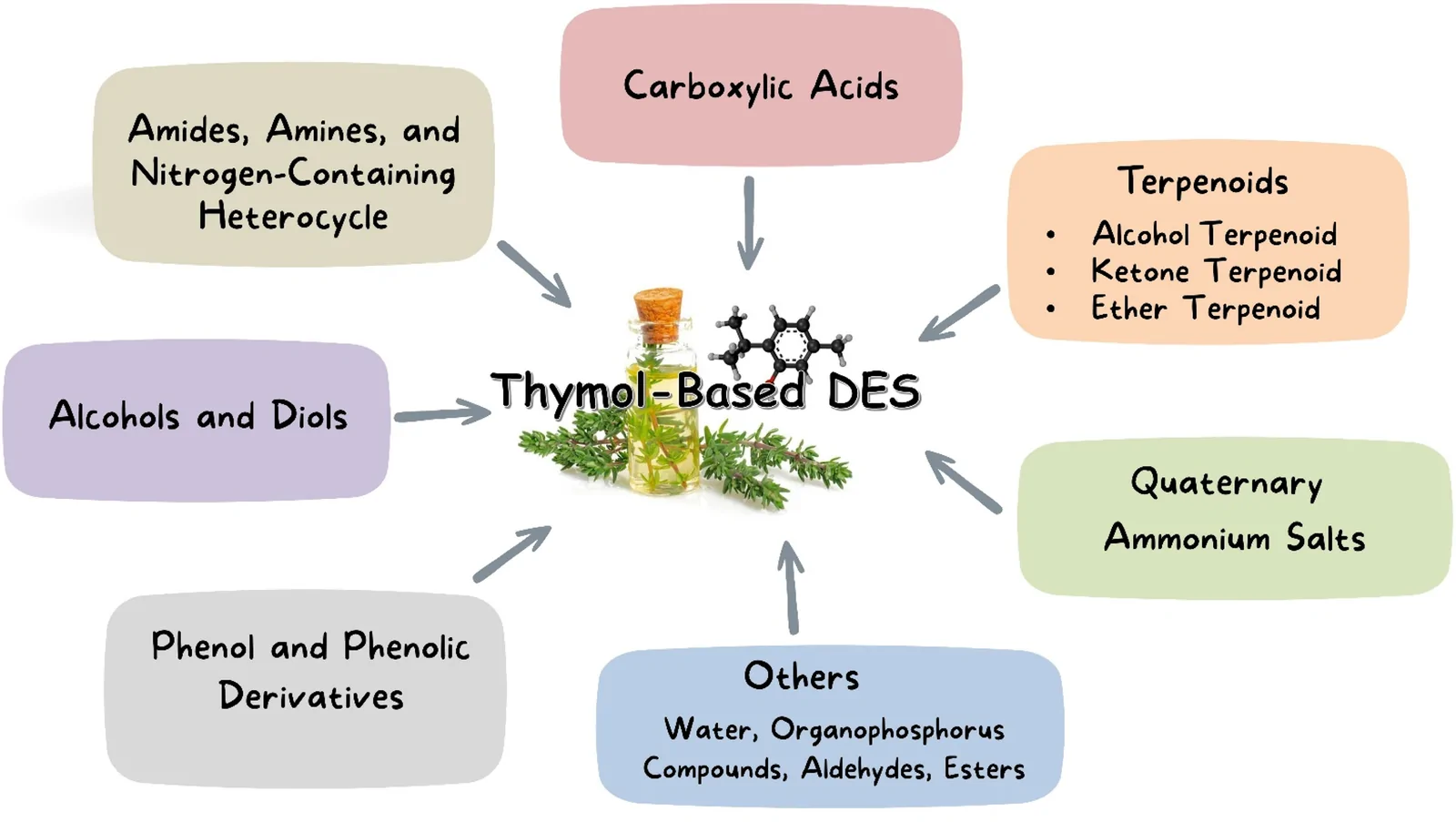
A classification of materials used so far to form DES with thymol.

Beyond classification based on composition, thymol-based DESs can also be categorized according to the dominant intermolecular interactions responsible for their formation and properties. The most common category comprises hydrogen-bonding-based DESs; in this group, the phenolic OH group of thymol interacts with amides, carboxylic acids, alcohols, or other HBDs/HBAs, resulting in a depression of the melting point and the formation of a stable eutectic mixture.^254^ These systems dominate the literature because thymol, while maintaining hydrophobicity, provides an effective site for hydrogen bond formation.^38^ The second category comprises systems in which acid–base interactions play a dominant role, and proton transfer or partial charge delocalization contributes to the formation of these solvents. In these systems, acidic components (such as organic acids) can interact with thymol both through hydrogen bonding and *via* acid–base interactions involving the phenolic group. The third category comprises the hydrophobic DESs, characterized by dispersion forces and hydrophobic interactions. In these systems, thymol is combined with non-polar or weakly polar molecules, such as terpenes, hydrocarbons, or long-chain fatty compounds. Although their overall hydrophobic character is governed by the nonpolar portions of the constituent molecules, molecular-level studies indicate that hydrogen bonding can remain an important organizing interaction, together with dispersion, van der Waals, and other noncovalent interactions.^255^ Recently, multifunctional thymol-based DESs incorporating nanoparticles, active molecules, or other functional components have emerged, in which additional interactions, such as coordination or surface interactions, enhance their performance. In the published literature on thymol-based DESs, the molecular-level interactions responsible for their formation have not yet been comprehensively elucidated through a combination of experimental and theoretical approaches. Although hydrogen bonding, van der Waals forces, hydrophobic interactions, and, in some cases, acid–base interactions have been proposed as key contributors to the formation and stabilization of these systems, the relative contribution and specific role of each interaction remain insufficiently understood.^241^ This knowledge gap limits the rational design, classification, and optimization of thymol-based DESs for targeted applications. Future studies should therefore integrate advanced experimental techniques, including spectroscopic methods (*e.g.*, FT-IR, Raman, and NMR spectroscopy) to identify structural changes and intermolecular interactions, with theoretical approaches such as MD simulations, quantum chemical calculations, and COSMO-RS modeling to provide molecular-level insights into DES formation mechanisms. Such combined experimental–computational investigations will enable a more accurate classification of thymol-based DESs according to the dominant interactions involved and facilitate the development of next-generation DESs with predictable physicochemical properties and enhanced performance in separation, extraction, and other technological applications.^241,256^

## 6. Molar ratio and compositional diversity, solid–liquid equilibria phase behavior, physicochemical properties, water stability and hydrophobicity, and preparation methods in binary thymol-based DESs

Table 2 categorizes the materials used to form thymol-based DESs by their respective classes, specifying information regarding the molar ratio of HBD and HBA, physicochemical properties, water stability, and preparation methods. As can be seen from Table 2, while the “heating and stirring” method remains the predominant approach for preparing thymol-based DESs, alternative techniques have also been reported. These include “stirring at room temperature (without heating)”, “heating without stirring”, “heating, stirring, and sonication”, “ultrasonication and heating”, “ultrasonication without heating”, “vacuum evaporation (without using any solvent)”, “addition of both compounds to a container (without heating, stirring or another external factor)”, and “*in situ* formation” methods. External heating (without stirring) is usually required for the *in situ* formation of thymol-based HDES. However, Pochivalov *et al.*^49^ prepared thymol : fatty acid HDESs by pH-adjustment-based *in situ* method without the need for heating. In this approach, an alkaline solution of the DES precursors (in this example, thymol and hexanoic/heptanoic/octanoic/nonanoic/decanoic acids) is prepared and mixed with the aqueous sample containing extractive analytes. In the alkaline solution, both thymol and fatty acids exist in their anionic, hydrophilic forms, making them highly soluble in water and forming a homogeneous mixture with the aqueous sample. Then, DES Formation was triggered by acidification. As the pH drops, the hydrophilic anionic forms of the DES precursors become protonated, transforming into their molecular (non-ionized) forms. These molecular forms have limited solubility in aqueous media. This change in solubility causes fine droplets of the HDES phase to appear throughout the entire volume of the extraction system. This simultaneous formation of HDES microdroplets and extraction of analytes allows for rapid mass transfer. The pH-adjustment *in situ* preparation of HDESs avoids the need for heating and auxiliary energy sources (like microwave or ultrasound radiation, or vortex mixing) allowing the extraction of thermally unstable analytes in aqueous media by a simple, rapid, low-cost, and high-efficiency approach.^49^

**Table 2:** Binary thymol-based eutectic mixtures: classification and information regarding the molar ratio of constituents, physicochemical properties, water stability, and preparation method

DES	HBD	HBA	Molar ratio (thymol : another compound)	SLE phase diagram	Experimental physicochemical properties	Water stability	Preparation methods
**Class III**							
*Thymol : quaternary ammonium salt*							
Thymol : etrabutylammonium bromide (TBABr)	Thymol	TBABr	3 : 1 ^67^	✗	✗	Stable^67^	Heating and stirring^67^
Thymol : tetramethylammonium chloride (TMACl)	Thymol	TMACl	(1 : 1, 2 : 1, 3 : 1, 4 : 1, and 5 : 1)^216^	✗	✗	Stable^216^	Heating and stirring^216^
Thymol : tetraethylammonium chloride (TEACl)	Thymol	TEACl	(1 : 1, 2 : 1, 3 : 1, 4 : 1, and 5 : 1)^215^	✗	✗	Stable^215^	Heating and stirring^215^
Thymol : tetrabutylammonium chloride (TBACl)	Thymol	TBACl	(2 : 1 and 4 : 1),^70,71^ (3 : 1)^214^	✗	✓ (density (25–70 °C), viscosity (25–70 °C), and melting point/glass transition for water-saturated DES with 4 : 1 molar ratio)^70^	Stable^70,214^^l^	Heating and stirring^70,71,214^
Thymo : choline chloride (ChCl)	Thymol	ChCl	4 : 1,^55^ (1 : 1, 2 : 1, 3 : 1, and 4 : 1),^220^ 2 : 1,^221^ 2 : 1,^222^ 2 : 1,^102^ (1 : 1, 2 : 1, 3 : 1, and 4 : 1),^71^ 5 : 1,^213^ 4 : 1,^223^ (4 : 1 and 1 : 3),^224^ 2 : 1,^225^ 4 : 1,^217^ 4 : 1,^218^ 1 : 1 ^219^	✗	✓ (density and water content for 4 : 1 molar ratio at room temperature)^220^	Stable^55,213,217,218,220–223,225^	Heating and stirring^55,71,102,213,217–225^
					✓ (decomposition temperature and contact angle of water (25 °C) for 5 : 1 molar ratio)^213^	Unstable^102,220^^h^	Sonication and heating^224^
Thymol : trimethylglycine (betaine)	Thymol	Betaine	(The whole composition range was investigated, and the eutectic point was specified as 3 : 1 molar ratio (*x*^E^_thy_ = 0.75, *T*^E^ = −15 °C))^226^	✓ ^226^	✓ (density and water content for 3 : 1 molar ratio at 25 °C)^226^	Stable^226^	Heating and stirring^226^
Thymol : benzyltriethylammonium chloride (BTEACl)	Thymol	BTEACl	(1 : 1, 2 : 1, 3 : 1, and 4 : 1),^221^ (1 : 1, 2 : 1, 3 : 1, and 4 : 1),^222^ (1 : 1, 2 : 1, 3 : 1, and 4 : 1),^223^ (1 : 1, 2 : 1, 3 : 1, and 4 : 1),^228^ (4 : 1, 5 : 1, 6 : 1, and 7 : 1),^121^ 2 : 1 ^229^	✗	✓ (density and viscosity for 4 : 1 molar ratio at 25 °C)^222^	Stale^121,221–223,228^	Heating and stirring^121,221–223,228,229^
					✓ (density at room temperature, and melting point for 4 : 1 molar ratio)^121^		
Thymol : methyltrioctylammonium chloride (MTOACl)	Thymol	MTOACl	(3 : 1, 4 : 1, 5 : 1, 6 : 1, 7 : 1, and 8 : 1),^67^ 2 : 1,^213^ 4 : 1 ^121^	✗	✓ (decomposition temperature and contact angle of water (25 °C) for 2 : 1 molar ratio)^213^	Stable^67,121,213^	Heating and stirring^67,121,213^
					✓ (density at room temperature, and melting point for 4 : 1 molar ratio)^121^		
Thymol : 1,1,3,3-tetramethylguanidine chloride (TMGHCl)	Thymol	TMGHCl	2 : 1 ^213^	✗	✓ (decomposition temperature and contact angle of water (25 °C) for 2 : 1 molar ratio)^213^	Stable^213^	Heating and stirring^213^
Thymol : *N*,*N*-dimethyl-*N*,*N*-didodecylammonium chloride (DDDACl)	Thymol	DDDACl	(The whole composition range was investigated, and the eutectic point was specified as 2 : 1 molar ratio (*x*^E^_thy_ = 0.67, *T*^E^ = −20 °C))^226^	✓ ^226^	✓ (density and water content for 3 : 1 molar ratio at 25 °C)^226^	Stable^226^	Heating and stirring^226^
Thymol : tetraheptylammonium bromide (THABr)	Thymol	THABr	1 : 1 ^43^	✗	✗	Stable^43^	Heating and stirring^43^
Thymol : aliquat 336	Thymol	Aliquat 336	1 : 1,^230^ (the whole composition range was investigated, and the eutectic point was specified as 1 : 1.22 molar ratio (*x*^E^_thy_ = 0.45, *T*^E^ = −80 °C))^231^	✓ ^231^^n^	✓ (density, viscosity, and refractive index for 1 : 1.22 molar ratio, at different temperatures)^231^	Stable^230,231^	Heating and stirring^230,231^

**Class V**							
*Thymol : carboxylic acid*							
Thymol : methanoic acid (formic acid)	Formic acid	Thymol	1 : 1 ^44^	✗	✗	Stable^44^	Heating and stirring^44^
Thymol : ethanoic acid (acetic acid)	Acetic acid	Thymol	(1 : 1, 1 : 2, 1 : 3, 1 : 4 and 2 : 1),^44^	✗	✗	Stable^44,72^^b^	Heating and stirring^44,68,72,96,97^
			(1 : 1, 5 : 1, and 1 : 10),^96^ a 1 : 3,^68^ 1 : 1,^72^ (1 : 1, 1 : 2, 1 : 3, and 1 : 4)^97^			Unstable^96^^c^	
Thymol : 2-hydroxypropanoic acid (lactic acid)	Lactic acid	Thymol	1 : 3,^100^ (2 : 1, 1 : 1, 1 : 2, 1 : 3, and 1 : 4)^98^	✗	✓ )density for 1 : 3 molar ratio^100^	Stable^98–100,102^	Heating and stirring^98–102^
			1 : 2,^99^ 2 : 1,^101^ (1.2 : 1, 1 : 1.6, 1 : 3, and 1 : 3.1)^102^		✓ (density, viscosity, and surface tension for 2 : 1 molar ratio at 25 °C)^101^		
Thymol : propenoic acid (acrylic acid)	Acrylic acid	Thymol	1 : 1,^104^ 1 : 1 ^105^	✗	✗	Stable^104,105^	Heating and stirring^104,105^
Thymol : 2-oxopropanoic acid (pyruvic acid)	Pyruvic acid	Thymol	1 : 1 ^131^	✗	✗	Stable^131^	Heating and stirring^131^
Thymol : maleic acid	Maleic acid	Thymol	2 : 1 ^89^	✗	✗	Stable^89^	Heating and stirring^89^
Thymol : methacrylic acid	Methacrylic acid	Thymol	1 : 1 ^132^	✗	✗	—	Heating and stirring^132^
Thymol : butanoic acid (butyric acid)	Butyric acid	Thymol	1 : 3,^68^ 1 : 1 ^119^	✗	✓ (density (25 °C), vicosity (25 °C), and melting point for 1 : 1 molar ratio)^119^	Stable^119^	Heating and stirring^68,119^
Thymol : 4-oxopentanoic acid (levulinic acid)	Levulinic acid & thymol	Levulinic acid & thymol	(2 : 1, 1 : 1, 1 : 2, 1 : 3, and 1 : 4),^100^ (2.5 : 1, 2.0 : 1, 1.5 : 1, 1 : 1, 1 : 1.5, 1 : 2.0, and 1 : 2.5),^106^ (1 : 1 and 1 : 2),^107^ (1 : 3, 1 : 2, 1 : 1, and 2 : 1),^108^ 1 : 1 ^80^	✗	✓ (density for 1 : 3 molar ratio)^100^	Stable^80,100,108,109^	Heating and stirring^100,106–108^
					✓ (density, viscosity, thermal conductivity, and refractive index for 1 : 1 molar ratio at different temperatures, and water content at room temperature)^106^		Stirring at room temperature^80^
					✓ (decomposition temperature and glass transition for 1 : 1 molar ratio)^80^		*In situ* formation^109^
Thymol : hexanoic acid (caproic acid)	Hexanoic acid & thymol	Hexanoic acid & thymol	1 : 1,^49^ 1 : 1,^104^ 1 : 1,^57^ 1 : 2,^59^ (2 : 1, 1 : 1, and 1 : 2),^110^ (2 : 1, 1 : 1, 1 : 2, and 1 : 3),^50^ 1 : 2,^6^ 1 1 : 3,^68^ 1 : 1,^108^ 1 : 2,^77^ (3 : 1, 2 : 1, 1 : 1, 1 : 2),^78^ 1 : 1,^81^ 1 : 1,^84^ 1 : 1,^105^ (3 : 1, 2 : 1, 1 : 1, 1 : 2, and 1 : 3),^111^ 1 : 2 ^112^	✗	✓ (density for 2 : 1, 1 : 1, and 2 : 1 molar ratios)^110^	Stable^49,50,57,61,77,78,81,84,104,105,108,112^	Heating and stirring^50,57,59,61,68,77,81,84,104,105,108,110–112^
					✓ (density and viscosity for 1 : 1 molar ratio at 20 °C)^49^		Ultrasonication and heating^78^
							*In situ* formation^49^
Thymol : heptanoic acid (enanthic acid)	Heptanoic acid & thymol	Heptanoic acid & thymol	1 : 1,^49^ (3 : 1, 2 : 1, 1 : 1, 1 : 2, and 1 : 3),^26^ 1 : 1,^113^ 1 : 2,^61^ 1 : 1,^149^ (3 : 1, 2 : 1, 1 : 1, 1 : 2, and 1 : 3),^73^ (1 : 1, 2 : 1, and 1 : 2),^79^ 1 : 1,^81^ 1 : 1,^84^ 2 : 1,^89^ 1 : 2 ^112^	✗	✓ (density and viscosity for 1 : 1 molar ratio at 20 °C)^49^	Stable^26,49,61,73,81,84,89,112,113^	*In situ* formation^26,49^
					✓ (melting point and viscosity (25 °C) for 1 : 1 molar ratio, and polarity and density for 3 : 1, 2 : 1, 1 : 1, 1 : 2, and 1 : 3 molar ratios at 25 °C)^26^		Heating and stirring^61,73,79,81,84,89,112,113,149^
Thymol : octanoic acid (caprylic acid)	Caprylic acid & thymol	Caprylic acid & thymol	(1 : 1, 1 : 1.9, and 1 : 4),^104^ (2 : 1, 1 : 1, and 1 : 2),^110^ 1 : 1,^49^ 1 : 1,^57^ 2 : 1,^50^ 1 : 1.5,^59^ 1 : 1,^26^ (the whole composition range was investigated, and the eutectic point was specified as 1 : 1.857 molar ratio (*x*^E^_thy_ = 0.35, *T*^E^ = −2.33 °C)),^34^ 21 : 29,^55^ 1 : 1,^48^ 1 : 1.38,^52^ (1 : 6, 1 : 5, 1 : 4, 1 : 3, 1 : 2, 1 : 1, 2 : 1, 3 : 1, and 4 : 1),^60^ (1 : 5, 1 : 4, 1 : 3, 1 : 2, 1 : 1, 2 : 1, and 3 : 1),^62^ 2 : 1,^114^ 1 : 1,^115^ 1 : 1,^56^ 2 : 1,^58^ 1 : 1.94,^116^ 1 : 1,^63^ 1 : 1.38,^36^ (the whole composition range was investigated, and the eutectic point was specified as 1 : 2.004 molar ratio (*x*^E^_thy_ = 0.3329, *T*^E^ = 1.25 °C,^46^ 1 : 2,^61^ 1 : 1,^92^ 1 : 1.04,^95^ 1 : 1.02,^64^ 1 : 1,^43^ 1 : 1.94,^117^ 1 : 1,^118^ 1 : 3,^68^ 1 : 1,^119^ 1 : 1,^69^ 1 : 1,^71^ 1 : 1,^120^ 1 : 2,^121^ 1 : 1,^72^ 1 : 1,^108^ 1 : 1,^73^ 1 : 2,^77^ 2 : 1,^78^ (1 : 1, 2 : 1, and 1 : 2),^79^ (1 : 1, 1 : 2, and 2 : 1),^81^ (1 : 3, 1 : 2, 1 : 1, 2 : 1, and 3 : 1),^84^ 1 : 1,^85^ 1 : 1,^88^ (1 : 1, 1 : 1.86, and 1 : 4),^105^ 1 : 1,^111^ (2 : 1, 1 : 1, and 1 : 2),^89^ 1 : 2,^90^ 1 : 1,^122^ 1 : 2,^112^ 1 : 3 ^91^	✓ ^34^	✓ (density for 2 : 1, 1 : 1, and 2 : 1 molar ratios)^110^	Stable^26,34,36,43,46,48–50,55–62,64,69,71–73,77,78,81,84,85,89,90,92,104,105,108,112,114–121^	Heating and stirring,^34,36,43,46,48,50,52,55–64,68,69,71–73,77,79,81,84,85,88–92,95,104,105,108,110–112,114–122^
				✓ ^46^	✓ (density and viscosity for 1 : 1 molar ratio at 20 °C)^49^		Ultrasonication and heating^78^
					✓ (melting point, viscosity, polarity and density for 1 : 1 molar ratio at 25 °C)^26^		*In situ* formation^26,49^
					✓ (density and viscosity for 1 : 1.375 molar ratio at different temperatures)^34^		
					✓ (density, viscosity, and surface tension for 1 : 1 molar ratio at different temperatures)^48^		
					✓ (density at 45 °C and melting point)^58^		
					✓ (density and viscosity for 1 : 1.38 molar ratio at 60 °C, and melting point for this molar ratio)^36^		
					✓ (density, speed of sound, isobaric molar heat capacity, refraction index, static permittivity, surface tension, and kinematic viscosity for 1 : 2, 1 : 1, and 2 : 1 molar ratios at different temperatures)^46^		
					✓ (density (25 °C), vicosity (25 °C), and melting point for 1 : 1 molar ratio)^119^		
					✓ (density at room temperature, and melting point for 1 : 2 molar ratio)^121^		
					✓ (decomposition temperature and melting point for 1 : 1 molar ratio)^79,81^		
					✓ (electrical conductivity (room temperature) and viscosity (4 °C) for 1 : 1 molar ratio)^85^		
					✓ (polarity, density, and viscosity for 1 : 3 molar ratio at 25 °C)^91^		
Thymol : nonanoic acid (pelargonic acid)	Pelargonic acid & thymol	Pelargonic acid & thymol	1 : 1,^104^ 1 : 1,^49^ 1 : 1,^59^ 1 : 1,^26^ 1 : 1,^55^ 1 : 5,^60^ 1 : 4,^62^ 1 : 1,^56^ 1 : 2,^61^ 1 : 1,^92^ 2 : 1,^124^ 1 : 1,^149^ 1 : 1,^73^ (1 : 1, 2 : 1, and 1 : 2),^79^ 1 : 1,^81^ 1 : 1,^84^ 1 : 1,^105^ 2 : 1 ^89^	✗	✓ (density and viscosity for 1 : 1 molar ratio at 20 °C)^49^	Stable^26,49,55,56,61,62,73,81,84,89,92,104,105,124^	Heating and stirring^55,56,59–62,73,79,81,84,89,92,104,105,124,149^
					✓ (melting point, viscosity, polarity and density for 1 : 1 molar ratio at 25 °C)^26^		*In situ* formation^26,49^
					✓ (density (25 °C), surface tension (25 °C), and viscosity (50 °C) for 2 : 1 molar ratio) ^124^		
Thymol : nonanedioic acid (azelaic acid)	Azelaic acid & thymol	Azelaic acid & thymol	(15 : 1, 16 : 1, 17 : 1, 18 : 1, 19 : 1),^125^ 17 : 1 ^111^	✗	✓ (density for 15 : 1, 16 : 1, 17 : 1, 18 : 1, 19 : 1 molar ratios and melting point for 17 : 1 molar ratio)^125^	Stable^125^	*In situ* formation,^125^ heating and stirring^111^
Thymol : hydrocinnamic acid	Hydrocinnamic acid	Thymol	1 : 1 ^127^	✗	✗	—	Heating and stirring^127^
Thymol : decanoic acid (capric acid)	Capric acid & thymol	Capric acid & thymol	1 : 1,^49^ 2 : 1,^50^ (1 : 1, 2 : 1, and 1 : 2),^57^ 1 : 1,^59^ 1 : 1,^26^ (the whole composition range was investigated, and the eutectic point was specified as 1 : 1.273 molar ratio (*x*^E^_thy_ = 0.44, *T*^E^ = 13.85 °C)),^34^ 1 : 1,^55^ 1 : 1,^48^ 1 : 1,^52^ 1 : 5,^60^ 1 : 4,^62^ 1 : 1,^56^ 2 : 1,^58^ 1 : 1,^63^ 1 : 1,^36^ (the whole composition range was investigated, and the eutectic point was specified as 1 : 1.228 molar ratio (*x*^E^_thy_ = 0.4488, *T*^E^ = 12.35 °C)),^46^ 1 : 2,^61^ (1 : 1, 2 : 1),^45^ (2 : 1, 1 : 1, 1 : 2),^54^ (1 : 2, 1 : 1, 1.5 : 1, and 2 : 1),^51^ (1.5 : 1, 1 : 1, 1 : 2, and 1 : 3),^37^ (3.44 : 1, 2.29 : 1, 1.15 : 1, 1 : 1.74, and 1 : 2.62),^47^ 1 : 1,^53^ (1 : 1, 1 : 2, and 2 : 1),^75^ 1 : 1,^86^ 1 : 1,^92^ 1 : 1,^93^ (1 : 1, 1 : 1.5, and 1 : 2),^94^ 1.15 : 1,^95^ 1 : 1.273,^64^ (1 : 1, 1 : 2, and 2 : 1),^65^ (1 : 1, 2 : 1, and 1 : 2),^66^ (1 : 2, 1 : 1, 2 : 1, 3 : 1, 4 : 1, and 5 : 1),^67^ 1 : 3,^68^ 1 : 1,^69^ 1 : 1,^70^ 1 : 1,^71^ (1 : 1, 1 : 2, and 2 : 1),^72^ 1 : 1,^73^ (2 : 1, 1 : 1, and 1 : 2),^74^ (2 : 1, 1.5 : 1, 1 : 1, and 1 : 2),^76^ 1 : 2,^77^ 2 : 1,^78^ 1 : 1,^80^ (1 : 1, 2 : 1, and 1 : 2)^79^, 1 : 1,^81^ (1 : 2 and 1.5 : 1),^82^ 1 : 1,^83^ 1 : 1,^84^ 1 : 1,^85^ (1 : 1, 1 : 2, and 2 : 1),^87^ 1 : 1,^88^ 2 : 1,^89^ 1 : 2,^90^ 1 : 1 ^91^	✓ ^34^	✓ (density and viscosity for 1 : 1 molar ratio at 20 °C)^49^	Stable^26,34,36,37,45–51,53,55–58,61,62,64–78,80,81,83–85,87,89,90,92–94^	Heating and stirring^34,36,37,45–48,50–77,79,81–95^
				✓ ^46^	✓viscosity at 25 °C ^57^		Stirring at room temperature^80^
				✓ ^37^^d^	✓ (melting point, viscosity, polarity, and density for 1 : 1 molar ratio at 25 °C)^26^		Ultrasonication and heating^78^
				✓ ^76^^d^	✓ (density and viscosity for 1 : 1 molar ratio at different temperatures)^34^		*In situ* formation^26,49^
					✓ (density, viscosity, and surface tension for 1 : 1 molar ratio at different temperatures)^48^		
					✓ (density at 45 °C and melting point)^58^		
					✓ (density and viscosity for 1 : 1 molar ratio at 60 °C, and melting point for this molar ratio)^36^		
					✓ (density, speed of sound, isobaric molar heat capacity, refraction index, static permittivity, surface tension, and kinematic viscosity for 1 : 2, 1 : 1, and 2 : 1 molar ratios at different temperatures)^46^		
					✓ (melting point and water content (before and after saturation with water) for 1 : 1 and 2 : 1 molar ratios)^45^		
					✓ (density (different temperatures), viscosity (different temperatures), water miscibility (25 °C), and surface tension (25 °C), for 1 : 2, 1 : 1, 1.5 : 1, and 2 : 1 molar ratios)^51^		
					✓ (density and viscosity for 1.5 : 1, 1 : 1, 1 : 2, and 1 : 3 molar ratios at 25 °C, and melting point for 2.33 : 1, 1.5 : 1, 1 : 1, 1 : 2, 1 : 3, and 1 : 4 molar ratios)^37^		
					✓ (density and rheological characteristics for 1 : 1, 1 : 2, and 2 : 1 molar ratios)^75^		
					✓ (melting point for 1 : 1 molar ratio and viscosity of this molar ratio at 25 °C)^86^		
					✓ (interfacial tension between DES (1 : 1 molar ratio) and water)^93^		
					✓ (density (25–70 °C), viscosity (25–70 °C), and melting point/glass transition for water-saturated DES with 1 : 1 molar ratio)^70^		
					✓ (melting point and decomposition temperature for 1 : 1 molar ratio)^72^		
					✓ (density (room temperature) and melting point for 2 : 1, 1.5 : 1, 1 : 1, and 1 : 2 molar ratios)^76^		
					✓ (density and viscosity for 1 : 2 and 1.5 : 1 molar ratios at 20 °C)^82^		
					✓ (electrical conductivity (room temperature) and viscosity (4 °C) for 1 : 1 molar ratio)^85^		
					✓ (density (room temperature), water content (room temperature), and melting point for 1 : 1, 1 : 2, and 2 : 1 molar ratios)^87^		
					✓ (polarity, density, and viscosity for 1 : 1 molar ratio at 25 °C)^91^		
Thymol : undecanoic acid	Undecanoic acid & thymol	Undecanoic acid & thymol	1 : 1 ^92^	✗	✗	Stable^92^	Heating and stirring^92^
Thymol : 10-undecylenic acid	10-Undecylenic acid & thymol	10-Undecylenic acid & thymol	(2.33 : 1, 1.5 : 1, 1 : 1, 1 : 2, 1 : 3, and 1 : 4),^37^ 1 : 1 ^103^	✓^37^^d^	✓ (density (25 °C), viscosity (25 °C), and melting point for 2.33 : 1, 1.5 : 1, 1 : 1, 1 : 2, 1 : 3, and 1 : 4 molar ratios)^37^	Stable^37,103^	Heating and stirring^37,103^
					✓ (density and viscosity for 1 : 1 molar ratio at different temperatures)^103^		
Thymol : dodecanoic acid (lauric acid)	Lauric acid & thymol	Lauric acid & thymol	2 : 1,^50^ 1 : 1,^57^ (1 : 1, 1 : 2, 1 : 3, 2 : 1, and 3 : 1),^59^ (the whole composition range was investigated, and the eutectic point was specified as 1.273 : 1 molar ratio (*x*^*E*^_thy_ = 0.56, *T*^*E*^ 24.56 °C)),^34^ 1.25 : 1,^52^ 1 : 1,^48^ (0.5 : 1, 1 : 1, 2 : 1, 3 : 1),^58^ 1.22 : 1,^36^ (1 : 1, 1 : 2, and 2 : 1),^75^ 1.273 : 1,^64^ 1 : 1,^69^ 1 : 1,^85^ (5 : 1, 2.5 : 1, 1.7 : 1, 1.25 : 1, 1 : 1, 1 : 1.2, 1 : 1.4, 1 : 1.6, 1 : 1.8, and 1 : 2)^88^	✓ ^34^	✓ Melting point for 1 : 1 molar ratio^59^	Stable^34,36,48,50,57,58,64,69,75,85^	Heating and stirring^34,36,48,50,52,57–59,64,69,75,85,88^
					✓ (density and viscosity for 1.222 : 1 molar ratio at different temperatures)^34^		
					✓ (density, viscosity, and surface tension for 1 : 1 molar ratio at different temperatures)^48^		
					✓ (density at 45 °C and melting point)^58^		
					✓ (density and viscosity for 1.22 : 1 molar ratio at 60 °C, and melting point for this molar ratio)^36^		
					✓ (density and rheological characteristics for 1 : 1, 1 : 2, and 2 : 1 molar ratios)^75^		
					✓ (electrical conductivity (room temperature) and viscosity (4 °C) for 1 : 1 molar ratio)^85^		
Thymol : tetradecanoic acid (myristic acid)	Myristic acid & thymol	Myristic acid & thymol	1 : 1,^104^ (the whole composition range was investigated, and the eutectic point was specified as 2.030 : 1 molar ratio (*x*^E^_thy_ = 0.67, *T*^E^ = 33.15 °C)),^34^ 3 : 1,^36^ 1 : 1,^88^ 1 : 1 ^105^	✓ ^34^	✓ (density and viscosity for 3 : 1 molar ratio at different temperatures)^34^	Stable^34,36,104,105^	Heating and stirring^34,36,88,104,105^
					✓ (density and viscosity for 3 : 1 molar ratio at 60 °C, and melting point for this molar ratio)^36^		
Thymol : hexadecanoic acid (palmitic acid)	Palmitic acid & thymol	Palmitic acid & Thymol	(The whole composition range was investigated, and the eutectic point was specified as 3.167 : 1 molar ratio (*x*^E^_thy_ = 0.76, *T*^E^ = 39.14 °C)),^34^ 4 : 1,^36^ 1 : 1 ^88^	✓ ^34^	✓ (density and viscosity for 4 : 1 molar ratio at different temperatures)^34^	Stable^34,36^	Heating and stirring^34,36,88^
					✓ (density and viscosity for 4 : 1 molar ratio at 60 °C, and melting point for this molar ratio)^36^		
Thymol : octadecanoic acid (stearic acid)	Stearic acid & thymol	Stearic acid & thymol	1 : 1,^104^ (the whole composition range was investigated, and the eutectic point was specified as 5.250 : 1 molar ratio (*x*^E^_thy_ = 0.84, *T*^E^ = 43.54 °C)),^34^ 9 : 1,^36^ 1 : 1,^88^ 1 : 1 ^105^	✓ ^34^	✓ (density and viscosity for 9 : 1 molar ratio at different temperatures)^34^	Stable^34,36,104,105^	Heating and stirring^34,36,88,104,105^
					✓ (density and viscosity for 9 : 1 molar ratio at 60 °C, and melting point for this molar ratio)^36^		
Thymol : octadecenoic acid (oleic acid)	Oleic acid & thymol	Oleic acid & thymol	1 : 1,^104^ (1 : 1 and 1 : 5),^96^ (the whole composition range was investigated, and the eutectic point was specified as 3 : 1 molar ratio (*x*^E^_thy_ = 0.75, *T*^E^ = −4 °C),^123^ 1 : 1,^67^ 1 : 1 ^105^	✓ ^123^	✓ (density, thermal expansion coefficient, refractive index, surface tension, dynamic viscosity, ionic conductivity, and thermal conductivity for 1 : 1 and 1 : 5 molar ratios at 25 °C)^96^	Stable^67,96,104,105,123^	Heating and stirring^67,96,104,105,123^
Thymol : octadecadienoic acid (linoleic acid)	Linoleic acid & thymol	Linoleic acid & thymol	(1 : 1 and 1 : 5)^96^	✗	✓ (density, thermal expansion coefficient, refractive index, surface tension, dynamic viscosity, ionic conductivity, and thermal conductivity for 1 : 1 and 1 : 5 molar ratios at 25 °C)^96^	Stable^96^	Heating and stirring^96^
Thymol : 2-hexyldecanoic acid	2-Hexyldecanoic acid & thymol	2-Hexyldecanoic acid & thymol	(1 : 1, 1 : 2, 1 : 3, and 1 : 4)^126^	✗	✓ (density, viscosity, and water content for 1 : 1, 1 : 2, 1 : 3, and 1 : 4 molar ratios at 25 °C)^126^	Stable^126^	Heating and stirring^126^
Thymo : phenylacetic acid	Phenylacetic acid & thymol	Phenylacetic acid & thymol	The whole composition range was investigated, and the eutectic point was specified as 3 : 1 molar ratio (*x*^E^_thy_ = 0.75, *T*^E^ = 3 °C)^128^	✓ ^128^	✓ (density, molar volume, and water content for 3 : 1 molar ratio at 25 °C)^128^	Stable^128^	Heating and stirring^128^
Thymol : citric acid	Citric acid	thymol	1 : 1,^129^ 1 : 1 ^130^^j^	✗	✓ (specific weight and polarity for 1 : 1 molar ratio)^130^	Stable^129^	Heating and stirring^129,130^
Thymol : polyacrylic acid	Polyacrylic acid	thymol	2 : 1 ^133^	✗	✗	Stable^133^	Heating and stirring^133^

*Thymol : amides/amines/nitrogen-containing heterocycle*							
Thymol : urea	Thymol	Urea	The whole composition range was investigated, and the eutectic point was specified as 5 : 1 molar ratio (*x*^E^_thy_ = 0.83, *T*^E^ = −5 °C),^128^ (1 : 1, 1 : 2, and 3 : 1)^129^	✓ ^128^	✓ (density, molar volume, and water content for 5 : 1 molar ratio at 25 °C)^128^	Stable^128,129^	Heating and stirring^128,129^
Thymol : monoethanolamine	Thymol & monoethanolamine	Thymol & monoethanolamine	2 : 1, 1 : 1, 1 : 2, 1 : 3, and 1 : 42 ^145^	✗	✓ (glass transition and water content for 1 : 3 molar ratio)^145^	—	Heating and stirring^145^
Thymol : *N*-methylethanolamine	Thymol & *N*-methylethanolamine	Thymol & *N*-methylethanolamine	1 : 1 ^145^	✗	✗	—	Heating and stirring^145^
Thymol : acetamide	Thymol	Acetamide	The whole composition range was investigated, and the eutectic point was specified in the range of (1.5–2.5):1 molar ratios (*x*^E^_thy_ = 0.60–0.71, *T*^E^ = −61.95 °C)^135^	✓ ^135^	✓ (density, sound velocity, viscosity, and refractive index for 2 : 1 molar ratio at different temperatures)^135^	Stable^135^	Heating and stirring^135^
Thymol : acetanilide	Thymol	Acetanilide	The whole composition range was investigated, and the eutectic point was specified in the range of (2–2.5):1 molar ratios (*x*^E^_thy_ = 0.67–0.71, *T*^E^ = −57.85 °C)^135^	✓ ^135^	✓ (density, sound velocity, viscosity, and refractive index for 2 : 1 molar ratio at different temperatures)^135^	Stable^135^	Heating and stirring^135^
Thymol : triethylenetetramine hydrochloride	Thymol	Triethylenetetramine HCL	3 : 1,^143^ (3 : 1 and 5 : 1)^144^	✗	✓ (density and viscosity for 3 : 1 molar ratio)^143^	Stable^143^	Heating and stirring ^143,144^
					✓ (density, viscosity, and water content for 3 : 1 and 5 : 1 molar ratios at 50 °C)^144^		
Thymol : tetraethylpentamine hydrochloride	Thymol	Tetraethylpentamine HCl	(3 : 1 and 5 : 1)^144^	✗	✓ (density, viscosity, and water content for 3 : 1 and 5 : 1 molar ratios at 50 °C)^144^	Stable^144^	Heating and stirring^144^
Thymol : 4-(dimethylamino)benzaldehyde	Thymol	4-(Dimethylamino)benzaldehyde	1 : 1 ^134^	✗	✓ (density (25 °C) and viscosity (30 °C) for 1 : 1 molar ratio)^134^	Stable^134^	Heating and stirring^134^
Thymol : 1,10-phenanthroline	Thymol	1,10-Phenanthroline	(The whole composition range was investigated, and the eutectic point was specified in the range of (2.33–4):1 molar ratios (*x*^E^_thy_ = 0.7–0.8),^136^ 2 : 1 ^137^	✓ ^136^	✓ (density and viscosity for 4 : 1 molar ratio at different temperatures)^136^	Stable^136,137^	Heating and stirring^136,137^
Thymol : lidocaine	Thymol	Lidocaine	(1 : 1 and 2 : 1),^138^ 1 : 1,^108^ 2 : 1,^139^ (2 : 1, 1 : 1, and 1 : 2),^140^ (the whole composition range was investigated, and the eutectic point was specified as about 1 : 1 molar ratio (*x*^E^_thy_ = 0.5)),^141^ 2 : 1 ^142^	✓ ^141^	✓ (density (25 °C), viscosity (25 °C), and water content (22 °C) for 1 : 1 and 2 : 1 molar ratios)^138^	Stable^108,138–141^	Heating and stirring,^108,138,141^ addition of both compounds to a container (without heating, stirring or another external factor)^139,140^, rotary evaporation at 20 °C (without solvent)^142^
					✓ (density (25 °C), viscosity (25 °C), and decomposition temperature for 2 : 1, 1 : 1, and 1 : 2 molar ratios)^141^		
					✓ (density, viscosity, and water content for 2 : 1 molar ratio at 30 °C)^142^		
Thymol : atropine	Atropine	Thymol	2 : 1 ^138^	✗	✓ (density (25 °C), viscosity (25 °C), and water content (22 °C) for 2 : 1 molar ratio)^138^	Stable^138^	Heating and stirring^138^
Thymol : caffeine	Thymol	Caffeine	3 : 1 ^127^	✗	✗	—	Heating and stirring^127^
Thymol : 1-(*o*-tolyl)thiourea	Thymol	1-(*o*-Tolyl)thiourea	The whole composition range was investigated, and the eutectic point was specified as 9 : 1 molar ratio (*x*^E^_thy_ = 0.9)^146^^k^	✓ ^146^	✓ (density and viscosity for 9 : 1 molar ratio at 25 °C)^146^	Stable^146^	Heating and stirring^146^
Thymol : *N*,*N*-dimethylformamide	Thymol	*N*,*N*-Dimethylformamide	1 : 1 147	✗	✗	Stable^147^	Heating and stirring^147^

*Thymol : alcohol/diol*							
Thymol : ethylene glycol	Thymol & ethylene glycol	Thymol & ethylene glycol	(1 : 3, 1 : 2, 1 : 1, 2 : 1, and 3 : 1),^148^ 1 : 2,^133^ 1 : 1 ^149^	✗	✓ (melting point for 2 : 1 molar ratio)^148^	Stable^133,148^	Heating and stirring^133,148,149^
Thymol : hexafluoroisopropanol	Hexafluoroisopropanol	Thymol	(1 : 2, 1 : 3, and 1 : 4)^153^	✗	✓ (density (40 °C), viscosity (25 °C), and melting point for 1 : 2, 1 : 3, and 1 : 4 molar ratios)^153^	Stable^153^	Heating and stirring^153^
Thymol : isobutanol	Thymol & isobutanol	Thymol & isobutanol	(1 : 1, 1 : 2, 2 : 1, and 3 : 1)^43^	✗	✗	Stable^43^	Heating and stirring^43^
Thymol : 1,4-butanediol	Thymol & 1,4-butanediol	Thymol & 1,4-butanediol	1 : 1 ^71^	✗	✗	Stable^71^	Heating and stirring^71^
Thymol : octanol	Thymol & octanol	Thymol & octanol	1 : 2,^55^ 1 : 1,^154^ 1 : 2,^124^ (2 : 1, 1 : 1, 1 : 2, and 1 : 3)^90^	✗	✓ (density (25 °C), surface tension (25 °C), and viscosity (50 °C) for 1 : 2 molar ratio) ^124^	Stable^55,90,124,154^	Heating and stirring^55,90,124,154^
Thymol : decanol	Thymol & decanol	Thymol & decanol	1 : 1.5,^55^ (1 : 2, 1 : 1, 2 : 1, 3 : 1, 4 : 1, and 5 : 1),^147^ 1 : 1 ^122^	✗	✗	Stable^55,147^	Heating and stirring^55,122,147^
Thymol : 1,2-decanediol	Thymol & 1,2-decanediol	Thymol & 1,2-decanediol	2 : 1 ^138^	✗	✓ (density (25 °C), viscosity (25 °C), and water content (22 °C) for 2 : 1 molar ratio)	Stable^138^	Heating and stirring^138^
Thymol : 1-dodecanol (lauryl alcohol)	Thymol & 1-dodecanol	Thymol & 1-dodecanol	1 : 1,^44^ (1 : 1 and 1 : 2) ^157^	✗	✗	Stable^44,157^	Heating and stirring^44,157^
Thymol : tetradecanol (myristyl alcohol)	Thymol	Tetradecanol	The whole composition range was investigated, and the eutectic point was specified as 1.2 : 1 molar ratio (*x*^E^_thy_ = 0.54, *T*^E^ ≈ 25 °C),^150^ 2 : 1,^151^ (1 : 2, 1 : 1, 2 : 1, and 3 : 1) ^152^^p^	✓ ^150^	✗	Stable^151,152^	Heating and stirring^150–152^
Thymol : hexadecanol (cetyl alcohol)	Thymol	Hexadecanol	The whole composition range was investigated, and the eutectic point was specified as 2.2 : 1 molar ratio (*x*^E^_thy_ = 0.69, *T*^E^ ≈ 35 °C),^150^ 1 : 1 ^130^^j^	✓ ^150^	✓ (specific weight and polarity for 1 : 1 molar ratio)^130^	—	Heating and stirring^130,150^
Thymol : octadecanol	Thymol	Octadecanol	The whole composition range was investigated, and the eutectic point was specified as 3.4 : 1 molar ratio (*x*^E^_thy_ ≈ 0.77, *T*^E^ ≈ 38 °C)^150^	✓ ^150^	✗	—	Heating and stirring^150^
Thymol : benzyl alcohol	Thymol & benzyl alcohol	Thymol & benzyl alcohol	(1 : 1 and 2 : 1),^43^ 1 : 4,^55^ 1 : 1,^154^ 2 : 1,^124^ 1 : 1 ^155^	✗	✓ (density (25 °C), surface tension (25 °C), and viscosity (50 °C) for 2 : 1 molar ratio)^124^	Stable^43,55,124,154^	Heating and stirring^43,55,124,154,155^
Thymol : 2-hydroxyethyl methacrylate	Thymol & 2-hydroxyethyl methacrylate	Thymol & 2-hydroxyethyl methacrylate	1 : 1 ^156^	✗	✗	Stable^156^^e^	Heating and stirring^156^
Thymol : polyethylene glycol 400 (PEG400)	Thymol & PEG400	Thymol & PEG400	1 : 1 43	✗	✗	Unstable^43^^f^	Heating and stirring^43^
Thymol : polyethylene glycol 200 (PEG200)	Thymol & PEG200	Thymol & PEG200	(1 : 2 and 2 : 1)^133^	✗	✗	Stable^133^	Heating and stirring^133^

*Thymol : terpenoid (oxygenated derivative of terpenes)*							
Thymol : borneol	Thymol	Borneol	(The whole composition range was investigated, and the eutectic point was specified as about a 1 : 1 molar ratio (*x*^E^_thy_ ≈ 0.5, *T*^E^ ≈ 10.55 °C)),^159^ (the whole composition range was investigated, and the eutectic point was specified as 1.4 : 1 molar ratio (*x*^E^_thy_ = 0.583, *T*^E^ = 20.05 °C)),^160^ (1 : 1, 2 : 1, 3 : 1, and 4 : 1),^158^ (1 : 1 and 2 : 1),^161^ (1 : 1, 1 : 2, and 2 : 1),^162^ 1 : 1,^111^ 2 : 1 ^163^^o^	✓ ^159^	✓ (density and viscosity for 1 : 1 molar ratio at different temperatures, and water content of this molar ratio before and after saturation with water at 22 °C)^159^	Stable^158,159,161,162^	Heating and stirring^111,158–162^, heating without stirring^163^
				✓ ^160^	✓ (density, viscosity, surface tension, and refractive index for 2.33 : 1 molar ratio at 25 °C)^160^		
					✓ (density, viscosity, and water content before and after saturation with water, for 1 : 1, 2 : 1, 3 : 1, and 4 : 1 molar ratios at 25 °C)^158^		
Thymol : fenchyl alcohol (fenchol)	Thymol	Fenchol	The whole composition range was investigated, and the eutectic point w as specified as about a 1 : 1.5 molar ratio (*x*^E^_thy_ = 0.4)^164^	✓ ^164^	✓ (density (25 °C) and viscosity (different temperatures) for the eutectic point)^164^	Stable^164^	Heating and stirring^164^
Thymol : terpineol	Thymol	Terpineol	1 : 1.3,^164^ (1 : 1, 2 : 1, and 3 : 1),^161^ (1 : 1, 1 : 2, and 2 : 1)^162^	✗	✓ (density (25 °C) and viscosity (different temperatures) for the eutectic point)^164^	Stable^161,162,164^	Heating and stirring^161,162,164^
Thymol : β-citronellol	Thymol	β-Citronellol	1 : 1 ^181^	✗	✓ (density (25 °C) and viscosity (different temperatures) for 1 : 1 molar ratio)^181^	—	Heating and stirring^181^
Thymol : menthol	Thymol	Menthol	1 : 1,^26^ (the whole composition range was investigated, and the eutectic point was specified as about a 1 : 1 molar ratio (0.45 ≤ *x*^E^_thy_ ≤ 0.50, *T*^E^ = −50 °C,^38^ (1 : 1 and 1 : 2),^45^ 1 : 1,^187^ 2 : 1,^188^ 1 : 1,^189^ 1 : 1,^190^ 1 : 1,^191^ (1.04 : 1 and 1 : 1.44),^169^ (1 : 2 and 1 : 4),^192^ (1 : 1 and 1 : 2),^54^ (the whole composition range was investigated, and the eutectic point was specified as 1 : 2.33 molar ratio (*x*^E^_thy_ = 0.3, *T*^E^ = 17 °C)),^154^ g (2 : 1, 1 : 1, and 1 : 2),^193^ (the whole composition range was investigated, and the eutectic point was specified as 1 : 1.15 molar ratio (*x*^E^_thy_ = 0.4651, *T*^E^ = −59.39 °C)),^182^ (1 : 1, 2 : 1, 3 : 1, 4 : 1, and 1 : 2),^194^ (1.5 : 1, 1 : 1, and 1 : 2),^195^1 : 1,^159^ 1 : 1,^55^ 1 : 1,^113^ (1 : 2, 1 : 1, and 2 : 1),^171^ (1 : 1 and 1 : 2),^138^ (1 : 2.5, 1 : 2, 1 : 1.5, 1 : 1, 1.5 : 1, 2 : 1, and 2.5 : 1),^106^ 1 : 3,^100^ (the whole composition range was investigated, and the eutectic point was specified as about a 1 : 1.2 molar ratio (*x*^E^_thy_ = 0.46, *T*^E^ = −61 °C)),^196^ 1 : 1,^166^ 1 : 1,^43^ 1 : 1,^117^ 1 : 1.1,^164^ (1 : 1, 1 : 2, and 2 : 1),^65^ (1 : 1, 1 : 2, 1 : 3, 1 : 4, 1 : 5, 2 : 1, 3 : 1, 4 : 1, and 5 : 1),^167^ (1 : 1, 1 : 2, and 2 : 1),^183^ (different molar ratios from 1 : 3.5 to 3.5 : 1),^184^ (the whole composition range was investigated, and the eutectic point was specified as about a 1 : 1.22 molar ratio (*x*^E^_thy_ = 0.45, *T*^E^ = −73 °C)),^185^ (1 : 4.9, 1 : 4, 1 : 3, 1 : 2, 1 : 1, 2 : 1, 3 : 1, 4 : 1, and 4.9 : 1),^51^ (1 : 1),^186^ (1 : 1, 2 : 1, and 1 : 2),^66^ 2 : 1,^197^ 1 : 1,^103^ 1 : 1,^118^ 2 : 1,^124^ 1 : 1,^131^ 5 : 1,^147^ 1 : 1,^101^ (1 : 1, 1 : 2, 2 : 1, and 4 : 1),^69^ (1 : 1, 1 : 2, and 2 : 1),^198^ 1 : 1,^199^ 1.5 : 1,^200^ (1 : 1 and 2 : 1),^161^ (1 : 1, 1 : 2, and 2 : 1),^162^ (1 : 1 and 2 : 1),^71^ 1 : 1,^201^ 1 : 1,^120^ (4 : 1, 2.3 : 1, 1.5 : 1, 1 : 1, 1 : 1.5, 1 : 2.3, 1 : 4, and 1 : 9),^202^ (1 : 3, 1 : 2, 1 : 1, 2 : 1, and 3 : 1),^203^ 1 : 1.5,^204^ 1 : 1,^175^ 1 : 1,^108^ 1 : 1,^74^ 1 : 1,^82^ 1 : 1,^85^ 1 : 1,^122^ (2 : 1, 1 : 1, and 1 : 2),^205^ (1 : 1 and 1 : 1.5),^163^ (1 : 1, 2 : 1, and 5 : 1),^206^ 1 : 1,^207^ (2 : 1, 1 : 1, and 1 : 2),^208^ (1 : 1, 2 : 1, 3 : 1, and 4 : 1),^209^ 1 : 1,^112^ (1 : 4, 1 : 3, 1 : 2, 1 : 1, 2 : 1, and 3 : 1),^152^ p 1 : 1,^210^ (1 : 1 and 1 : 2),**211** 1 : 1.5 ^91^	✓ ^38^	✓ (density and polarity for 1 : 1 molar ratio at 25 °C)^26^	Stable^26,43,45,51,55,65,66,69,71,74,85,100,103,108,112,113,117,118,120,124,131,138,147,152,154,159,161,162,164,171,182,184–186,188,193,194,198–201,203,205,208,209^	Heating and stirring,^38,43,45,51,54,55,65,66,69,71,74,82,85,91,100,101,103,106,108,112,113,117,118,120,122,124,131,138,147,152,154,159,162,164,166,167,169,171,175,182–188,190–198,200–211^ heating, stirring, and sonication,^208^ stirring at room temperature,^199^ heating without stirring,^163^*in situ* formation^26^
				✓ ^154^^g^	✓ (vapor–liquid Equilibrium behavior for the full composition range)^38^		
				✓ ^182^	✓ (glass transition and water content (before and after saturation with water) for 1 : 1 and 2 : 1 molar ratios)^45^		
				✓ ^196^	✓ (density and surface tension at 25 °C and 20 °C, respectively)^169^		
				✓ ^185^	✓ (polarity and water content at room temperature, density and viscosity at different temperatures, for 1 : 2 and 1 : 4 molar ratios)^192^		
					✓ (glass transition for 2 : 1, 1 : 1, and 1 : 2 molar ratios)^193^		
					✓ (density, speed of sound, isobaric molar heat capacity, refraction index, static permittivity, surface tension, and viscosity for 2 : 1, 1 : 1, and 1 : 2 molar ratios at different temperatures)^182^		
					✓ (density and viscosity for 1 : 1 molar ratio at different temperatures, and water content of this molar ratio before and after saturation with water at 22 °C)^159^		
					✓ (density (25 °C), viscosity (25 °C), and water content (22 °C), for 1 : 1 and 1 : 2 molar ratios)^138^		
					✓ (density, viscosity, thermal conductivity, and refractive index for 1 : 1 molar ratio at different temperatures, and water content at room temperature)^106^		
					✓density for 1 : 3 molar ratio^100^		
					✓ (density (25 °C) and melting point for 1 : 1 molar ratio)^166^		
					✓ (density (25 °C) and viscosity (different temperatures) for the eutectic point)^164^		
					✓ (pH for 1 : 1, 1 : 2, 1 : 3, 1 : 4, 1 : 5, 2 : 1, 3 : 1, 4 : 1, and 5 : 1 molar ratios and viscosity for 1 : 1 and 1 : 5 molar ratios, at room temperatures)^167^		
					✓ (density (different temperatures), viscosity (different temperatures), water miscibility (25 °C), and surface tension (25 °C), for 1 : 4.9, 1 : 4, 1 : 3, 1 : 2, 1 : 1, 2 : 1, 3 : 1, 4 : 1, and 4.9 : 1 molar ratios)^51^		
					✓ (density (different temperatures) and water content (room temperature) for 2 : 1 molar ratio)^197^		
					✓ (density and viscosity for 1 : 1 molar ratio at different temperatures)^103^		
					✓ (density (25 °C), surface tension (25 °C), and viscosity (50 °C) for 2 : 1 molar ratio)^124^		
					✓ (density, viscosity, and surface tension for 1 : 1 molar ratio at 25 °C)^101^		
					✓ (density (25–80 °C), viscosity (25–80 °C), and glass transition for 1 : 1 molar ratio)^201^		
					✓ (density and water content for 9 : 1^m^, 4 : 1, and 2.3 : 1 molar ratios at 25 °C, viscosity for 4 : 1 and 2.3 : 1 molar ratios at 15 °C– 55 °C, and melting point for 9 : 1 to 1 : 9 molar ratios)^202^		
					✓ (melting point of 1 : 3, 1 : 2, 1 : 1, 2 : 1, and 3 : 1 molar ratios)^203^		
					✓ (decomposition temperature for 1 : 1 molar ratio)^175^		
					✓ (density and viscosity for 1 : 1 molar ratio at 20 °C)^82^		
					✓ (electrical conductivity (room temperature) and viscosity (4 °C) for 1 : 1 molar ratio)^85^		
					✓ (melting point and decomposition temperature for 2 : 1, 1 : 1, and 1 : 2 molar ratios)^205^		
					✓ (density for 5 : 1 molar ratio at room temperature)^206^		
					✓ (density, viscosity, electical conductivity, and pH for 1 : 1 and 1 : 2 molar ratios at different temperatures)^211^		
					✓ (polarity, density, and viscosity for 1 : 1.5 molar ratio at 25 °C)^91^		
Thymol : sobrerol	Thymol & sobrerol	Thymol & sobrerol	(the whole composition range was investigated, and the eutectic point was specified as about a 2.33 : 1 molar ratio (*x*^E^_thy_ = 0.7, *T*^E^ = 27 °C)) ^159^	✓ ^159^	✗	Stable^159^	Heating and stirring^159^
Thymol : camphor	Thymol	Camphor	1 : 1,^26^ (1 : 1.48, 1.01 : 1, and 1.52 : 1),^169^ (3.04 : 1, 2.03 : 1, 1.01 : 1, 1 : 2.96, and 1 : 1.97),^47^ 1 : 1, ^53^ (the whole composition range was investigated, and the eutectic point was specified as 1 : 1.5 molar ratio (*x*^E^_thy_ = 0.4, *T*^E^ = 16 °C)),^154^^g^ (the whole composition range was investigated, and the eutectic point was specified as about a 1 : 1 molar ratio (*x*^E^_thy_ = 0.5, *T*^E^ = −73 °C)), 1 : 1 ^113^ (1 : 2, 1 : 1, and 2 : 1),^171^ 1 : 1,^166^ (the whole composition range was investigated, and the eutectic point was specified as 1.2 : 1 molar ratio (*x*^E^_thy_ = 0.547, *T*^E^ = −35.25 °C)),^160^ 1 : 1.5,^164^ (1 : 1, 1 : 2, 1 : 3, 1 : 4, 1 : 5, 2 : 1, 3 : 1, 4 : 1, and 5 : 1),^167^ (1 : 1, 1 : 2, and 2 : 1),^65^ 1 : 1,^53^ (1 : 1, 2 : 1, 2.5 : 1, 3 : 1, 4 : 1, and 1 : 2),^165^ (1 : 1, 2 : 1, and 1 : 2),^66^ (2.33 : 1, 1.5 : 1, and 1 : 1),^37^ (1 : 1, 1 : 2, and 2 : 1),^168^ 1 : 1,^170^ 1 : 1,^172^ (1 : 1, 1 : 2, and 2 : 1),^174^ 1 : 1,^173^ (1 : 1, 1 : 2, and 2 : 1),^162^ 1 : 1,^175^ 1 : 1,^108^ 1 : 1,^80^ 1 : 1,^176^ (2.33 : 1, 1.5 : 1, 1 : 1, 1 : 1.5, and 1 : 2.33),^122^ 1 : 1,^177^ (1 : 1.5, 1 : 1, 1.14 : 1, 2 : 33 : 1, 3.33 : 1, and 4 : 1),^163^ 1 : 1 ^178^	✓ ^154^ g	✓ (density and polarity for 1 : 1 molar ratio at 25 °C)^26^	Stable^26,37,47,53,65,66,80,108,113,154,159,162,164,165,168,170–172,174,176^	Heating and stirring,^37,47,53,65,66,108,113,122,154,159,160,162,164–175,178^ stirring at room temperature ^80,176,177^, heating without stirring,^163^*in situ* formation^26^
				✓ ^159^	✓ (density and surface tension at 25 °C and 20 °C, respectively)^169^		
				✓ ^160^	✓ (density and viscosity for 1 : 1 molar ratio at different temperatures, and water content of this molar ratio before and after saturation with water at 22 °C)^159^		
				✓ ^37^ d	✓ (density (25 °C) and melting point for 1 : 1 molar ratio)^166^		
					✓ (density, viscosity, surface tension, and refractive index for 1 : 1 molar ratio at 25 °C)^160^		
					✓ (density (25 °C) and viscosity (different temperatures) for the eutectic point)^164^		
					✓ (pH for 1 : 1, 1 : 2, 1 : 3, 1 : 4, 1 : 5, 2 : 1, 3 : 1, 4 : 1, and 5 : 1 molar ratios and viscosity for 1 : 1 and 5 : 1 molar ratios, at room temperatures)^167^		
					✓ (melting point for 1 : 1 molar ratio)^66^		
					✓ (density and viscosity for 2.33 : 1, 1.5 : 1, and 1 : 1 molar ratios at 25 °C, and melting point for 2.33 : 1, 1.5 : 1, 1 : 1, 1 : 2, 1 : 3, and 1 : 4 molar ratios)^37^		
					✓ (decomposition temperature for 1 : 1 molar ratio)^175^		
					✓ (decomposition temperature for 1 : 1.5 molar ratio)^122^		
					✓ (viscosity for 1 : 1.5, 1.14 : 1, and 3.33 : 1 molar ratios at 22–40 °C)^163^		
					✓ (pressure – volume – temperature (PVT) measurements across 0.1–60 MPa and 20–70 °C)^178^		
Thymol : carvone	Thymol	Carvone	(2.5 : 1, 2 : 1, 1.5 : 1, 1 : 1, 1 : 2, and 1 : 2.5; the predicted eutectic point using the COSMO-RS method corresponds to 1 : 1 molar ratio (*x*^E^_thy_ = 0.5, *T*^E^ ≈ −118 °C)),^179^ 2 : 1,^101^ 1 : 1,^173^ 1 : 1,^180^ 1 : 1,^176^ 1 : 1 ^177^	✗	✓ (density (different temperatures), viscosity (different temperatures), refractive index (different temperatures), surface tension (20 °C), water content (25 °C), thermal conductivity (25 °C), and polarity (25 °C) for 1 : 1 molar ratio^179^	Stable^176^	Heating and stirring,^101,173,179,180^ stirring at room temperature^176,177^
					✓ (density, viscosity, and surface tension for 2 : 1 molar ratio at 25 °C)^101^		
Thymol : cineole	Thymol	Cineole	(2.5 : 1, 2 : 1, 1.5 : 1, 1 : 1, 1 : 2, and 1 : 2.5; the predicted eutectic point using the COSMO-RS method corresponds to 1 : 1 molar ratio (*x*^E^_thy_ = 0.5, *T*^E^ = −173 °C)),^179^ 1 : 1,^180^ 1 : 1 ^178^	✗	✓ (density (different temperatures), viscosity (different temperatures), refractive index (different temperatures), surface tension (20 °C), water content (25 °C), thermal conductivity (25 °C), and polarity (25 °C) for 1 : 1 molar ratio)^179^	—	Heating and stirring^**178–1**80^
					✓ (pressure – volume – temperature (PVT) measurements across 0.1–60 MPa and 20–70 °C)^178^		
Thymol : linalool	Thymol & linalool	Thymol & linalool	2 : 1 ^101^	✗	✓ (density, viscosity, and surface tension for 2 : 1 molar ratio at 25 °C)^101^	—	Heating and stirring^101^
Thymol : geraniol	Thymol & geraniol	Thymol & geraniol	1 : 1 ^101^	✗	✓ (density, viscosity, and surface tension for 1 : 1 molar ratio at 25 °C)^101^	—	Heating and stirring^101^
Thymol : carvacrol	Thymol & carvacrol	Thymol & carvacrol	1 : 1 ^212^	✗	✓ (melting point for 1 : 1 molar ratio)^212^	Stable^212^	Heating and stirring^212^

**Thymol** : **phenol and phenolic derivatives**							
Thymol : phenol	Thymol & phenol	Thymol & phenol	(1 : 1, 1 : 2, and 2 : 1)^232^	✗	(Density (ambient temperature), viscosity (25 °C), and glass transition for 1 : 1, 1 : 2, and 2 : 1 molar ratios. Melting point for 1 : 2 and 2 : 1 molar ratios)^232^	Stable^232^	Heating and stirring^232^
Thymol : 2-chlorophenol	Thymol & 2-chlorophenol	Thymol & 2-chlorophenol	(1 : 1, 1 : 2, and 2 : 1)^65^	✗	✗	Stable^65^	Heating and stirring^65^
Thymol : 2,6-xylenol	Thymol & 2,6-xylenol l	Thymol & 2,6-xylenol	1 : 1 ^197^	✗	✓ (density (different temperatures) and water content (room temperature) for 1 : 1 molar ratio)^197^	—	Heating and stirring^197^
Thymol : coumarin	Thymol	Coumarin	(2 : 1 and 1 : 1),^138^ 1.5 : 1,^196^ 1.2 : 1,^164^ (1 : 1 and 2 : 1),^113^ (1 : 2, 1 : 1, and 2 : 1),^171^ (1 : 1, 1 : 2, and 2 : 1),^168^ (1 : 1, 1 : 2, and 2 : 1),^170^ 1 : 1,^127^ 1 : 1,^172^ (1 : 1, 2 : 1, 3 : 1, 4 : 1, and 5 : 1),^235^ (1 : 1, 2 : 1, 1 : 2),^174^ 1 : 1,^103^ 2 : 1,^236^ 1 : 1,^108^ (2 : 1 and 1 : 1),^211^ 1.5 : 1 ^91^	✗	✓ (density (25 °C), viscosity (25 °C), and water content (22 °C) for 2 : 1 and 1 : 1 molar ratios)^138^	Stable^103,108,113,138,164,168,170–172,174,235,236^	Heating and stirring,^91,103,108,113,127,138,164,168,170–172,174,196,211,235^ heating and vortexing^236^
					✓ (density (25 °C) and viscosity (different temperatures) for the eutectic point)^164^		
					✓ (density and viscosity for 1 : 1 molar ratio at different temperatures)^103^		
					✓ (density, viscosity, electical conductivity, and pH for 2 : 1 and 1 : 1 molar ratios at different temperatures)^211^		
					✓ (polarity, density, and viscosity for 1.5 : 1 molar ratio at 25 °C)^91^		
Thymol : 6-methylcoumarin	Thymol	6-Methylcoumarin	(1 : 1, 2 : 1, and 3 : 1),^236^ 2.132 : 1,^247^ 1.066 : 1 ^248^	✗	✗	Stable^236,247,248^	Heating and vortexing^236,247,248^
Thymol : vanillin	Thymol & vanillin	Thymol & vanillin	1 : 1,^242^^k^ 1 : 1,^243^^i^ 1 : 1,^72^^i^ 1 : 1,^244^^i^ (1.5 : 1, 2.33 : 1, 4 : 1, and 9 : 1),^245^ 1 : 1,^246^ 1 : 1 ^111^	✗	✓ (density and viscosity for 1.5 : 1, 2.33 : 1, 4 : 1, and 9 : 1 molar ratios at 10–80 °C. Further, melting point, decomposition temperature and water content (30 °C) for 4 : 1 molar ratio)^245^	Stable^72,242–246^	Heating and stirring^72,242–246^
Thymol : 2,6-dimethoxyphenol (syringol)	Thymol & syringol	Thymol & syringol	(1 : 1, 1 : 2, and 2 : 1)^232,233^	✗	✓ (density (ambient temperature), viscosity (25 °C), and glass transition for 1 : 1, 1 : 2, and 2 : 1 molar ratios)^232^	Stable^232,233^	Heating and stirring^232,233^
Thymol : eugenol	Thymol & eugenol	Thymol & eugenol	1 : 1,^127^ 1 : 2,^234^ 1 : 1 ^175^	✗	✓ (decomposition temperature for 1 : 1 molar ratio)^175^	Stable^234^	Heating and stirring^127,175,234^
Thymol : phenyl salicylate (salol)	Salol	Thymol	1 : 1,^26^ (The whole composition range was investigated, and the eutectic point was specified as 1 : 1.5 molar ratio (*x*^E^_thy_ = 0.4, *T*^E^ = 28 °C))^154^^g^	✓ ^154^^g^	✓ (density and polarity for 1 : 1 molar ratio at 25 °C)^26^	Stable^26,154^	Heating and stirring,^154^*in situ* formation^26^
Thymol : flavone	Thymol	Flavone	(The whole composition range was investigated, and the eutectic point was specified slightly above 1.5 : 1 molar ratio (0.6 < *x*^E^_thy_ < 0.7, *T*^E^ = 13 °C))^241^	✓ ^241^	✗	—	Heating and stirring^241^
Thymol : flavanone	Thymol	Flavanone	(The whole composition range was investigated, and the eutectic point was specified slightly above 1.5 : 1 molar ratio (*x*^E^_thy_ ≈ 0.6, *T*^E^ = −5 °C)^241^	✓ ^241^	✗	—	Heating and stirring^241^
Thymol : benzophenone	Thymol	Benzophenone	(1 : 1, 1 : 2, and 2 : 1)^249^	✗	✗	Stable^249^	Ultrasonication without heating^249^

*Thymol : others*							
Thymol : water	Thymol	Water	(The whole composition range was investigated, and the eutectic point was specified as 4.8 : 1 molar ratio (*x*^E^_thy_ =0.83, *T*^E^ = −6 °C)),^128^ (5 : 1 and 10 : 1)^129^	✓ ^128^	✓ (density, molar volume, and water content for 4.8 : 1 molar ratio at 25 °C)^128^	Stable^128,129^	Heating and stirring,^128,129^*in situ* formation^128^
Thymol : trioctylphosphine oxide (TOPO)	Thymol	TOPO	1 : 1,^250^ (The whole composition range was investigated, and the eutectic point was specified as about 2.33 : 1 molar ratio (*x*^E^_thy_ ≈ 0.7, *T*^E^ = −13.15 °C)),^127^ 1 : 1 ^108^	✓ ^127^	✓ (refractive index for 1 : 1 molar ratio at different temperatures)^250^	Stable^108,127,250^	Heating and stirring^108,127,250^
					✓ (density and viscosity for 1 : 1 molar ratio at different temperatures, and water content for this molar ratio before and after saturation with water at 25 °C)^127^		
Thymol : triisobutylphosphine sulfide	Thymol	Triisobutylphosphine sulfide	(The whole composition range was investigated, and the eutectic point was specified as about 1 : 1-1 : 1.5 molar ratio (0.4 *x*^E^_thy_ ≤ 0.5))^251^	✓ ^251^	✓ (density, viscosity, and refractive index for 1 : 1 molar ratio in the temperature renge of 15–65 °C)^251^	Stable^251^muhulakshmi	Heating and stirring^251^
Thymol : bis(2-ethylhexyl) phosphoric acid	Thymol	Bis(2-ethylhexyl) phosphoric acid	(1 : 1, 2 : 1, and 3 : 1)^252^	✗	✓ (density (25 °C), viscosity (25 °C), and melting point for 2 : 1 molar ratio)^252^	Stable^252^	Heating and stirring^252^
Thymol : 3-hydroxyhexanoate	Thymol & 3-hydroxyhexanoate	Thymol & 3-hydroxyhexanoate	1 : 1 ^253^	✗	✗	Stable^253^	*In situ* formation^253^
Thymol : butyl lactate	Thymol & butyl lactate	Thymol & butyl lactate	1 : 1 ^253^	✗	✗	Stable^253^	*In situ* formation^253^
Thymol : methyl salicylate	Thymol & methyl salicylate	Thymol & methyl salicylate	1 : 1 ^253^	✗	✗	Stable^253^	*In situ* formation^253^
Thymol : poly(methacrylic acid-*co*-ethylene glycol dimethacrylate)	Poly(methacrylic acid-co-ethylene glycol dimethacrylate)	Thymol	1 : 1 ^132^	✗	✗	Stable^132^	Heating and stirring^132^
Thymol : 4-methoxybenzaldehyde	Thymol	4-Methoxybenzaldehyde	(5 : 1, 4 : 1, 3 : 1, 2 : 1, 1 : 1, 1 : 2, 1 : 3, 1 : 4, and 1 : 5)^246^	✗	✓ (density and viscosity for 1 : 1 molar ratio at 20 °C, and melting point for 2 : 1, 1 : 1 1 : 2, and 1 : 3 molar ratios)^246^	Stable^246^	Heating and stirring^246^
Thymol : 2,4,5-trimethoxybenzaldehyde	Thymol	2,4,5-Trimethoxybenzaldehyde	1 : 1 ^246^	✗	✗	Stable^246^	Heating and stirring^246^
Thymol : 4-methylbenzaldehyde	Thymol	4-Methylbenzaldehyde	1 : 1 ^246^	✗	✗	Stable^246^	Heating and stirring^246^

a The molar ratio 1 : 5 of thymol : acetic acid was examined but it did not lead to a transparent liquid (it resulted in solidification).^96^

b Thymol : acetic acid at different molar ratios (1 : 1, 1 : 2, 1 : 3, 1 : 4, and 2 : 1) as hydrophobic NDESs were used for dispersive liquid–liquid microextraction (DLLME). Although the paper does not provide quantitative data on the stability of thymol : acetic acid DES in water, its successful application in a DLLME procedure indicates that this DES maintains a degree of stability and forms a separate hydrophobic phase.^44^

c The 1 : 1 and 5 : 1 molar ratios of thymol : acetic acid DES showed instability in water (precipitation), while the 1 : 10 molar ratio was hydrophilic. In summary, the trend observed was that increasing the proportion of acetic acid relative to thymol enhances the water compatibility of the DES. The 1 : 10 molar ratio (thymol : acetic acid) resulted in a hydrophilic DES. Conversely, the 1 : 1 and 5 : 1 ratios led to precipitation, signifying instability in aqueous environment.^96^

d In the ref. 37 and 76, the deviation of the experimental solid–liquid equilibrium phase diagram from the ideal state has not been investigated. Therefore, it cannot be commented whether thymol : 10-undecylenic acid eutectic solvent is a deep eutectic or not.

e In this ref. 156, the water stability of cross-linked copolymeric [(2-hydroxyethyl methacrylate)_*m*_-*co*-(ethylene glycol dimethacrylate)_*n*_] : [thymol] DES, which is a eutectogel (deep eutectic solvent gel), has been investigated. There is no experimental evidence regarding the behavior of the DES (thymol : 2-hydroxyethyl methacrylate) in water.

f Thymol : PEG400 DES has not shown stability in water. The interactions with water molecules separate the DES components, causing PEG400 to go to the bottom of the tube and thymol to crystallize on the surface of the aqueous phase^43^ However, according to the ref. 133, thymol : PEG200 DES has good water stability and has successfully been used in the liquid–liquid microextraction procedure.

g According to the SLE data obtained in this ref. 154, the experimental eutectic points of thymol : salol, thymol : camphor, and thymol : menthol are higher than the ideal eutectic points. Therefore, based on the definition provided by Cherniakova *et al.*^32^ regarding eutectic solvents, these binary systems (thymol : salol, thymol : camphor, and thymol : menthol) are shallow eutectic solvents, although the ref. 154 claims that these systems are DES, according to the depression of the mixtures melting temperature compared to the melting points of starting components However, the SLE data obtained for thymol : camphor and thymol : menthol in the other literature ^38,159,160,182,185,196^ showed remarkable negative deviations from ideality, echoing that the eutectic solvent is deep.

h According to ref. 220, the water stability of thymol : choline chloride eutectic mixtures increases with increasing the mole ratio of thymol to choline chloride, so that thymol : choline chloride mixture in 1 : 1 and 2 : 1 molar ratios is water-soluble, in 3 : 1 molar ratio slightly forms a separate layer with water, and in 4 : 1 molar ratio could easily form a separate layer with water. The ref. 102 points out that thymol : choline chloride mixture in 2 : 1 molar ratio precipitate a solid white at the bottom of the tube, showing no stability when added to the water sample. However, in the other ref. 221, 222 and 225, a thymol : choline chloride mixture with a molar ratio of 2 : 1 was used in a liquid–liquid extraction process. Namely, according to ref. 221, 222 and 225, the 2 : 1 molar ratio of thymol : choline chloride mixture has shown water-immiscibility and separation from the aqueous phase.

i Although thymol : vanillin (1 : 1) has been reported as a DES in several ref. 72, 111, 242–244 and 246, the ref. 232 and 245 have not considered thymol : vanillin (1 : 1) as a stable eutectic mixture. According to ref. 232, a 1 : 1 molar ratio of thymol : vanillin forms a liquid upon heating and retains this liquid form after being kept at room temperature for 24 hours. However, this mixture (thymol : vanillin, 1 : 1) is not stable, as it crystallizes within one week, despite appearing liquid for the first several days.^232^ Also, the ref. 245 has mentioned that 1 : 1 molar ratio of thymol : vanillin is metastable and exhibits quick crystallization at temperatures below 25 °C. However, according to the ref. 244, thymol : vanillin mixtures at the molar ratios of 1 : 2 and 2 : 1 solidify after cooling at room temperature, while the solvent with the molar ratio of 1 : 1 remains as a transparent yellow liquid at ambient conditions for at least one week.

j The ref. 130 calls thymol : citric acid and thymol : cetyl alcohol Solid Eutectic Systems (SES), and defines SES as a homogeneous solid system at room temperature.

k The ref. 146 has not investigated the deviation of the experimental solid–liquid equilibrium phase diagram of thymol + 1-(*o*-tolyl)thiourea binary system from the ideal state. Therefore, it cannot be confirmed whether thymol : 1-(*o*-tolyl)thiourea (9 : 1) eutectic mixture is a deep eutectic or not.

l The water-stability of thymol : TBACl DES increase with increasing the molar ratio of thymol to the salt. The 2 : 1 molar ratio of thymol : TBACl DES is unstable in water (leaching of the salt clearly appreciable on the aqueous phase ^1^H NMR spectrum), but 3 : 1 and 4 : 1 molar ratio of thymol : TBACl DES is water-stable.^70^

m The thymol:menthol mixture in the molar ratio of 9 : 1 is not stable liquid, and turns to a gel-like solid after overnight monitoring at room temperature.^202^

n The deviation of the experimental solid–liquid equilibrium phase diagram of thymol : Aliquat 336 from the ideal state has not been investigated in the ref. 231.

o The ref. 163 also investigated the molar ratio of 3 : 1 of thymol : borneol, however, this mixture turned out to be unstable and crystallized very quickly.^163^

p The binary mixtures of thymol : menthol and thymol : 1-tetradecanol at a 4 : 1 molar ratio showed re-crystallization after cooling the transparent liquid to room temperature.^152^

Table 2 also shows that SLE phase diagram is available for which thymol-based binary eutectic mixtures. In cases where a source has determined the SLE phase diagram for a eutectic mixture (indicated by a checkmark in the SLE column), a separate indication of the melting point determination was deemed unnecessary in the physicochemical properties column. This is because the determination of the SLE phase diagram implicitly includes the melting point data. A mixture can be definitively classified as a DES only in cases where the “SLE phase diagram” column is checked. In instances where the SLE behavior has not been investigated, the system may not exhibit the necessary negative deviation from ideality, and it could merely be a eutectic liquid rather than a deep eutectic one. For example, Tiecco *et al.*^128^ found that although the thymol : benzoic acid mixture (4 : 1 molar ratio) is a liquid at room temperature and exhibits a melting point depression compared to the pure components (the melting points of thymol and benzoic acid are 49 °C and 121 °C, respectively), it cannot be classified as a DES because the SLE curve of this system didn't show a meaningful shift from the theoretical ideal curve, and activity coefficients deviated only negligibly from unity.

The physicochemical properties of thymol-based DESs are strongly influenced by the molecular structure of thymol and the nature of its counterpart component. In thymol, the phenolic hydroxyl group and the aromatic ring (along with the methyl and isopropyl groups, which are hydrophobic alkyl substituents) play key roles in hydrogen bonding and the molecule's hydrophobicity, respectively. The structure and function of the HBD/HBA partner determine the strength and organization of the intermolecular interaction network, thereby influencing the reduction in melting point, viscosity, polarity, water miscibility, and extraction efficiency. For example, the addition of carboxylic acids generally enhances hydrogen-bonding interactions *via* carbonyl and hydroxyl functional groups, leading to the formation of stable hydrophobic DESs with high affinity for organic analytes, whereas increasing the alkyl chain length of fatty acids can raise hydrophobicity and viscosity due to enhanced van der Waals interactions.^257,258^ Similarly, DESs containing alcohols, amides, or nitrogen-containing compounds exhibit distinct physicochemical behaviors, depending on their hydrogen-bonding capability and molecular flexibility.^91^ Therefore, the judicious selection of DES components and their molar ratios offers an effective strategy for tuning the structure, and consequently optimizing the properties, of thymol-based DESs for specific applications.^259^

Thymol plays a versatile role in the formation of DESs when combined with various substances, acting as either a HBD or HBA depending on the partner molecule. In combinations with menthol, camphor, coumarin, and borneol, thymol's hydroxyl group (–OH) typically serves as an HBD, interacting with the lone pairs of electrons on the oxygen atoms of these molecules. With carboxylic acids, thymol can either donate a hydrogen bond *via* its hydroxyl group or accept a hydrogen bond through its aromatic ring, depending on the specific acid's functional groups. Coutinho *et al.*^34^ measured the Kamlet–Taft solvatochromic parameters for mixtures of thymol with caprylic (octanoic), capric (decanoic), lauric (dodecanoic), myristic (tetradecanoic), palmitic (hexadecanoic), and stearic (octadecanoic) acids. They evaluated the *α* and *β* parameters describing the capacity for being HBDs and HBAs, respectively. They found that in the mixtures of thymol with these fatty acids (C8, C10, C12, C14, C16 and C18), thymol has a high capacity to donate H-bonds, which is largely unaffected by the length of the monocarboxylic acid chain. When mixed with alcohols like isobutanol, benzyl alcohol, and 1,2-decanediol, thymol can both donate and accept hydrogen bonds, creating a network of interactions that lead to a significant lowering of the melting point. Thymol also interacts with more complex molecules such as tetraheptylammonium bromide, trioctylphosphine oxide (TOPO), and ChCl, where it primarily functions as an HBD. This dual ability of thymol to donate and accept hydrogen bonds allows it to form DESs with a wide range of substances, leading to eutectic mixtures that have unique solvent properties suitable for various applications.

### 6.1 Stability of thymol-based DESs in aqueous environment

The water stability of a DES means the maintenance of the DES integrity as a distinct entity composed of HBA and HBD. In other words, the stability of a DES in water refers to the ability of the specific hydrogen bonding network between HBA and HBD components, which defines the DES structure and its unique properties, to persist upon the addition of water. A DES is water-stable if the hydrogen bonds between the HBA and HBD components significantly remain, even though water molecules are present and competing for hydrogen bonding sites. Instability occurs when water molecules effectively disrupt the HBA–HBD interactions, leading to the individual solvation of HBA and HBD components. The hydrophilicity/hydrophobicity of DES constituents significantly affects their water stability. For example, a DES composed of menthol (hydrophobic) and dodecanoic acid (hydrophobic) exhibits high stability in water. In contrast, a DES made from choline chloride (hydrophilic) and urea (hydrophilic) readily dissolves in water and loses its DES structure. It should be mentioned that DES stability in water is highly dependent on the water concentration. Most DESs can tolerate small amounts of water without significant disruption. At low water concentrations, the DES structure can remain largely intact, with water molecules integrating into the network. Commonly, small amounts of water are added to highly viscous hydrophilic DESs to enhance their fluidity.^260,261^ However, as the water concentration increases (beyond a certain threshold which varies greatly depending on the specific DES components), the interactions within the DES weaken, and eventually, the DES hydrophilic components become fully solvated by water, transitioning the system into an aqueous solution of the DES constituents.^261^

In addition to externally added water during application, the presence of residual water originating from the preparation process can significantly influence the physicochemical properties and structural organization of thymol-based DESs. During DES synthesis, incomplete removal of water from hygroscopic starting materials or insufficient drying of components may introduce water molecules into the eutectic network. Even at low concentrations, residual water can act as a competitive hydrogen-bonding partner and modify the strength and distribution of HBA–HBD interactions, thereby affecting key properties such as viscosity, density, polarity, conductivity, thermal stability, and extraction performance. In hydrophilic DESs, residual water is often incorporated into the hydrogen-bonding network and may reduce viscosity by increasing molecular mobility; however, excessive water can disrupt the eutectic structure by solvating the individual components. In contrast, hydrophobic thymol-based DESs generally show lower sensitivity to small amounts of residual water because their nonpolar constituents limit water incorporation, although trace water can still alter interfacial behavior and mass-transfer properties in extraction processes. Therefore, accurate determination and control of water content are essential for the reliable comparison of physicochemical data and for ensuring reproducibility of thymol-based DES preparation and applications. Karl Fischer titration is commonly recommended for precise quantification of water content, particularly when DES properties are correlated with thermodynamic or transport measurements.^262,263^

Hydrophobic DESs commonly maintain their structural integrity (the HBA-HBD network) in contact with water (even in high concentrations of water) because they do not mix noticeably with water, making them suitable for extracting non-polar compounds from aqueous phase. It has been suggested that the hydrophilic or hydrophobic nature of HBD dictates the overall hydrophobicity of the DESs.^128,226,264^ Notably, pH has been shown to exert a considerable influence on the aqueous solubility and, hence, the hydrophobicity of DESs.^49,50,59^ The binary DESs consisting of thymol and saturated medium-chain fatty acids (C6–C12) are hydrophobic and water-immiscible in the neutral aqueous environment. However, as the water environment is adjusted to alkaline by adding KOH, the thymol : fatty acid (C6–C12) DESs become miscible with water due to ionization of the DESs components, forming homogenous solutions. Conversely, adding acid (HCl) can neutralize the ionized groups of the DES components, returning the DES to its hydrophobic state and causing phase separation.^49,50,59^ It is noteworthy that FT-IR and ^1^H NMR spectroscopy of the DESs before and after pH-induced phase transition are similar, indicating the maintenance of the thymol-based hydrophobic DESs chemical structure after the conversion of water-miscibility and phase transition by altering pH.^50^ Further, it was shown that the FI-IR spectra of the *in situ* prepared thymol : octanoic acid DES by the addition of HCl to alkaline aqueous solution of the DES's precursors (thymol and octanoic acid) and the hydrophobic thymol : octanoic acid DES directly prepared by mixing its constituents through heating and stirring method are almost identical.^49^Table 2 addresses the stability or instability of thymol-based DESs in water based on literature data regarding hydrophobic or hydrophilic nature of these DESs. However, discrepancies may exist between the information reported in different sources. For example, Mostafa *et al.*^44^ employed thymol-acetic acid DES at various molar ratios (1 : 1, 1 : 2, 1 : 3, 1 : 4, and 2 : 1) as hydrophobic NDESs for dispersive liquid–liquid microextraction of sulfonamides in water samples, demonstrating the aqueous stability of this DES.^44^ Whereas, Duque *et al.*^96^ pointed out that the 1 : 1 and 5 : 1 molar ratios of thymol : acetic acid DES precipitate in water and the 1 : 10 molar ratio of thymol : acetic acid results in a hydrophilic DES.^96^ Although many literature used thymol : octanoic acid^26,34,48–50,55–58,60,62,104,114–116^ and thymol : decanoic acid^26,34,48–50,55–58,62^ DESs as a hydrophobic solvent in liquid–liquid extraction, González *et al.*^63^ reported a cloudy interphase for 50% v/v ratio of water and these DESs (thymol : octanoic acid (1 : 1) and thymol : decanoic acid (1 : 1)) after passing a night of the prepared mixtures and concluded that these DESs are not suitable for liquid–liquid extraction from aqueous media.^63^

### 6.2 Thymol-based DESs with carboxylic acids

Binary thymol-based DESs formed with carboxylic acids represent one of the most extensively investigated subclasses of hydrophobic and amphiphilic DESs. These systems are typically based on hydrogen bonding interactions between thymol, acting as the HBD or HBA, and the carboxylic acid.

In the case of short-chain carboxylic acids, such as formic, acetic, lactic, acrylic, pyruvic, maleic, methacrylic, butyric, citric, and polyacrylic acids, thymol predominantly acts as a HBA. However, for other investigated acids, both components may exhibit a dual HBD/HBA nature, participating in a more complex hydrogen-bonding network. The diversity of carboxylic acids, from short-chain aliphatic acids to long-chain fatty acids and multifunctional acids, results in a wide range of physicochemical properties, phase behaviors, and water stabilities.

### 6.3 Molar ratio and compositional diversity

A key feature of thymol : carboxylic acid DESs is the broad range of molar ratios investigated. For simple monocarboxylic acids such as formic,^44^ acetic,^44^ acrylic,^104,105^ and pyruvic acids,^131^ the most common ratio is 1 : 1 (thymol : acid). However, systems such as thymol : acetic acid^96^ and thymol : lactic acid^98^ have been studied over wide composition ranges, including ratios from thymol-rich (*e.g.*, 5 : 1) to acid-rich systems (*e.g.*, 1 : 10). For medium- and long-chain fatty acids (*e.g.*, hexanoic, heptanoic, octanoic, decanoic, and dodecanoic acids), multiple molar ratios have been systematically explored, often covering nearly the entire composition range.^34,46,50,60,67,73^ In these systems, eutectic compositions frequently lie near equimolar or slightly acid-rich regions, although significant variation exists depending on chain length and functionalization. Notably, for thymol : octanoic acid, eutectic compositions around 1 : 1.86–1 : 2.00 have been reported,^34,46^ while thymol : decanoic acid shows eutectic ratios near 1 : 1.23–1 : 1.27.^34,46^ For longer-chain acids such as stearic acid, the eutectic point shifts toward thymol-rich compositions (*e.g.*, 5.25 : 1),^34^ reflecting increased van der Waals interactions and reduced hydrogen bonding efficiency. Multifunctional and aromatic carboxylic acids (*e.g.*, levulinic, phenylacetic, and azelaic acids) also exhibit diverse molar ratios, often extending to highly asymmetric compositions such as 15 : 1–19 : 1 for thymol : azelaic acid,^125^ indicating weak or highly directional intermolecular interactions.

### 6.4 Solid–liquid equilibrium (SLE) phase behavior

SLE phase diagrams have been reported for a limited number of thymol–carboxylic acid systems, but they provide critical insight into the nature of eutectic formation. SLE phase diagrams have been reported for thymol : octanoic acid,^34,46^ thymol : decanoic acid,^34,46^ thymol : dodecanoic acid,^34^ thymol:tetradecanoic acid,^34^ thymol : hexadecanoic acid,^34^ thymol : octadecanoic acid,^34^ thymol:octadecenoic acid^123^ and thymol : phenylacetic acid.^28^ Systems such as thymol : octanoic acid, thymol : decanoic acid, thymol : dodecanoic acid, and thymol : oleic acid exhibit clear eutectic behavior with measurable deviations from ideality. These deviations, often negative, confirm the formation of deep eutectic systems driven by strong hydrogen bonding and dispersion interactions. In contrast, many systems (*e.g.*, thymol with formic, acetic, lactic, acrylic, and pyruvic acids) lack reported SLE data, limiting the ability to definitively classify them as DESs. In some cases (*e.g.*, thymol : 10-undecylenic acid), incomplete analysis of deviations from ideality prevents confirmation of DES behavior despite observed melting point depression.

### 6.5 Physicochemical properties

Table 3 summarizes the physicochemical properties reported for thymol : carboxylic acid DESs at different molar ratios, with emphasis on data obtained at 25 °C or near room temperature to facilitate comparison.

**Table 3:** Physicochemical properties reported for various thymol : carboxylic acid DESs at 25 °C (unless otherwise specified)

DES	Molar ratio	Melting point/°C	Density/g cm^−3^	Viscosity/mPa s	Surface tension/mN m^−1^	Other properties
Thymol : citric acid	1 : 1		0.936 (ref. 130)			Polarity/kcal mol^−1^: 51.45 (ref. 130)
Thymol : 2-hydroxypropanoic acid (lactic acid)	1 : 3		1.101 (ref. 100)			
Thymol : 2-hydroxypropanoic acid (lactic acid)	2 : 1		0.90 (ref. 101)	30.86 (ref. 101)	44.47 (ref. 101)	
Thymol : 2-hydroxypropanoic acid (lactic acid)	4 : 1		0.814 (ref. 130)			Polarity/kcal mol^−1^: 51.34 (ref. 130)
Thymol : 4-oxopentanoic acid (levulinic acid)	1 : 3		1.074 (ref. 100)			
Thymol : 4-oxopentanoic acid (levulinic acid)	1 : 1		1.03783 (ref. 106)	28.2 (ref. 106)		Refractive index: 1.48866 (ref. 106)
Thymol : butanoic acid (butyric acid)	1 : 1		0.97 (ref. 119)	5.98 (ref. 119)		
Thymol : hexanoic acid (caproic acid)	2 : 1		0.838 (ref. 110)			
Thymol : hexanoic acid (caproic acid)	1 : 1		0.862,^110^ 0.9643 at 20 °C (ref. 49)	11.38 at 20 °C (ref. 49)		
Thymol : hexanoic acid (caproic acid)	1 : 2		0.855 (ref. 110)			
Thymol : heptanoic acid (enanthic acid)	3 : 1		0.97 (ref. 26)			Polarity/kcal mol^−1^: 31.35 (ref. 26)
Thymol : heptanoic acid (enanthic acid)	2 : 1		0.97 (ref. 26)			Polarity/kcal mol^−1^: 31.34 (ref. 26)
Thymol : heptanoic acid (enanthic acid)	1 : 1	−21.9 (ref. 26)	0.95,^26^ 0.9560 at 20 °C (ref. 49)	8.25,^26^ 12.14 at 20 °C (ref. 49)		Polarity/kcal mol^−1^: 31.34 (ref. 26)
Thymol : heptanoic acid (enanthic acid)	1 : 2		0.95 (ref. 26)			Polarity/kcal mol^−1^: 31.32 (ref. 26)
Thymol : heptanoic acid (enanthic acid)	1 : 3		0.95 (ref. 26)			Polarity/kcal mol^−1^: 31.29 (ref. 26)
Thymol : octanoic acid (caprylic acid)	1 : 3		0.93 (ref. 91)	6.89 (ref. 91)		Polarity/kcal mol^−1^: 50.03 (ref. 91)
Thymol : octanoic acid (caprylic acid)	1 : 2	7 (ref. 121)	0.863,^110^ 0.92872,^46^ 0.91682 at 40 °C,^46^ 0.9316 (ref. 121)	7.820,^46^ 4.769 at 40 °C (ref. 46)	29.29,^46^ 28.43 at 40 °C (ref. 46)	Refractive index: 1.45779,^46^ 1.45147 at 40 °C (ref. 46)
						Speed of sound/m s^−1^: 1342.97,^46^ 1291.04 at 40 °C (ref. 26)
						Heat capacity/J mol^−1^ K^−1^: 298,^46^ 310 at 40 °C (ref. 46)
						Static permittivity: 3.427,^46^ 3.365 at 40 °C (ref. 46)
Thymol : octanoic acid (caprylic acid)	1 : 1.38	6.68 36	0.9020 at 60 °C (ref. 36)	2.83 at 60 °C (ref. 36)		
Thymol : octanoic acid (caprylic acid)	1 : 1.375		0.9301,^34^ 0.9140 at 45 °C (ref. 34)	8.00 ^34^, 4.17 at 45 °C (ref. 34)		
Thymol : octanoic acid (caprylic acid)	1 : 1	−8.3 ^26^, 19.22 ^79^, 19.22 (ref. 81)	0.96,^26^ 0.9517 at 20 °C,^49^ 0.830,^110^ 0.95,^119^ 0.93942,^46^ 0.92753 at 40 °C (ref. 46)	10.17 ^26^, 13.13 at 20 °C ^49^, 11.85 ^119^, 9.201,^46^ 5.278 at 40 °C ^46^, 15.167 at 4 °C (ref. 85)	29.58 ^46^, 28.08 at 40 °C (ref. 46)	Refractive index: 1.47336,^46^ 1.46695 at 40 °C (ref. 46)
						Polarity/kcal mol^−1^: 31.35 ^26^
						Speed of sound/m s^−1^: 1362.79 ^46^, 1311.23 at 40 °C (ref. 46)
						Heat capacity/J mol^−1^ K^−1^: 283 ^46^, 293 at 40 °C (ref. 46)
						Static permittivity: 3.902 ^46^, 3.782 at 40 °C (ref. 46)
						Electrical conductivity/µS cm^−1^: 0.006 (ref. 85)
Thymol : octanoic acid (caprylic acid)	2 : 1		0.870,^110^ 0.93809 at 40 °C (ref. 46)	5.745 at 40 °C (ref. 46)	29.36 at 40 °C (ref. 46)	Refractive index: 1.48238 at 40 °C (ref. 46)
						Speed of sound/m s^−1^: 1330.96 at 40 °C (ref. 46)
						Heat capacity/J mol^−1^ K^−1^: 301 at 40 °C (ref. 46)
						Static permittivity: 4.012 at 40 °C (ref. 46)
Thymol : nonanoic acid (pelargonic acid)	1 : 1	−1.9(ref. 26)	0.95,^26^ 0.9433 at 20 °C (ref. 49)	10.88,^26^ 15.19 at 20 °C (ref. 49)		Polarity/kcal mol^−1^: 31.32 (ref. 26)
Thymol : nonanoic acid (pelargonic acid)	2 : 1		0.934 (ref. 124)	2.43 (ref. 124)	24.546 (ref. 124)	
Thymol : decanoic acid (capric acid)	1 : 3	19.0 (ref. 37)	0.9272 (ref. 37)	10.4 (ref. 37)		
Thymol : decanoic acid (capric acid)	1 : 2	18.0 ^37^, 18.0 ^76^, 19.9 (ref. 87)	0.91811 ^46^, 0.90659 at 40 °C ^46^, 0.918 ^51^, 0.9365 ^37^, 0.918 ^75^, 0.9358 ^76^, 0.9163 (ref. 87)	11.187 ^46^, 6.569 at 40 °C ^46^, 11.13 ^51^, 10.8 (ref. 37)	29.71 ^46^, 28.64 at 40 °C ^46^, 29.29 (ref. 51)	Refractive index: 1.45913 ^46^, 1.45291 at 40 °C (ref. 46)
						Speed of sound/m s^−1^: 1362.29 ^46^, 1310.08 at 40 °C (ref. 46)
						Heat capacity/J mol^−1^ K^−1^: 333 ^46^, 344 at 40 °C (ref. 46)
						Static permittivity: 3.283 ^46^, 3.214 at 40 °C (ref. 46)
						Maximum miscibility of water in DES/w/w%: 4.4 (ref. 51)
Thymol : decanoic acid (capric acid)	1 : 1	11.5 ^26^, 17.0 ^37^, 18.83 ^72^, 17.1 ^76^, 19.3 ^87^, 18.86 (ref. 36)	0.91 ^26^, 0.9301 ^34^, 0.9147 at 45 °C ^34^, 0.9407 at 20 °C ^49^, 0.92983 ^46^, 0.91821 at 40 °C ^46^, 0.94 ^91^, 0.930,^51^ 0.9437 ^37^, 0.909 ^75^, 0.9296 ^70^, 0.9432 ^76^, 0.9316 ^87^, 0.9031 at 60 °C (ref. 36)	11.24 ^26^, 12.16 ^34^, 5.74 at 45 °C ^34^, 16.56 at 20 °C ^49^, 12.030 ^46^, 6.703 at 40 °C ^46^, 9.72 ^91^, 12.00 ^51^, 11.2 ^37^, 12.34 ^70^, 12.333 at 4 °C ^85^, 3.70 at 60 °C (ref. 36)	30.02 ^46^, 28.93 at 40 °C ^46^, 29.69 (ref. 51)	Refractive index: 1.47273 ^46^, 1.46637 at 40 °C (ref. 46)
						Polarity/kcal mol^−1^: 31.25 ^26^, 50.87 (ref. 92)
						Speed of sound/m s^−1^: 1374.37 ^46^, 1323.80 at 40 °C (ref. 46)
						Heat capacity/J mol^−1^ K^−1^: 317 ^46^, 327 at 40 °C (ref. 46)
						Static permittivity: 3.635 ^46^, 3.540 at 40 °C (ref. 46)
						Maximum miscibility of water in DES/w/w%: 4.1 ^51^, 3.28 (ref. 70)
						Electrical conductivity/µS cm^−1^: 1.241 (ref. 85)
Thymol : decanoic acid (capric acid)	1.5 : 1	18.0 ^37^, 18.7 ^76^	0.937 ^51^, 0.9515 ^37^, 0.9521 ^76^, 0.919 at 20 °C (ref. 82)	12.45 ^51^, 13.0 ^37^, 18.862 at 20 °C (ref. 82)	29.89 (ref. 51)	Maximum miscibility of water in DES/w/w%: 3.9 (ref. 51)
Thymol : decanoic acid (capric acid)	2 : 1	19.3 ^76^, 19.9 (ref. 87)	0.93060 at 40 °C ^46^, 0.942 ^51^, 0.925 ^75^, 0.9589 ^76^, 0.9502 (ref. 87)	6.738 at 40 °C ^46^, 12.84 (ref. 52)	29.16 at 40 °C ^46^, 30.07 (ref. 51)	Refractive index: 1.48087 at 40 °C (ref. 46)
						Speed of sound/m s^−1^: 1337.53 at 40 °C (ref. 46)
						Heat capacity/J mol^−1^ K^−1^: 334 at 40 °C (ref. 46)
						Static permittivity: 3.862 at 40 °C (ref. 46)
						Maximum miscibility of water in DES/w/w%: 3.6 (ref. 51)
Thymol : 2-hexyldecanoic acid	1 : 1		0.905 (ref. 126)	31.2 (ref. 126)		Maximum miscibility of water in DES/w/w%: 0.468 (ref. 126)
Thymol : 2-hexyldecanoic acid	1 : 2		0.892 (ref. 126)	32.9 (ref. 126)		Maximum miscibility of water in DES/w/w%: 0.689 (ref. 126)
Thymol : 2-hexyldecanoic acid	1 : 3		0.884 (ref. 126)	32.7 (ref. 126)		Maximum miscibility of water in DES/w/w%: 0.681 (ref. 126)
Thymol : 2-hexyldecanoic acid	1 : 4		0.882 (ref. 126)	33.5 (ref. 126)		Maximum miscibility of water in DES/w/w%: 1.21 (ref. 126)
Thymol : phenylacetic acid	3 : 1	3 (ref. 128)	0.9771 (ref. 128)			Maximum miscibility of water in DES/w/w%: 5.50 (ref. 128)
Thymol : 10-undecylenic acid	1 : 4	7.5 (ref. 37)	0.9353 (ref. 37)	11.8 (ref. 37)		
Thymol : 10-undecylenic acid	1 : 3	9.0 (ref. 37)	0.9375 (ref. 37)	12.4 (ref. 37)		
Thymol : 10-undecylenic acid	1 2	10.0 (ref. 37)	0.9395 (ref. 37)	13.1 (ref. 37)		
Thymol : 10-undecylenic acid	1 1	11.0 (ref. 37)	0.9457 (ref. 37)	13.2 (ref. 37)		
Thymol : 10-undecylenic acid	1.02 : 1.00		0.9395 (ref. 103)	13.79 (ref. 103)		
Thymol : 10-undecylenic acid	1.5 : 1	16.5 (ref. 37)	0.9515 (ref. 37)	14.4 (ref. 37)		
Thymol : 10-undecylenic acid	2.33 1	19.0 (ref. 37)	0.9598 (ref. 37)	15.6 (ref. 37)		
Thymol : octadecenoic acid (oleic acid)	1 : 1		0.913 (ref. 96)	23.4 (ref. 96)	31.6 (ref. 96)	Refractive index: 1.478 (ref. 96)
						Electrical conductivity/µS cm^−1^: 400 (ref. 96)
						Thermal conductivity/W m^−1^ K^−1^: 0.152 (ref. 96)
Thymol : octadecenoic acid (oleic acid)	1 : 5		0.895 (ref. 96)	27.1 (ref. 96)	31.7 (ref. 96)	Refractive index: 1.464 (ref. 96)
						Electrical conductivity/µS cm^−1^: 3600 (ref. 96)
						Thermal conductivity/W m^−1^ K^−1^: 0.160 (ref. 96)
Thymol : octadecadienoic acid (linoleic acid)	1 : 1		0.914 (ref. 96)	20.5 (ref. 96)	31.9 (ref. 96)	Refractive index: 1.478 (ref. 96)
						Electrical conductivity/µS cm^−1^: 2800 (ref. 96)
						Thermal conductivity/W m^−1^ K^−1^: 0.154 (ref. 96)
Thymol : octadecadienoic acid (linoleic acid)	1 : 5		0.901 (ref. 96)	21.9 (ref. 96)	32.1 (ref. 96)	Refractive index: 1.468 (ref. 96)
						Electrical conductivity/µS cm^−1^: 13400 (ref. 96)
						Thermal conductivity/W m^−1^ K^−1^: 0.160 (ref. 96)
Thymol : dodecanoic acid (lauric acid)	1 : 2		0.893 (ref. 75)			
Thymol : dodecanoic acid (lauric acid)	1 : 1	22.9 (ref. 59)	0.912 (ref. 75)	15.000 at 4 °C (ref. 85)		Electrical conductivity/µS cm^−1^: 1.208 (ref. 85)
Thymol : dodecanoic acid (lauric acid)	1.222 : 1	24.83 (ref. 36)	0.9107 at 45 °C ^34^, 0.8992 at 60 °C (ref. 36)	7.01 at 45 °C ^34^, 4.42 at 60 °C (ref. 36)		
Thymol : dodecanoic acid (lauric acid)	2 : 1		0.911 (ref. 75)			
Thymol : tetradecanoic acid (myristic acid)	3 : 1	38.16 (ref. 36)	0.9240 at 45 °C ^34^, 0.9126 at 60 °C (ref. 36)	7.16 at 45 °C ^34^, 4.34 at 60 °C (ref. 36)		
Thymol : hexadecanoic acid (palmitic acid)	4 : 1	41.22 (ref. 36)	0.9255 at 45 °C ^34^, 0.9140 at 60 °C (ref. 36)	7.54 at 45 °C ^34^, 4.53 at 60 °C (ref. 36)		
Thymol : octadecanoic acid (stearic acid)	9 : 1	46.22 (ref. 36)	0.9357 at 45 °C ^34^, 0.9240 at 60 °C (ref. 36)	6.88 at 45 °C ^34^, 4.06 at 60 °C (ref. 36)		

Although DESs composed of thymol and carboxylic acids have been studied much more extensively than other systems, the primary focus of the scientific literature, as with other systems, has been on density, viscosity, melting point, and surface tension; however, density and viscosity have received the most attention. Other properties, including refractive index, polarity, speed of sound, heat capacity, static permittivity, electrical and thermal conductivity, and water miscibility, have been investigated for only a limited number of systems. Thus, the available database is highly uneven, with insufficient systematic data to establish reliable structure–property relationships, particularly for less-studied properties. The investigated carboxylic acids cover several structural classes but are strongly dominated by aliphatic monocarboxylic acids, especially medium- and long-chain fatty acids such as butanoic, hexanoic, heptanoic, octanoic, nonanoic, decanoic, 10-undecylenic, dodecanoic, tetradecanoic, hexadecanoic, octadecanoic, oleic, and linoleic acids. Octanoic and decanoic acids have received the greatest attention. In contrast, hydroxycarboxylic, polyfunctional, keto, aromatic, and branched-chain acids are represented by only a few examples. Consequently, the current dataset is adequate for examining some chain-length effects but is insufficient to distinguish systematically the effects of functional groups, branching, unsaturation, and the number of carboxyl groups. Broader and more balanced acid selection is therefore needed.

Significant discrepancies are also evident among studies reporting identical DES compositions. For thymol : octanoic acid (1 : 2), densities at 25 °C range from 0.863 to 0.9316 g cm^−3^.^46,110,121^ For thymol : octanoic acid (1 : 1), reported densities and viscosities range from 0.830–0.96 g cm^−3^ and 9.201–11.85 mPa s, respectively.^26,46,49,110,119^ Similarly, thymol : decanoic acid (1 : 1) shows melting points of 11.5–19.3 °C, densities of approximately 0.909–0.9437 g cm^−3^, and viscosities of 9.72–12.34 mPa s across different studies.^26,34,36,37,72,76,87^ Polarity values for this system also differ substantially, from 31.25 (ref. 26) to 50.87 (ref. 91) kcal mol^−1^. Such discrepancies may arise from differences in water content, temperature, component purity, sample preparation, equilibration, and exact composition, emphasizing the need for standardized experimental protocols and explicit reporting of these parameters.

At a fixed thymol ratio, carboxylic acid structure substantially affects thermophysical properties. When examining data from a single systematic study to avoid inter-laboratory discrepancies, a clear trend of decreasing density with increasing chain length is observed for 1 : 1 DESs containing short-to-medium-chain acids. For instance, at 20 °C, the values of density decrease monotonically from 0.9643 g cm^−3^ for hexanoic acid to 0.9560 g cm^−3^ for heptanoic acid, 0.9517 g cm^−3^ for octanoic acid, 0.9433 g cm^−3^ for nonanoic acid, and 0.9407 g cm^−3^ for decanoic acid.^49^ In contrast, viscosity generally increases, from 5.98 mPa s (ref. 119) for thymol : butanoic acid to 8.25 mPa s (ref. 26) for thymol : heptanoic acid, 10.17 (ref. 26) or 11.85 (ref. 119), mPa s for thymol : octanoic acid, 10.88 mPa s (ref. 26) for thymol : nonanoic acid, and 11.24 (ref. 26) or 12.16 (ref. 34) mPa s for thymol : decanoic acid. This behavior reflects the increasing contribution of the hydrocarbon chain to dispersion interactions and molecular friction. The effect is stronger for unsaturated long-chain acids: at 1 : 1, the viscosities of the oleic- and linoleic-acid DESs reach 23.4 (ref. 96) and 20.5 (ref. 96) mPa s, respectively, compared with approximately 13.2 (ref. 37) mPa s for the 10-undecylenic-acid system, while their densities remain in the relatively narrow range of 0.913–0.9457 g cm^−3^.^37,96^ The melting point rises from −21.9 °C (ref. 26) for thymol : heptanoic acid (1 : 1) to −8.3 °C (ref. 26) for thymol : octanoic acid (1 : 1) and −1.9 °C (ref. 26) for thymol : nonanoic acid (1 : 1), followed by substantially higher values for longer saturated acids, *e.g.*, 11.5–19.3 °C for thymol : decanoic acid (1 : 1),^26,36,37,72,76,87^ 22.9 °C for thymol : dodecanoic acid (1 : 1),^59^ reflecting enhanced molecular packing and crystallization of longer saturated chains. Unsaturation tends to disrupt packing and lower melting temperatures. Overall, acid chain length has the clearest effect on viscosity and melting point, whereas density shows a weaker and more structure-dependent response. Other properties cannot yet be compared systematically because of limited data.

The thymol acid molar ratio also has a pronounced but system-dependent effect, and no universal trend can be established. Increasing the thymol fraction decreases density in thymol : lactic acid^100,101,130^ and thymol : 4-oxopentanoic acid,^100,106^ but increases density in thymol : 10-undecylenic acid,^37^ decanoic acid,^37^ oleic acid,^96^ linoleic acid,^96^ and 2-hexyldecanoic acid,^126^ systems. Some systems exhibit non-monotonic or weak composition dependence. For example, thymol : hexanoic acid shows densities of 0.838, 0.862, and 0.855 g cm^−3^ at ratios of 2 : 1, 1 : 1, and 1 : 2, respectively,^110^ while thymol : octanoic acid changes from 0.870 to 0.830 and then 0.863 g cm^−3^ over the same ratios.^110^ At 40 °C, however, monotonic decreases in density with increasing acid content were reported for both thymol : octanoic acid and thymol : decnoic acid^46^ DESs. These differences indicate that density depends on the relative molecular volumes and intrinsic densities of the components, as well as composition-dependent hydrogen bonding, molecular packing, and free volume. Viscosity likewise shows no universal dependence on molar ratio. Increasing the thymol fraction increases viscosity in thymol : 10-undecylenic acid,^37^ octanoic acid,^46^ and decanoic acid^37,46,51^ systems, whereas the opposite trend occurs for thymol : 2-hexyldecanoic acid,^126^ oleic acid,^96^ and linoleic acid.^96^ In the systematically investigated thymol : oleic and thymol : linoleic acid systems, changing the ratio from 1 : 1 to 1 : 5 decreases density and refractive index but slightly increases viscosity, thermal conductivity, and surface tension while markedly increasing electrical conductivity.^96^ The melting point also depends on composition: it decreases with increasing thymol fraction in thymol : decanoic acid^37,76^ but shows the opposite trend for thymol : 10-undecylenic acid.^37^

Overall, molar ratio is an important formulation parameter, but its effect depends strongly on the carboxylic-acid structure, intermolecular interactions, and the property considered. The substantial scatter among studies for nominally identical compositions further demonstrates the importance of controlling water content, temperature, purity, sample preparation, and measurement procedures. More systematic measurements across acid structures and molar ratios under identical experimental conditions are therefore required to establish reliable composition–property relationships for thymol-based DESs.

### 6.6 Water stability and hydrophobicity

Water stability is a crucial parameter for practical applications, especially in liquid–liquid extraction. Most thymol-based DESs with carboxylic acids exhibit good stability in aqueous environments, forming a separate hydrophobic phase. This behavior is particularly consistent for systems with medium- and long-chain acids such as hexanoic, octanoic, decanoic, and higher fatty acids. However, water stability strongly depends on composition and acid structure. For example, thymol : acetic acid systems display composition-dependent behavior so that thymol-rich mixtures (*e.g.*, 1 : 1 and 5 : 1) are hydrophobic but unstable in water, potentially leading to precipitation, whereas acid-rich mixtures (*e.g.*, 1 : 10) become hydrophilic.^96^ This trend illustrates that increasing the polarity of the DES enhances water miscibility while reducing phase separation capability. Short-chain acid systems generally show lower hydrophobicity, while long-chain fatty acid systems exhibit pronounced hydrophobic character and excellent phase separation. Multifunctional acids such as citric acid^129^ and polymeric acids (*e.g.*, polyacrylic acid)^133^ can also form hydrophobic water-stable DESs.

### 6.7 Preparation methods

The predominant method for preparing thymol–carboxylic acid DESs is simple heating and stirring of the components until a homogeneous liquid is formed. This method is widely applied across all systems, from short-chain acids to long-chain fatty acids. Alternative preparation approaches include ultrasonication-assisted heating, which enhances mass transfer and reduces preparation time,^78^ and *in situ* formation, where the DES is generated directly within the application medium.^26,49,109,125^ In some cases, preparation at room temperature with continuous stirring has also been reported, particularly for systems with low melting components.^80^

Overall, thymol-based DESs with carboxylic acids exhibit remarkable compositional flexibility and tunable physicochemical properties. The chain length and functionalization of the acid play a decisive role in determining eutectic composition, hydrophobicity, and thermophysical behavior. While extensive data are available for fatty acid-based systems, significant gaps remain for short-chain and multifunctional acids, particularly regarding SLE phase diagrams and comprehensive physicochemical characterization. Furthermore, the classification of some systems as true DESs remains uncertain due to the lack of thermodynamic analysis of non-ideality. Future studies should prioritize systematic SLE investigations, standardized property measurements, and detailed analysis of water interactions to fully elucidate the structure–property relationships governing these versatile solvent systems.

### 6.8 Thymol-based DESs with amides, amines, and nitrogen-containing heterocycles

Binary thymol-based DESs formed with amides, amines, and nitrogen-containing heterocycles represent an important subclass of hydrophobic and, in some cases, amphiphilic systems. These DESs are typically stabilized through hydrogen-bond interactions between thymol acting predominantly as a HBD and the paired compound (urea, acetamide, acetanilide, triethylenetetramine HCl, tetraethylenetetramine HCl, 4-(dimethylamino)benzaldehyde, 1,10-phenanthroline, lidocaine, caffeine, 1-(*o*-tolyl)thiourea and *N*,*N*-dimethylformamide) functioning as a HBA, although in some systems (thymol : monoethanolamine and thymol : *N*-methylethanolamine) both components may exhibit dual HBD/HBA behavior. The available literature provides valuable insights into their phase behavior, physicochemical properties, water stability, and preparation methods.

### 6.9 Molar ratios and phase behavior

A wide range of molar ratios has been explored for these systems, often covering the entire composition range to accurately determine eutectic points through SLE phase diagrams. For example, in the thymol : urea system, the eutectic composition was identified at a 5 : 1 molar ratio (*x*_thy_^E^ = 0.83) with a eutectic temperature of −5 °C,^128^ although other ratios such as 1 : 1, 1 : 2, and 3 : 1 have also been reported.^129^ Similarly, thymol : acetamide and thymol : acetanilide systems exhibit eutectic regions within relatively narrow molar ratio ranges of approximately (1.5–2.5) : 1 and (2–2.5) : 1, respectively, both showing significantly depressed eutectic temperatures (−61.95 °C and −57.85 °C),^135^ indicative of strong intermolecular interactions and pronounced non-ideal behavior.

For nitrogen-containing heterocycles, such as thymol : 1,10-phenanthroline, the eutectic composition spans a broader range ((2.33–4) : 1), suggesting flexibility in DES formation depending on composition.^136^ In contrast, thymol : lidocaine has been extensively studied, with multiple reported molar ratios (1 : 1, 2 : 1, and 1 : 2), and a well-defined eutectic point near equimolar composition (*x*_thy_^E^ ≈ 0.5).^141^ Some systems, such as thymol : 1-(*o*-tolyl)thiourea, exhibit eutectic behavior at highly asymmetric compositions (9 : 1),^146^ although the deviation from ideality has not always been assessed, leaving uncertainty regarding their classification as true DESs. It is noteworthy that for several systems (*e.g.*, thymol : monoethanolamine, *N*-methylethanolamine, triethylenetetramine.HCl, tetraethylpentamine HCl, 4-(dimethylamino)benzaldehyde, atropine, caffeine and *N*,*N*-dimethylformamide), no SLE data are available, limiting the ability to conclusively classify them as deep eutectic systems.

### 6.10 Physicochemical properties

Table 4 summarizes the physicochemical properties reported for thymol : amides/amines/nitrogen-containing heterocycle DESs at different molar ratios, with emphasis on data obtained at 25 °C or near room temperature to facilitate comparison.

**Table 4:** Physicochemical properties reported for various thymol : amides/amines/nitrogen-containing heterocycle DESs at 25 °C (unless otherwise specified)

DES	Molar ratio	Density g cm^−3^	Viscosity mPa s	Other properties
Thymol–urea	5 : 1	0.9794 (ref. 128)		Melting point/°C: −5 (ref. 128)
				Maximum miscibility of water in DES/w/w%: 2.35 (ref. 128)
Thymol–monoethanolamine	1 : 3			Melting point/°C: −81.5 (glass transition)^145^
Thymol–acetamide	2 : 1	0.9905 (ref. 135)	39.96 (ref. 135)	Refractive index: 1.5027 (ref. 135)
				Melting point/°C: −61.95 (ref. 135)
				Speed of sound/m s^−1^: 1467.03 (ref. 135)
Thymol–acetanilide	2 : 1	1.0096 (ref. 135)	46.59 (ref. 135)	Refractive index: 1.5363 (ref. 135)
				Melting point/°C: −57.85 (ref. 135)
				Speed of sound/m s^−1^: 1475.71 (ref. 135)
Thymol–triethylenetetramine hydrochloride	3 : 1	1.056 ^143^, 1.0317 at 50 °C (ref. 144)	201.73 ^143^, 205.1 at 50 °C (ref. 144)	
Thymol–triethylenetetramine hydrochloride	5 : 1	1.0230 at 50 °C (ref. 144)	69.1 at 50 °C (ref. 144)	
Thymol–tetraethylpentamine hydrochloride	3 : 1	1.0513 at 50 °C (ref. 144)	295.7 at 50 °C (ref. 144)	
Thymol–tetraethylpentamine hydrochloride	5 : 1	1.0421 at 50 °C (ref. 144)	198.8 at 50 °C (ref. 144)	
Thymol-4-(dimethylamino)benzaldehyde	1 : 1	1.030 (ref. 134)	34.6 at 30 °C (ref. 134)	
Thymol-1,10-phenanthroline	4 : 1	1.0239 (ref. 136)	75.312 (ref. 136)	Melting point/°C: 7.35 (ref. 136)
Thymol–lidocaine	1 : 2	1.00532 (ref. 141)	202.84 (ref. 141)	
Thymol–lidocaine	1 : 1	0.99312,^138^ 0.99437,^138^^a^ 0.99312 (ref. 141)	177.15,^138^ 149.80,^138^^a^ 177.15 (ref. 141)	Maximum miscibility of water in DES/w/w%: 1.77 at 22 ^138^, 0.02960 at 22 °C (ref. 138)^b^
Thymol–lidocaine	2 : 1	0.9891,^138^ 0.99085,^138^^a^ 0.99 at 30 °C ^142^, 0.98908 (ref. 141)	122.05 ^138^, 100.20,^138^^a^ 122 at 30 °C ^142^, 122.05 (ref. 141)	Maximum miscibility of water in DES/w/w%: 1.64 at 22,^138^ 0.02551 at 22 °C,^138^^b^ 0.0610 at 30 °C (ref. 142)^b^
Thymol–atropine	2 : 1	1.06229 ^138^, 1.06102 (ref. 138)^a^	86800 138, 9255.5 (ref. 138)^a^	Maximum miscibility of water in DES/w/w%: 3.00 at 22,^138^ 0.11056 at 22 °C (ref. 138)^b^
Thymol-1-(*o*-tolyl)thiourea	9 : 1	0.990031 (ref. 146)	25.34 (ref. 146)	

a Density and viscosity of the water saturated DES.

b Water content before contact with water.

The physicochemical characterization of thymol-based DESs containing amides, amines, and nitrogen-containing heterocyclic compounds remains relatively limited and uneven. The properties reported to date include density, viscosity, refractive index, melting point, speed of sound, and water miscibility, although most systems have been characterized for only a small subset of these properties. Density and viscosity are the most frequently reported properties, particularly for thymol : lidocaine, whereas refractive index, melting point, speed of sound and water miscibility have been reported for only a few systems. In particular, important transport and thermodynamic properties such as heat capacity, polarity, surface tension, dielectric response, thermal and electrical conductivity, have not been systematically measured across comparable series, making it difficult to establish reliable structure–property relationships.

The studied HBA/partner structures encompass relatively simple amides, represented by urea, acetamide, and acetanilide,^128,135^ alkanolamines such as monoethanolamine,^145^ polyamines in the form of triethylenetetramine hydrochloride and tetraethylpentamine hydrochloride,^143,144^ and nitrogen-containing aromatic or heterocyclic compounds including 4-(dimethylamino)benzaldehyde, 1,10-phenanthroline, lidocaine, atropine, and 1-(*o*-tolyl)thiourea.^134,136,138,141,142,146^ However, the literature covers only a narrow fraction of the possible structural diversity of amides and amines: systematic studies of primary, secondary, and tertiary amides; homologous aliphatic amines; amino alcohols beyond monoethanolamine; aromatic amines; cyclic amines; and structurally related heterocycles are largely absent, while comparisons within a given chemical class are generally unavailable.

As shown in the table, density and viscosity data for the thymol : lidocaine system have been reported by two sources^138,141^ under identical conditions, and the values show generally good agreement. For the 1 : 1 molar ratio, both sources report a density of 0.99312 g cm^−3^, whereas for the 2 : 1 ratio, the reported densities are 0.9891 and 0.98908 g cm^−3^, respectively. Similarly, the reported viscosity values are 177.15 and 122.05 mPa s for the 1 : 1 and 2 : 1 molar ratios, respectively. Overall, the close agreement between the independently reported values supports the reliability and reproducibility of the density and viscosity data for the thymol : lidocaine system.

Regarding molar-ratio effects, the thymol : lidocaine series provides the clearest comparison under essentially comparable conditions: increasing the thymol fraction from 1 : 2 to 1 : 1 and then 2 : 1 decreases the density from 1.00532 to 0.99312 and 0.98908 g cm^−3^, respectively, while the viscosity decreases from 202.84 mPa s at 1 : 2 to 177.15 mPa s at 1 : 1 and 122.05 mPa.s at 2 : 1,^141^ indicating that increasing thymol content progressively lowers both density and viscosity in this series. A similar ratio dependence is suggested for triethylenetetramine hydrochloride and tetraethylpentamine hydrochloride, for which increasing the thymol proportion from 3 : 1 to 5 : 1 decreases density and viscosity;^144^ increasing the thymol fraction from 1 : 1 to 2 : 1 decreases the maximum water miscibility of the DES from 1.77 to 1.64 wt%, respectively. Since water is denser than thymol : lidocaine, water incorporation increases the density of the water-saturated DES relative to that of the freshly prepared, water-free DES. Conversely, because water has a much lower viscosity than thymol : lidocaine, water addition reduces the viscosity of the DES.^138^ As shown in the table, the water content increases after mixing the DESs with water, confirming the incorporation of water into the DES structure.

The chemical structure of the amide, amine, or nitrogen-containing heterocycle strongly influences the thermophysical properties of thymol-based DESs, although direct comparisons under identical conditions are limited. Under identical 2 : 1 conditions, replacing acetamide with the bulkier aromatic acetanilide increases density (0.9905 → 1.0096 g cm^−3^), viscosity (39.96 → 46.59 mPa s), refractive index, and speed of sound, suggesting stronger intermolecular interactions and molecular packing.^135^ Larger and more multifunctional nitrogen-containing compounds can produce even higher viscosities; for example, thymol : atropine (2 : 1) shows a much higher viscosity (86,800 mPa s) than thymol : lidocaine (122.05 mPa s) at the same ratio.^138^ Similarly, at 50 °C, thymol : tetraethylpentamine hydrochloride is denser and more viscous than thymol : triethylenetetramine hydrochloride, indicating an effect of amine structure and functionality.^144^ Overall, larger, more rigid, aromatic, or multifunctional nitrogen-containing components generally tend to increase density and particularly viscosity, but the available data are limited by differences in temperature, composition, and water content, making systematic structure–property comparisons necessary.

### 6.11 Water stability

Most systems in this category, including thymol : urea,^128,129^ thymol : 4-(dimethylamino)benzaldehyde,^134^ thymol : triethylenetetramine hydrochloride,^143^ thymol : tetraethylpentamine hydrochloride,^144^ thymol : acetamide,^135^ thymol : acetanilide,^135^ thymol : 1,10-phenanthroline,^136,137^ thymol : lidocaine,^108,138–141^ thymol : N,N-dimethylformamide,^147^ thymol : atropine,^138^ and thymol : 1-(*o*-tolyl)thiourea, are reported to be stable in the presence of water. This stability is often associated with their hydrophobic nature and the strength of intermolecular interactions. However, detailed quantitative assessments of water stability are often lacking, and in some cases, stability is inferred from successful application in aqueous systems rather than direct experimental measurements. For a few systems, no information on water stability is available (*e.g.*, thymol : monoethanolamine and thymol : *N*-methylethanolamine), indicating the need for further investigation.

### 6.12 Preparation methods

The predominant method for preparing thymol-based DESs in this class is the conventional heating and stirring technique. This method has been successfully applied to nearly all reported systems. Alternative preparation strategies have also been reported for specific systems. For example, thymol : lidocaine DESs have been prepared not only by heating and stirring but also by simple mixing without external energy input,^139,140^ and by rotary evaporation at low temperature (20 °C) without solvent.^142^

Overall, thymol-based DESs with amides, amines, and nitrogen-containing heterocycles exhibit diverse phase behavior and physicochemical properties, largely governed by the nature of the hydrogen bond acceptor and the molar ratio. Systems with well-characterized SLE diagrams and significant melting point depression (*e.g.*, thymol : acetamide,135 thymol : acetanilide^135^ and thymol : lidocaine^141^) can be confidently classified as DESs. However, for many systems (such as thymol : monoethanolamine, thymol : *n*-methylethanolamine, thymol : triethylenetetramine hydrochloride, thymol : tetraethylpentamine hydrochloride, thymol : 4-(dimethylamino)benzaldehyde, thymol : atropine, thymol : caffeine, and thymol : n,n-dimethylformamide) lacking phase diagram analysis, the classification remains tentative. Furthermore, while most systems demonstrate promising water stability and tunable physicochemical properties, the scarcity of systematic studies, particularly regarding long-term stability, thermodynamic non-ideality, and structure–property relationships, highlights the need for more comprehensive investigations.

### 6.13 Thymol-based DESs with alcohols and diols

Thymol-based DESs formed with alcohols and diols represent an important subclass of predominantly hydrophobic eutectic systems, where thymol can act either as a HBD (thymol : tetradecanol, thymol : hexadecanol and thymol : octadecanol), HBA (thymol : hexafluoroisopropanol), or both (other investigated systems shown in Table 2), depending on the partner molecule. These systems are typically characterized by hydrogen-bonding interactions between the hydroxyl group of thymol and the –OH functionalities of alcohols/diols, with the strength and extent of these interactions strongly influenced by chain length, branching, and functionalization of the co-component.

### 6.14 Molar ratios and composition range

A wide range of molar ratios has been explored for thymol–alcohol/diol systems, reflecting the compositional flexibility required to achieve eutectic behavior. For short-chain diols such as ethylene glycol, multiple molar ratios including 1 : 3, 1 : 2, 1 : 1, 2 : 1, and 3 : 1 have been reported,^148^ indicating extensive screening to identify optimal compositions. Similarly, thymol : hexafluoroisopropanol systems have been investigated at 1 : 2, 1 : 3, and 1 : 4 ratios,^153^ and thymol : isobutanol mixtures span 1 : 1 to 3 : 1.^43^ For longer-chain alcohols, the molar ratios also vary significantly. For example, thymol : octanol and thymol : decanol systems have been studied across broad composition ranges (*e.g.*, 1 : 2 to 3 : 1 and up to 5 : 1, respectively).^90,147^ In contrast, some systems such as thymol : 1,4-butanediol,^71^ thymol : 1,2-decanediol,^138^ and thymol : 2-hydroxyethyl methacrylate^156^ have been primarily reported at a single molar ratio, typically 1 : 1 or 2 : 1. The polymeric alcohol PEG400 has also been used to form eutectic mixture with thymol at a single molar ratio 1 : 1.^43^

### 6.15 Solid–liquid equilibria phase behavior

The availability of SLE phase diagrams for thymol-based DESs with alcohols and diols is relatively limited. Most systems lack detailed SLE characterization, making it difficult to conclusively classify them as DESs based on thermodynamic criteria. Notable exceptions include thymol : tetradecanol, thymol : hexadecanol, and thymol : octadecanol systems, for which the entire composition range has been investigated.^150^ These studies identified well-defined eutectic points at molar ratios of approximately 1.2 : 1 (*x*_thy_^E^ ≈ 0.54, *T*^E^ ≈ 25 °C), 2.2 : 1 (*x*_thy_^E^ ≈ 0.69, *T*^E^ ≈ 35 °C), and 3.4 : 1 (*x*_thy_^E^ ≈ 0.77, *T*^E^ ≈ 38 °C) for thymol : tetradecanol, thymol : hexadecanol, and thymol : octadecanol respectively.^150^ These results demonstrate that increasing alkyl chain length shifts the eutectic composition toward higher thymol content and higher eutectic temperatures. For the majority of other systems, the absence of SLE data limits the ability to evaluate deviations from ideality and confirm deep eutectic behavior.

### 6.16 Physicochemical properties

Table 5 summarizes the reported physicochemical properties of thymol : alcohol/diol DESs at various molar ratios, focusing primarily on data obtained at 25 °C or near room temperature to facilitate meaningful comparison.

**Table 5:** Physicochemical properties of thymol : alcohol/diol DESs at 25 °C (unless otherwise specified)

DES	Molar ratio	Density/g cm^−3^	Viscosity/mPa s	Other properties
Thymol : ethylene glycol	2 : 1			Melting point/°C: 21.5 (ref. 148)
Thymol : hexafluoroisopropanol	1 : 2	1.265 at 40 °C (ref. 153)	26.1 (ref. 153)	Melting point/°C: −19.1 (ref. 153)
Thymol : hexafluoroisopropanol	1 : 3	1.390 at 40 °C (ref. 153)	11.4 (ref. 153)	Melting point/°C: −12.3 (ref. 153)
Thymol : hexafluoroisopropanol	1 : 4	1.447 at 40 °C (ref. 153)	9.7 (ref. 153)	Melting point/°C: −22.4 (ref. 153)
Thymol : octanol	1 : 2	0.877 (ref. 124)	3.78 (ref. 124)	Surface tension/mN m^−1^: 24.795 (ref. 124)
Thymol : 1,2-decanediol	2 : 1	0.95233,^138^ 0.95631 (ref. 138)^a^	64.25,^138^ 42.57 (ref. 138)	Maximum miscibility of water in DES/w/w%: 5.14 at 22,^138^ 0.03509 at 22 °C (ref. 138)^b^
Thymol : hexadecanol (cetyl alcohol)	1 : 1	0.728 (ref. 130)		Polarity/kcal mol^−1^: 52.15 (ref. 130)
Thymol : benzyl alcohol	2 : 1	0.985 (ref. 124)	4.87 (ref. 124)	Surface tension/mN m^−1^: 34.392 (ref. 124)

a Density and viscosity of the water saturated DES.

b Water content before contact with water.

The physicochemical characterization of thymol : alcohol/diol DESs remains relatively limited and uneven. To date, the reported properties include density, viscosity, melting point, surface tension, polarity, and water content/miscibility; however, available data focus primarily on density and viscosity, and to a lesser extent on the melting point, while other properties have been reported for only isolated systems. This represents a substantial data gap, particularly for transport and interfacial properties such as thermal/electrical conductivity, speed of sound, heat capacity, and surface tension, as well as dielectric and optical properties. The alcohol/diol partners investigated so far are also highly diverse but poorly represented, including ethylene glycol, hexafluoroisopropanol, 1-octanol, 1,2-decanediol, cetyl alcohol (1-hexadecanol), and benzyl alcohol. Nevertheless, systematic studies across homologous alcohol series, branched *versus* linear alcohols, aromatic *versus* aliphatic alcohols, and diols/polyols are largely absent, making it difficult to isolate the effect of alcohol structure. Direct comparison of identical systems across independent sources is likewise severely constrained because the table contains essentially no identical thymol : alcohol/diol systems reported under the same conditions by different references. Within the available data, the clearest compositional comparison is thymol : hexafluoroisopropanol, for which increasing the alcohol fraction from 1 : 2 to 1 : 4 decreases viscosity from 26.1 to 9.7 mPa s and gives melting points of −19.1, −12.3, and −22.4 °C for the 1 : 2, 1 : 3, and 1 : 4 compositions, respectively, whereas the reported densities (at 40 °C) increase from 1.265 to 1.447 g cm^−3^.^153^ For thymol : 1,2-decanediol (2 : 1), contact with water increases the density from 0.95233 to 0.95631 g cm^−3^ only slightly, but reduces the viscosity markedly from 64.25 to 42.57 mPa s, demonstrating the strong plasticizing effect of water; the initially prepared DES contained only 0.03509 water-content fraction (reported at 22 °C), whereas the water-saturated values represent a different hydration state.^138^ Comparisons among different alcohol structures suggest that alcohol identity can strongly influence the properties—for example, thymol : octanol (1 : 2) has a density of 0.877 g cm^−3^ and viscosity of 3.78 mPa s, whereas thymol : benzyl alcohol (2 : 1) shows higher values of 0.985 g cm^−3^ and 4.87 mPa s (ref. 124)—but these differences cannot be attributed solely to molecular structure because the molar ratios are not identical. Overall, the literature is characterized by sparse and non-systematic measurements, and controlled studies varying alcohol structure, thymol : alcohol ratio, and precisely defined water content under identical temperature and experimental conditions are needed to establish reliable structure–property relationships for these DESs.

### 6.17 Water stability

Most thymol-based DESs with alcohols and diols exhibit good hydrophobicity and stability in aqueous environments. Systems such as thymol : ethylene glycol,^133,148^ thymol : hexafluoroisopropanol,^153^ thymol : isobutanol,^43^ thymol : 1,4-butanediol,^71^ thymol : octanol,^55,90,124,154^ thymol : decanol,^55,147^ thymol : 1,2-decanediol,^138^ thymol : dodecanol,^44,157^ thymol : tetradecanol,^151,152^ thymol : benzyl alcohol,^43,55,124,154^ thymol : PEG200 (ref. 133) are reported to be stable in water. An important exception is thymol : PEG400 (1 : 1), which is unstable in water, undergoing phase separation where PEG400 partitions into the aqueous phase and thymol crystallizes.^43^ In contrast, thymol : PEG200 mixtures demonstrate good water stability and have been successfully applied in microextraction processes,^133^ highlighting the role of molecular weight of polymeric alcohols and chain flexibility in water-stability of the prepared thymol : PEG eutectic mixtures. For some systems, such as thymol : hexadecanol and thymol : octadecanol, water stability has not been reported. Overall, increasing hydrophobicity of the alcohol component (*e.g.*, longer alkyl chains) generally enhances water immiscibility and stability of the DES.

### 6.18 Preparation methods

All reported thymol-based DESs with alcohols and diols have been prepared using a conventional heating and stirring method. This approach involves mixing the components at the desired molar ratio and heating the mixture until a homogeneous, transparent liquid is formed. The simplicity and reproducibility of this method make it the most widely employed preparation technique across all systems, regardless of the nature of the alcohol or diol component.

Despite the wide variety of thymol-based DESs with alcohols and diols, several limitations are evident. First, the lack of comprehensive SLE phase diagrams for most systems restricts rigorous thermodynamic classification. Second, physicochemical property data are sparse and unevenly distributed across different systems. Third, although water stability is generally favorable, systematic studies correlating structure, molar ratio, and aqueous behavior are still lacking. Nevertheless, these systems demonstrate significant potential, particularly due to their tunable hydrophobicity, relatively simple preparation, and suitability for extraction applications. Future work should prioritize systematic phase behavior studies, complete physicochemical characterization, and deeper investigation into structure–property relationships.

### 6.19 Thymol-based DESs with terpenoids (oxygenated derivative of terpenes)

Terpenoids (oxygenated derivatives of terpenes) constitute one of the most extensively explored classes of HBAs or co-components in thymol-based DESs. These systems are particularly attractive due to their natural origin, hydrophobicity, low toxicity, and structural diversity, which enable fine-tuning of physicochemical and thermodynamic properties. As summarized in Table 2, a wide variety of thymol–terpenoid binary systems have been reported, including combinations with borneol, fenchol, terpineol, menthol, camphor, carvone, cineole, and others. In the thymol : borneol, fenchol, terpineol, β-citronellol, menthol, camphor, carvone, and cineole DESs, thymol acts as an HBD, however in the thymol : sobrerol, linalool, geraniol and carvacrol DESs, both components may exhibit a dual HBD/HBA nature.

### 6.20 Molar ratios and phase behavior

The molar ratio of thymol to terpenoid plays a decisive role in determining the formation and classification of DESs. In many cases, full SLE phase diagrams have been constructed to identify eutectic compositions and evaluate deviations from ideality. For thymol : borneol systems, two studies investigated the entire composition range, reporting the eutectic point close to 1 : 1 molar ratio (*x*_th*y*_^E^ ≈ 0.5) with an eutectic temperature (*T*^E^) around 10.55 °C in one study,^159^ while the other study reported slightly shifted composition (*e.g.*, 1.4 : 1 molar ratio, *x*_thy_^E^ = 0.583, *T*^E^ = 20.05 °C).^160^ Similarly, thymol : fenchol exhibits a eutectic point near 1 : 1.5 (*x*_thy_^E^ ≈ 0.4),^164^ whereas thymol : sobrerol shows a eutectic composition around 2.33 : 1 (*x*_thy_^E^ ≈ 0.7, *T*^E^ ≈ 27 °C).^159^ Among all systems, thymol : menthol is by far the most extensively studied. Numerous SLE investigations across the full composition range consistently indicate eutectic compositions near equimolar ratios (*x*_thy_^E^ ≈ 0.45–0.50), with remarkably low eutectic temperatures, often reaching −50 to −73 °C.^38,182,185,196^ However, some reports suggest higher eutectic temperatures (*e.g.*, 17 °C at 1 : 2.33 molar ratio),^154^ highlighting discrepancies that may arise from experimental conditions or purity. Based on strong negative deviations of SLE phase diagram from ideality obtained in several studies,^38,182,185,196^ thymol : menthol eutectic mixture is classified as a DES.

Thymol : camphor systems also exhibit diverse eutectic behavior, with eutectic compositions ranging from approximately 1 : 1 to 1 : 1.5 and temperatures spanning from −73 °C to 16 °C depending on the study.^154,159,160^ These changes and the non-repeatability of thermophysical properties may be related to differences in experimental methods or experimental error, and they emphasize the need for more precise measurements to obtain accurate and repeatable results. For several systems such as thymol:terpineol, thymol : β-citronellol, thymol : carvone, thymol : cineole, thymol : linalool, thymol : geraniol, and thymol : carvacrol complete SLE phase diagrams are not available. In such cases, eutectic compositions are either experimentally approximated or predicted (*e.g.*, COSMO-RS prediction for thymol : carvone and thymol : cineole suggesting a 1 : 1 eutectic ratio with extremely low eutectic temperatures).^179^

### 6.21 Physicochemical properties

Table 6 summarizes the physicochemical properties reported for thymol : terpenoids DESs at different molar ratios, with emphasis on measurements at 25 °C or near room temperature. Research has focused mainly on density, viscosity, melting point, surface tension, and water miscibility, with density and viscosity being the most extensively investigated. Other properties, including refractive index, polarity, speed of sound, heat capacity, static permittivity, and electrical and thermal conductivity, have been reported for only a limited number of systems. Water content has also been determined both before and after contact with aqueous media. Consequently, the available database remains uneven, limiting the development of reliable structure–property relationships, particularly for less-studied properties.

**Table 6:** Physicochemical properties reported for various thymol : terpenoid (oxygenated derivative of terpenes) DESs at 25 °C (unless otherwise specified)

DES	Molar ratio	Melting point/°C	Density/g cm^−3^	Viscosity/mPa s	Surface tension/mN m^−1^	Maximum miscibility of water in DES/w/w%	Other properties
Thymol : borneol	1 : 1		0.80,^158^ 0.9631 at 35 °C (ref. 159)	70.50,^158^ 43.10 at 35 °C (ref. 159)		0.018,^158^ 0.011 (ref. 158)^b^	
Thymol : borneol	2 : 1		0.81 (ref. 158)	44.34 (ref. 158)		0.015,^158^ 0.010 (ref. 158)^b^	
Thymol : borneol	3 : 1		0.83 (ref. 158)	31.93 (ref. 158)		0.011,^158^ 0.005 (ref. 158)^b^	
Thymol : borneol	4 : 1		0.84 (ref. 158)	22.41 (ref. 158)		0.007,^158^ 0.002 (ref. 158)^b^	
Thymol : borneol	2.33 : 1		0.9716 (ref. 160)	45.63 (ref. 160)	31.75 (ref. 160]		Refractive index: 1.5105 (ref. 160)
Thymol : fenchyl alcohol (fenchol)	1 : 1.5		0.967 (ref. 164)	32.90 (ref. 164)			
Thymol : terpineol	1 : 1.3		0.959 (ref. 164)	35.40 (ref. 164)			
Thymol : β-citronellol	1 : 1		0.911 (ref. 181)	17.00 (ref. 181)			
Thymol : menthol	1 : 4.88		0.908 (ref. 51)	52.54 (ref. 51)	28.69 (ref. 51)	1.3 (ref. 51)	
Thymol : menthol	1 : 4		0.910,^51^ 1.002 (ref. 192)	51.36,^51^ 79.69 at 20 °C (ref. 192)	28.82,^51^ 24.97 (ref. 192)	1.3 (ref. 51)	Heat capacity/J mol^−1^ K^−1^: 297.57 (ref. 192)
Thymol : menthol	1 : 3	−54.0 (ref. 203)	0.914,^51^ 0.911 (ref. 100)	48.99 (ref. 51)	28.87 (ref. 192)	1.3 (ref. 51)	
Thymol : menthol	1 : 2	−54.7 (ref. 203)	0.920,^51^ 0.924 at 20 °C,^82^ 0.92384,^138^ 0.92556,^138^^a^ 1.098,^191^ 0.92109 (ref. 182)	42.16,^51^ 54.685 at 20 °C,^82^ 67.85,^138^ 52.17,^138^^a^ 68.94 at 20 °C,^192^ 41.048 (ref. 182)	29.01,^51^ 27.97,^192^ 29.21 (ref. 182)	1.1,^51^ 1.69 at 22 °C,^138^ 0.03136 at 22 °C (ref. 138)^b^	Refractive index: 1.47903 (ref. 182)
							Speed of sound/m s^−1^: 1386.99 (ref. 182)
							Heat capacity/J mol^−1^ K^−1^: 296.52,^192^ 317 (ref. 182)
							Static permittivity: 5.857 (ref. 182)
							Electrical conductivity/µS cm^−1^: 0.011 at 40 °C (ref. 211)
Thymol : menthol	1 : 1.5		0.93 (ref. 91)	27.47 (ref. 91)			Polarity/kcal mol^−1^: 52.08 (ref. 91)
Thymol : menthol	1 : 1	−58.65,^166^ −54.9 (ref. 203)	0.933,^51^ 0.93322,^106^ 0.94,^26^ 0.935 at 20 °C,^82^ 0.9327,^159^ 0.93656,^138^ 0.93812,^138^^a^ 0.93293,^182^ 0.93302 ,^166^ 0.933,^201^ 0.934,^164^ 0.87 (ref. 101)	58.6,^106^ 36.06,^51^ 37.863 at 20 °C,^82^ 38.08,^159^ 53.14,^138^ 42.01,^138^^a^ 35.275,^182^ 162.12,^201^ 42.30,^164^ 28.37 (ref. 101)	29.72,^51^ 29.70,^182^ 38.35 (ref. 101)	1.1,^51^ 1.81 at 22 °C,^138^ 0.03068 at 22 °C,^138^^b^ 0.24 (ref. 201)^b^	Refractive index: 1.48906,^106^ 1.48953 (ref. 182)
							Polarity/kcal mol^−1^: 47.39 (ref. 26)
							Speed of sound/m s^−1^: 1396.13 (ref. 182)
							Heat capacity/J mol^−1^ K^−1^: 319 (ref. 182)
							Static permittivity: 5.659 (ref. 182)
							Electrical conductivity/µS cm^−1^: 0.042 at 40 °C (ref. 211)
Thymol : menthol	1.01 : 1.00		0.9328 (ref. 103)	12.84 at 40 °C (ref. 103)			
Thymol : menthol	2 : 1	−56.7 (ref. 203)	0.945,^51^ 0.94559,^182^ 0.9476 at 20 °C,^197^ 0.938 (ref. 124)	27.50,^51^ 26.102,^182^ 9.13 (ref. 124)	30.30,^51^ 30.32,^182^ 26.419 (ref. 124)	2.4 (ref. 51], 0.014 (ref. 197)^b^	Refractive index: 1.49914 (ref. 182)
							Speed of sound/m s^−1^: 1406.03 (ref. 182)
							Heat capacity/J mol^−1^ K^−1^: 319 (ref. 182)
							Static permittivity: 55.329 (ref. 182)
Thymol : menthol	3 : 1	−57.7 (ref. 203)	0.952 (ref. 51)	23.34 (ref. 51)	30.68 (ref. 51)	2.3 (ref. 51)	
Thymol : menthol	4 : 1		0.955 (ref. 51)	21.27 (ref. 51)	30.93 (ref. 51)	3.8 (ref. 51)	
Thymol : menthol	4.88 : 1		0.958 (ref. 51)	20.16 (ref. 51)	31.14 (ref. 51)	4.6 (ref. 51)	
Thymol : camphor	1 : 1.5		0.968 (ref. 164)	31.70 (ref. 164)			
Thymol : camphor	1 : 1	−44.0,^37^ −44.15,^166^ −77.66 (ref. 66)	0.98,^26^ 0.9873,^37^ 0.9668,^159^ 0.9675,^160^ 0.96698 (ref. 166)	25.8,^37^ 20.80,^159^ 21.10 (ref. 160)	30.35 (ref. 160)		Refractive index: 1.4970 (ref. 160)
							Polarity/kcal mol^−1^: 49.77 (ref. 26)
Thymol : camphor	1.5 : 1	−37.0 (ref. 37)	0.9732 (ref. 37)	20.5 (ref. 37)			
Thymol : camphor	2.33 : 1	−33.0 (ref. 37)	0.9698 (ref. 37)	18.8 (ref. 37)			
Thymol : carvone	1 : 1		0.96920 (ref. 179)	8.76 (ref. 179)	31.3 at 20 °C (ref. 179)	0.10 (ref. 179)^b^	Refractive index: 1.51178 (ref. 179)
							Thermal conductivity/W m^−1^ K^−1^: 0.134 (ref. 179)
Thymol : carvone	2 : 1		0.90 (ref. 101)	11.24 (ref. 179)	43.57 (ref. 101)		
Thymol : cineole	1 : 1		0.95585 (ref. 179)	17.7 (ref. 179)	29.6 at 20 °C (ref. 179)	0.11 (ref. 179)^b^	Refractive index: 1.49322 (ref. 179)
							Polarity/kcal mol^−1^: 47.55 (ref. 179)
							Thermal conductivity/W m^−1^ K^−1^: 0.119 (ref. 179)
Thymol : linalool	2 : 1		0.84 (ref. 101)	7.07 (ref. 101)	37.89 (ref. 101)		
Thymol : geraniol	1 : 1		0.85 (ref. 101)	10.23 (ref. 101)	39.58 (ref. 101)		
Thymol : carvacrol	1 : 1	<−18 °C (ref. 212)					

a Density and viscosity of the water saturated DES.

b Water content before contact with water.

The terpenoids investigated in thymol-based DESs comprise a relatively narrow range of structures, dominated by oxygenated monoterpenoids. The most extensively studied system is thymol : menthol, for which a wide range of molar ratios (1 : 4.88–4.88 : 1) has been reported, followed by thymol : borneol (1 : 1–4 : 1), thymol : camphor (1 : 1.5–2.33 : 1), and thymol : carvone (1 : 1 and 2 : 1). Fenchol, terpineol, β-citronellol, linalool, geraniol, carvacrol, and cineole have generally been investigated at only one composition. Although these compounds represent hydroxyl, ketone, and ether functionalities, monoterpenoid hydrocarbons and higher terpenoids, including sesquiterpenoids and diterpenoids, are essentially absent. Thus, the particularly extensive literature on menthol contrasts with the limited data available for most other terpenoids and highlights the need for broader structural coverage.

Comparison of identical systems across independent studies generally shows better reproducibility for density than for viscosity. For thymol : menthol (1 : 1), most reported densities cluster between 0.933 and 0.938 g cm^−3^,^26,51,106,138,159,164,166,182,201^ although the value of 0.87 g cm^−3^ in ref. 101 is a clear outlier. Reported viscosities are considerably more dispersed, ranging from 28.37 to 162.12 mPa s.^51,101,106,138,159,182,201^ At 1 : 2, densities are similarly consistent (0.920–0.92384 g cm^−3^),^51,138,182^ whereas the value of 1.098 g cm^−3^ in ref. 192 appears anomalous. For thymol : menthol (2 : 1), excellent agreement is observed for both density (0.945–0.94559 g cm^−3^) and viscosity (26.10–27.50 mPa s),^51,182^ although the 9.13 mPa s value reported in ref. 124 stands as a clear outlier. For thymol : camphor (1 : 1), density varies more substantially (0.9668–0.9873 g cm^−3^),^26,37,159,160,166^ while viscosity values from ref. 159 and 160 are closely matched (20.80 and 21.10 mPa s). Melting-point data are also generally consistent for thymol : camphor (1 : 1), with values of −44.0 and −44.15 °C,^37,166^ although the much lower value of −77.66 °C reported in ref. 66 warrants caution. For thymol : menthol (1 : 1), melting points of −58.65 and −54.9 °C have been reported.^166,203^ Overall, these comparisons indicate that density is the most reproducible property, whereas viscosity is substantially more sensitive to experimental conditions. Differences in temperature, exact composition, water content, sample history, and measurement methodology should therefore be considered when evaluating apparent discrepancies.

The available data further demonstrate that molar ratio, temperature, water content, and terpenoid structure can strongly affect the thermophysical properties of thymol-based DESs. The effect of molar ratio is particularly evident for thymol : borneol and thymol : menthol at 25 °C. Increasing the thymol fraction in thymol : borneol from 1 : 1 to 4 : 1 increases density from 0.80 to 0.84 g cm^−3^ while reducing viscosity from 70.50 to 22.41 mPa s; water content before water contact simultaneously decreases from 0.018 to 0.007 wt%.^158^ Similarly, increasing the thymol fraction in thymol : menthol from 1 : 4.88 to 4.88 : 1 increases density from 0.908 to 0.958 g cm^−3^ and decreases viscosity from 52.54 to 20.16 mPa s, while surface tension increases from 28.69 to 31.14 mN m^−1^.^51^ Thus, composition has a particularly pronounced effect on viscosity, reflecting changes in intermolecular interactions and molecular mobility. The identity and structure of the terpenoid are equally important. At a 1 : 1 molar ratio, reported densities for thymol : borneol, thymol : β-citronellol, thymol : menthol, thymol : camphor, thymol : carvone, thymol : cineole, and thymol : geraniol are approximately 0.80,^158^ 0.911,^181^ 0.933,^51^ 0.98,^26^ 0.97,^179^ 0.96,^179^ and 0.85 (ref. 101) g cm^−3^, respectively. Their viscosities also differ substantially, demonstrating that replacing the terpenoid can markedly alter transport properties even at identical nominal composition. These differences cannot be explained solely by molecular mass; hydroxyl-group accessibility, molecular rigidity, cyclic *versus* acyclic structure, and hydrogen-bond-donating ability are likely to influence thymol–terpenoid interactions and molecular packing. Particularly low viscosities have been reported for thymol : carvone (1 : 1), thymol : cineole (1 : 1), thymol : linalool (2 : 1), and thymol : geraniol (1 : 1), at 8.76 ,^179^ 17.7,^179^ 7.07,^101^ and 10.23 (ref. 101) mPa s, respectively. Overall, the evidence suggests that increasing the thymol fraction generally increases density and decreases viscosity, while terpenoid structure determines the magnitude of these effects. Nevertheless, cross-study comparisons must be interpreted cautiously because water content, temperature, sample preparation, and measurement methodology may contribute as strongly as nominal composition to the observed properties.

### 6.22 Water stability

Most thymol–terpenoid DESs exhibit hydrophobic behavior and remain stable in the presence of water, forming biphasic systems. For instance, thymol : borneol,^158,159,161,162^ thymol : fenchol,^164^ thymol : terpineol,^161,162,164^ thymol : menthol (across a wide range of compositions)^26,43,45,51,55,65,66,69,71,74,85,100,103,108,112,113,117,118,120,124,131,138,147,152,154,159,161,162,164,171,182,184–186,188,193,194,198,200,201,203,205,208,209^ thymol : sobrerol,^159^ thymol : carvacrol,^212^ and thymol : camphor^26,37,47,53,65,66,80,108,113,154,159,162,164,165,168,170–172,174,176^ are generally reported as water-stable systems. This stability is often confirmed by measuring water content before and after saturation or by observing phase separation behavior. In contrast, for some systems such as thymol : β-citronellol, thymol : cineole, thymol : linalool, and thymol : geraniol, water stability has not been investigated. Nevertheless, their hydrophobic nature suggests potential immiscibility with water. An important point is that water stability may depend on composition; for example, certain molar ratios may exhibit improved phase separation or resistance to component leaching.

### 6.23 Preparation methods

The preparation of thymol–terpenoid DESs is generally straightforward and follows typical DES synthesis protocols. The most common method is heating and stirring the components until a homogeneous transparent liquid is obtained. This method has been successfully applied to nearly all systems listed in Table 2.

Alternative preparation approaches include:

• Heating without stirring (*e.g.*, thymol : borneol, thymol : camphor, and thymol : menthol in specific cases).^163^

• Stirring at room temperature (*e.g.*, thymol : menthol,^199^ thymol : carvone,^176,177^ and thymol : camphor,^80,176,177^ in some studies).

• Combined heating, stirring, and sonication to accelerate dissolution and homogenization (*e.g.*, thymol : menthol).^208^

• *In situ* formation, particularly in extraction processes where the DES forms directly in the sample matrix (*e.g.*, thymol : menthol and thymol : camphor).^26^

These variations highlight the flexibility of DES preparation and the relatively low energy requirements compared to conventional solvent synthesis.

Overall, thymol-based DESs with terpenoids represent a versatile and highly tunable class of hydrophobic solvents. Systems such as thymol : menthol and thymol : camphor are among the most comprehensively studied, with extensive thermodynamic and physicochemical data available. However, for many other terpenoids, studies remain limited to basic characterization, and full phase behavior analysis is lacking. A key challenge in this field is the inconsistency in reported eutectic compositions and temperatures, which complicates classification into deep or shallow eutectic systems. This highlights the necessity for standardized experimental methodologies and systematic thermodynamic modeling. Despite these challenges, the combination of favorable physicochemical properties, water immiscibility, and simple preparation methods makes thymol–terpenoid DESs promising candidates for green extraction, separation technologies, and other industrial applications.

### 6.24 Thymol-based DESs with quaternary ammonium salts

Thymol-based DESs formed with quaternary ammonium salts represent an important subclass of hydrophobic and tunable eutectic systems. In these mixtures, thymol typically acts as the HBD, while the quaternary ammonium salts function as HBAs. The diversity of ammonium salts, ranging from small alkyl-substituted salts to bulky, long-chain cations, enables fine control over the physicochemical properties, hydrophobicity, and water stability of the resulting DESs.

### 6.25 Molar ratios and eutectic behavior

A wide range of molar ratios has been investigated for thymol-based DESs with quaternary ammonium salts. In most systems, thymol-rich compositions (generally ≥ 2 : 1 thymol : HBA) are required to form stable liquid phases at ambient conditions. For instance, thymol : tetrabutylammonium bromide (TBABr) forms a DES at a 3 : 1 molar ratio ^67^, while thymol : tetrabutylammonium chloride (TBACl) has been reported at 2 : 1, 3 : 1, and 4 : 1 molar ratios.^70,71,214^ Similarly, systems with smaller cations such as tetramethylammonium chloride (TMACl) ^216^ and tetraethylammonium chloride (TEACl) ^215^ have been explored over a broader composition range (1 : 1 to 5 : 1). For several systems, the entire composition range has been examined to identify eutectic points through SLE phase diagrams. Notably, thymol : trimethylglycine (betaine) exhibits a well-defined eutectic point at a 3 : 1 molar ratio (*x*_th*y*_^E^ = 0.75, *T*^E^ = −15 °C) ^226^, while thymol : *N*,*N*-dimethyl-*N*,*N*-didodecylammonium chloride (DDDACl) shows a eutectic composition at 2 : 1 (*x*_th*y*_^E^ = 0.67, *T*^E^ = −20 °C) ^226^. Another remarkable system is thymol : Aliquat 336, with a eutectic point at approximately 1 : 1.22 (*x*_th*y*_^E^ ≈ 0.45) and a significantly depressed eutectic temperature (∼−80 °C) ^231^, indicating strong non-ideal interactions. However, for many other systems, SLE data are lacking, limiting a rigorous classification of their “deep” eutectic nature.

### 6.26 Physicochemical properties

Table 7 summarizes the physicochemical properties reported for thymol : quaternary ammonium salt DESs.

**Table 7:** Physicochemical properties of Thymol : Quaternary ammonium salt DESs at 25 °C

DES g cm^−3^	Molar ratio	Density	Viscosity mPa s	Other properties
Thymol : tetrabutylammonium chloride (TBACl)	2 : 1	0.9616 (ref.	>1700 (ref. 70)	Maximum miscibility of water in DES/w/w%: 0.95 (ref. 70)
Thymol : tetrabutylammonium chloride (TBACl)	4 : 1	0.9552 (ref. 70)	391.0 (ref. 70)	Maximum miscibility of water in DES/w/w%: 1.08 (ref. 70)
Thymol : N,N-dimethyl-*N*,*N*-didodecylammonium chloride (DDDACl)	2 : 1	0.88 (ref. 226)		Melting point/°C: −20 (ref. 226)
Thymol : ChCl	4 : 1	0.9812 (ref. 220)		Maximum miscibility of water in DES/w/w%: 2.25,^220^ 0.29 (ref. 226)^a^
Thymol : trimethylglycine (betaine)	3 : 1	0.96 (ref. 226)		Melting point/°C: −15 (ref. 226)
Thymol : benzyltriethylammonium chloride (BTEACl)	4 : 1	0.91,^222^ 0.9956 (ref. 121)	230 (ref. 222)	Melting point/°C: −23 (ref. 121)
Thymol : methyltrioctylammonium chloride (MTOACl)	4 : 1	0.9102 (ref. 121)		Melting point/°C: −5 (ref. 121)
Thymol : Aliquat 336	1 : 1.22	0.903 (ref. 231)	953 (ref. 231)	Refractive index: 1.4812 (ref. 231)

a Water content before contact with water.

The physicochemical characterization of this type of DESs remains limited, with available measurements mainly covering density, viscosity, melting point, maximum miscibility of water in DES and, for thymol : Aliquat 336, refractive index. Thus, compared with thymol : carboxylic acid DESs, substantial data gaps remain for interfacial and transport properties such as surface tension, thermal conductivity, electrical conductivity, and dielectric properties, which have been reported only sporadically. The quaternary ammonium components investigated so far include tetrabutylammonium chloride (TBACl), choline chloride (ChCl), *N*,*N*-dimethyl-*N*,*N*-didodecylammonium chloride (DDDACl), benzyltriethylammonium chloride (BTEACl), methyltrioctylammonium chloride (MTOACl), trimethylglycine (betaine), and Aliquat 336. Notably, the literature is strongly biased toward chloride-based ammonium salts, while other counterions (*e.g.*, bromide, iodide, or other anions) and a broader range of quaternary ammonium structures remain essentially unexplored. For thymol : BTEACl (4 : 1), for example, density is reported as 0.91 g cm^−3^^222^ and 0.9956 g cm^−3^^121^, a difference of nearly 9%, while a viscosity of 230 mPa s and a melting point of −23 °C were reported in ref. 121 and 222, respectively. For thymol : TBACl, increasing the thymol molar ratio from 2 : 1 to 4 : 1 decreases density slightly from 0.9616 to 0.9552 g cm^−3^ but produces a pronounced decrease in viscosity from >1700 to 391 mPa s ^70^, indicating that composition can strongly affect flow behavior. However, systematic molar-ratio studies are scarce, with TBACl being the clearest example; consequently, the effects of composition cannot yet be generalized across different salts.

Structural effects of the quaternary ammonium salt are also difficult to isolate because molar ratios and measurement sources vary simultaneously. Nevertheless, the reported densities span approximately 0.88–0.996 g cm^−3^ for DDDACl, MTOACl, Aliquat 336, BTEACl, ChCl, and TBACl systems, while melting points range from −23 to −5 °C for BTEACl, DDDACl, betaine, and MTOACl. The relatively low density of the long-chain DDDACl system (0.88 g cm^−3^), MTOACl system (0.9102 g cm^−3^) and Aliquat 336 (0.903 g cm^−3^) compared with BTEACl and TBACl may suggest an influence of increasing hydrophobic alkyl-chain content, but the different molar ratios and lack of consistent datasets prevent a definitive structure–property relationship. Overall, the principal limitation is not only the small number of quaternary ammonium salts investigated but also the absence of systematic studies in which salt structure, thymol ratio, and water content are varied independently under identical experimental conditions.

### 6.27 Water stability and hydrophobicity

Most thymol-based DESs with quaternary ammonium salts exhibit hydrophobic behavior and are stable in aqueous environments, forming biphasic systems. For example, thymol : TBABr ^67^, thymol : TMACl ^216^, thymol : TEACl ^215^, thymol : betaine ^226^, thymol : BTEACl,^121,221–223,228^ thymol : MTOACl,^67,121,213^ thymol : TMGHCl ^213^, thymol : DDDACl ^226^, thymol : THABr ^43^ and thymol : Aliquat 336 ^230,231^ are reported to be water-stable. However, water stability strongly depends on both the nature of the salt and the molar ratio. In the thymol : TBACl system, the 2 : 1 molar ratio is unstable due to salt leaching into the aqueous phase, whereas 3 : 1 and 4 : 1 ratios exhibit good stability ^70^. According to ref. 220, the water stability of thymol : ChCl eutectic mixtures increases with increasing the mole ratio of thymol to ChCl, so that thymol : ChCl mixture in 1 : 1 and 2 : 1 molar ratios is water-soluble, in 3 : 1 molar ratio slightly forms a separate layer with water, and in 4 : 1 molar ratio could easily form a separate layer with water. The ref. 102 points out that thymol : ChCl mixture in 2 : 1 molar ratio precipitate a solid white at the bottom of the tube, showing no stability when added to the water sample. However, in the other ref. 221, 222 and 225, a thymol : ChCl mixture with a molar ratio of 2 : 1 was used in a liquid–liquid extraction process. Namely, according to ref. 221, 222 and 225, the 2 : 1 molar ratio of thymol : ChCl mixture has shown water-immiscibility and separation from the aqueous phase.

Overall, increasing the thymol-to-salt ratio enhances hydrophobicity and water immiscibility, which is advantageous for extraction applications. Nonetheless, conflicting observations have been observed for thymol : ChCl (2 : 1) system.

### 6.28 Preparation methods

The preparation of thymol-based DESs with quaternary ammonium salts is generally straightforward and follows conventional DES synthesis protocols. The most commonly employed method is heating and stirring the components until a homogeneous, transparent liquid is formed. This approach has been used for nearly all reported systems. In some cases, alternative or complementary techniques such as sonication combined with heating have been applied to facilitate dissolution and accelerate DES formation, as reported for certain thymol : ChCl mixtures ^224^. Regardless of the method, careful control of temperature and mixing conditions is essential to ensure complete interaction between components and to avoid phase separation or incomplete dissolution.

Although thymol-based DESs with quaternary ammonium salts show significant promise due to their tunability and hydrophobic character, several limitations remain. The lack of comprehensive SLE phase diagrams for many systems restricts the ability to confirm their classification as true DESs. Additionally, physicochemical data are often limited to selected compositions, hindering the development of predictive models. Future research should focus on systematic thermodynamic characterization, including full phase diagrams and non-ideality analysis, as well as standardized evaluation of water stability. Such efforts are essential to fully exploit the potential of these DESs in separation processes, green extraction, and other industrial applications.

### 6.29 Thymol-based DESs with phenols and phenolic derivatives

Thymol-based DESs formed with phenols and phenolic derivatives constitute an important subclass of predominantly hydrophobic eutectic systems. These mixtures are typically characterized by strong intermolecular hydrogen bonding between the hydroxyl group of thymol and the functional groups of the co-component (*e.g.*, –OH, –OCH_3_, carbonyl groups), leading to significant melting point depression and the formation of liquid phases at or near ambient conditions. The available literature reveals a broad diversity of such systems in terms of composition, physicochemical properties, and stability behavior. In the case of thymol : coumarin, thymol : 6-methylcoumarin, thymol : flavone, thymol : flavanone and thymol : benzophenone systems, thymol acts as a HBD. However, in the case of thymol : salol DES, thymol acts as a HBA and for other investigated systems, both components may exhibit a dual HBD/HBA nature.

### 6.30 Molar ratios and phase behavior

A wide range of molar ratios has been explored for thymol-based DESs with phenolic derivatives. Simple phenolic systems such as thymol : phenol and thymol : 2-chlorophenol have been investigated at ratios of 1 : 1, 1 : 2, and 2 : 1.^65,232^ Also, thymol : 2,6-dimethoxyphenol (syringol) follows a similar compositional range (1 : 1, 1 : 2, and 2 : 1 molar ratios).^232,233^ In contrast, more structurally complex compounds such as coumarin ^91,103,108,113,127,138,164,168,170–172,174,196,211,235,236^ and its derivatives (6-methylcoumarin) ^236,247,248^ exhibit a much broader compositional window, including ratios from 1 : 2 to as high as 5 : 1 (thymol:coumarin) depending on the study. For three systems (thymol : salol ^154^, thymol : flavone ^241^, and thymol : flavanone ^241^), the full SLE phase diagram has been determined, providing valuable insight into eutectic composition and temperature. Notably, thymol : phenyl salicylate (salol) exhibits a well-defined eutectic point at approximately 1 : 1.5 (*x*_th*y*_^E^ = 0.4, *T*^E^ = 28 °C) ^154^, while thymol : flavone and thymol : flavanone show eutectic compositions slightly above 1.5 : 1, with eutectic temperatures of 13 °C and −5 °C, respectively ^241^. These systems demonstrate that the nature of the phenolic derivative strongly influences the extent of melting point depression and deviation from ideality. In contrast, for many systems such as thymol : phenol, thymol : 2-chlorophenol, thymol : vanillin, thymol : eugenol, and others, SLE data are not available, limiting a rigorous classification of these mixtures as deep, normal or shallow eutectic systems. Although thymol : vanillin (1 : 1) has been reported as a DES in several ref. 72, 111, 242–244 and 246, the ref. 232 and 245 have not considered thymol : vanillin (1 : 1) as a stable eutectic mixture. According to ref. 232, a 1 : 1 molar ratio of thymol : vanillin forms a liquid upon heating and retains this liquid form after being kept at room temperature for 24 hours. However, this mixture (thymol : vanillin, 1 : 1) is not stable, as it crystallizes within one week, despite appearing liquid for the first several days.^232^ Also, the ref. 245 has mentioned that 1 : 1 molar ratio of thymol : vanillin is metastable and exhibits quick crystallization at temperatures below 25 °C. However, according to the ref. 244, thymol : vanillin mixtures at the molar ratios of 1 : 2 and 2 : 1 solidify after cooling at room temperature, while the solvent with the molar ratio of 1 : 1 remains as a transparent yellow liquid at ambient conditions for at least one week.

### 6.31 Physicochemical properties

Table 8 summarizes the physicochemical properties reported for thymol : phenol and phenolic-derivative DESs at different molar ratios, with emphasis on measurements at 25 °C or near room temperature.

**Table 8:** Physicochemical properties of thymol : phenol and phenolic derivatives DESs at 25 °C (unless otherwise specified)

DES	Molar ratio	Density/g cm^−3^	Viscosity/mPa s	Other properties
Thymol : phenol	1 : 2	1.024 (ref. 232)	18.21 (ref. 232)	Melting point/°C: −9.4 (ref. 232)
Thymol : phenol	1 : 1	1.008 (ref. 232)	19.18 (ref. 232)	
Thymol : phenol	2 : 1	0.987 (ref. 232)	20.05 (ref. 232)	Melting point/°C: 6.4 (ref. 232)
Thymol : 2,6-dimethoxyphenol (syringol)	1 : 2	1.113 (ref. 232)	35.44 (ref. 232)	
Thymol : 2,6-dimethoxyphenol (syringol)	1 : 1	1.077 (ref. 232)	34.91 (ref. 232)	
Thymol : 2,6-dimethoxyphenol (syringol)	2 : 1	1.041 (ref. 232)	30.60 (ref. 232)	
Thymol : 2,6-xylenol	1 : 1	0.9922 at 20 °C (ref. 197)		Maximum miscibility of water in DES/w/w%: 0.011 (ref. 197)^b^
Thymol : coumarin	1.00 : 1.01	1.0874 (ref. 103)	10.01 at 40 °C (ref. 103)	
Thymol : coumarin	1 : 1	1.09180,^138^ 1.09024 (ref. 138)^a^	29.16,^138^ 25.82 (ref. 138)^a^	Maximum miscibility of water in DES/w/w%: 2.54 at 22 °C,^138^ 0.02173 at 22 °C (ref. 138)^b^
				Electrical conductivity/µS cm^−1^: 0.352 at 40 °C (ref. 211)
Thymol : coumarin	1.2 : 1	1.089 (ref. 164)	25.00 (ref. 164)	
Thymol : coumarin	1.5 : 1	1.07 (ref. 91)	17.92 (ref. 91)	Polarity/kcal mol^−1^: 52.17 (ref. 91)
Thymol : coumarin	2 : 1	1.05047,^138^ 1.0500 (ref. 138)^a^	31.35,^138^ 26.78 (ref. 138)^a^	Maximum miscibility of water in DES/w/w%:2.48 at 22,^138^ 0.0233 at 22 °C (ref. 138)^b^
				Electrical conductivity/µS cm^−1^: 0.244 at 40 °C (ref. 211)
Thymol : vanillin	1.5 : 1	1.066 (ref. 245)	84.10 (ref. 245)	
Thymol : vanillin	2.33 : 1	1.040 (ref. 245)	52.70 (ref. 245)	
Thymol : vanillin	4 : 1	1.017 (ref. 245)	32.18 (ref. 245)	Melting point/°C: 26 (ref. 245)
				Maximum miscibility of water in DES/w/w%:< 0.5 at 30 °C (ref. 245)
Thymol : vanillin	9 : 1	0.993 (ref. 245)	21.05 (ref. 245)	
Thymol : phenyl salicylate (salol)	1 : 1	1.10 (ref. 26)		Polarity/kcal mol^−1^: 50.13 (ref. 26)

a Density and viscosity of the water saturated DES.

b Water content before contact with water.

The physicochemical characterization of thymol : phenol and phenolic-derivative DESs remains relatively limited and highly uneven. To date, the reported properties include density, viscosity, melting point, polarity, electrical conductivity, and water miscibility/content, with density and viscosity being the most extensively investigated; in contrast, important properties such as surface tension, refractive index, speed of sound, heat capacity, static permittivity, and thermal conductivity have not been reported for this class of DESs. The investigated phenolic partners are also restricted to a small number of structurally diverse compounds, namely phenol, 2,6-dimethoxyphenol (syringol), 2,6-xylenol, coumarin, vanillin, and phenyl salicylate (salol), leaving major gaps for other phenols, alkylphenols, polyphenols, substituted phenols, and phenolic compounds with different functional groups. It is not possible to compare identical systems across different studies, as measurements taken under identical conditions are not available for any of the systems listed in Table 8. However, the molar ratios of thymol to coumarin reported in sources ^138^ (1 : 1) and 103 (1.00 : 1.01) are essentially identical, and the corresponding density values are 1.09180 g cm^−3^ (ref. 138) and 1.0874 g cm^−3^,^103^ respectively, indicating a difference of approximately 0.4%.

The effect of molar ratio is relatively systematic for thymol : phenol and thymol : syringol : increasing the thymol fraction from 1 : 2 to 2 : 1 decreases density from 1.024 to 0.987 g cm^−3^ while increasing viscosity from 18.21 to 20.05 mPa s for thymol : phenol, and decreases density from 1.113 to 1.041 g cm^−3^ and viscosity from 35.44 to 30.60 mPa s for thymol : syringol ^232^, indicating that the response of viscosity to composition is system-dependent. A similar decrease in both density and viscosity with increasing thymol fraction is observed for thymol : vanillin, from 1.066 and 84.10 mPa s at 1.5 : 1 to 0.993 and 21.05 mPa s at 9 : 1, respectively.^245^ Structural effects are evident when comparing phenolic partners: syringol-containing DESs are substantially denser and more viscous than the corresponding thymol : phenol systems,^232^ while vanillin produces particularly high viscosity, plausibly reflecting stronger and more extensive intermolecular interactions associated with its additional functional groups.^245^ Water also measurably affects the properties of thymol : coumarin DESs. Upon contact with water, the viscosity decreases from 29.16 to 25.82 mPa s for the 1 : 1 DES and from 31.35 to 26.78 mPa s for the 2 : 1 DES, whereas the corresponding densities change only slightly, from 1.09180 to 1.09024 and from 1.05047 to 1.0500 g cm^−3^, respectively.^138^ The water contents of the 1 : 1 and 2 : 1 DESs before contact with water are 0.02173 and 0.0233 at 22 °C, respectively, and increase substantially to 2.54 and 2.48 wt% after contact with water.^138^ Overall, the available data suggest that composition, phenolic structure, and water content can strongly influence viscosity, whereas density is comparatively less sensitive to hydration, but the small number of systems and the inconsistent measurement temperatures and hydration states currently prevent robust generalization across the broader family of thymol : phenolic DESs.

### 6.32 Water stability

Most thymol-based DESs with phenolic derivatives exhibit hydrophobic behavior and are reported to be stable in aqueous environments. Systems such as thymol : phenol,^232^ thymol : 2-chlorophenol,^65^ thymol : coumarin,^103,108,113,138,164,168,170–172,174,235,236^ thymol : 6-methylcoumarin,^236,247,248^ thymol : vanillin,^72,242–246^ thymol : syringol,^232,233^ thymol : eugenol,^234^ thymol : salol,^26,154^ and thymol : benzophenone^249^ are generally described as water-stable and capable of forming a separate phase. For several systems (*e.g.*, thymol : 2,6-xylenol, thymol : flavone, and thymol : flavanone), water stability data are not available, indicating the need for systematic evaluation.

### 6.33 Preparation methods

The preparation of thymol-based DESs with phenolic derivatives is generally straightforward and relies predominantly on conventional heating and stirring. This method ensures the formation of a homogeneous liquid phase by promoting hydrogen bond interactions between components. It has been successfully applied to most systems, including thymol : phenol, thymol : 2-chlorophenol, thymol : 2,6-xylenol, thymol : coumarin, thymol : vanillin, thymol : syringol, thymol : eugenol, thymol : salol, and thymol : flavone/flavanone. Alternative preparation approaches have also been reported. For example, thymol : coumarin^236^ and thymol : 6-methylcoumarin^236,247,248^ DESs have been prepared using heating combined with vortexing to enhance mixing efficiency, while thymol:benzophenone^249^ has been synthesized *via* ultrasonication without external heating, demonstrating the feasibility of energy-efficient preparation routes. Additionally, thymol:salol has been produced both by conventional heating and stirring^154^ and *via in situ* formation,^26^ where the DES is generated directly within the application system.

Overall, thymol-based DESs with phenols and phenolic derivatives exhibit considerable compositional flexibility, tunable physicochemical properties, and generally good water immiscibility, making them promising candidates for green extraction and separation technologies. However, several limitations remain. The lack of comprehensive SLE phase diagrams for many systems hinders proper classification and understanding of their thermodynamic nature. Similarly, incomplete physicochemical datasets and limited long-term stability studies restrict the rational design of these solvents for targeted applications. Future research should focus on systematic phase behavior analysis, standardized physicochemical characterization, and detailed investigation of water stability and aging effects to fully exploit the potential of this important class of thymol-based DESs.

### 6.34 Thymol-based DESs with other materials

In addition to conventional HBD–HBA systems, thymol has been combined with a wide range of “other” materials such as water, TOPO, triisobutylphosphine sulfide, bis(2-ethylhexyl) phosphoric acid, 3-hydroxyhexanoate, butyl lactate, methyl salicylate, poly (methacrylic acid-*co*-ethylene glycol dimethacrylate), 4-methoxybenzaldehyde, 2,4,5-trimethoxybenzaldehyde and 4-methylbenzaldehyde to form binary eutectic mixtures with diverse structural features and physicochemical behaviors. In these systems, thymol can act as a HBD (with water, TOPO, triisobutylphosphine sulfide, bis(2-ethylhexyl) phosphoric acid, 4-methoxybenzaldehyde, 2,4,5-trimethoxybenzaldehyde and 4-methylbenzaldehyde), HBA (with poly (methacrylic acid-*co*-ethylene glycol dimethacrylate)) or dual HBD/HBA (with 3-hydroxyhexanoate, butyl lactate, and methyl salicylate). These systems expand the applicability of thymol-based DESs, particularly in hydrophobic extraction processes, functional materials, and *in situ* applications. The available data on these systems reveal important insights into their molar composition, SLE behavior, physicochemical properties, water stability, and preparation strategies. Table 9 summarizes the physicochemical properties reported for thymol-based DESs containing water, phosphine/phosphorus-containing compounds, and 4-methoxybenzaldehyde.

**Table 9:** Physicochemical properties of thymol : other materials DESs at 25 °C (unless otherwise specified)

DES	Molar ratio	Density/g cm^−3^	Viscosity/mPa s	Other properties
Thymol : water	4.8 : 1	0.9400 (ref. 128)		Melting point/°C: −6 (ref. 128)
				Maximum miscibility of water in DES/w/w%: 2.45 128
Thymol : trioctylphosphine oxide (TOPO)	1 : 1	0.898 (ref. 127)	69.93 (ref. 127)	Refractive index: 1.4795 (ref. 250)
				Maximum miscibility of water in DES/w/w%: 15.1 (ref. 127)
Thymol : triisobutylphosphine sulfide	1 : 1	0.9386 (ref. 251)	19.1 (ref. 251)	Refractive index: 1.509 (ref. 251)
				Maximum miscibility of water in DES/w/w%: 0.779,^251^ 0.030 (ref. 251)^a^
Thymol : bis (2-ethylhexyl) phosphoric acid	2 : 1	0.90 (ref. 252)	270 (ref. 252)	
Thymol : 4-methoxybenzaldehyde		1.043 at 20 °C (ref. 246)	15.1 at 20 °C (ref. 246)	Melting point/°C: below 70 °C (ref. 246)

a Water content before contact with water.

A representative example is the thymol : water system, which has been extensively characterized over the entire composition range. The eutectic point was identified at a molar ratio of 4.8 : 1 (*x*_th*y*_^E^ = 0.83) with a eutectic temperature of −6 °C,^128^ while additional studies have examined discrete compositions such as 5 : 1 and 10 : 1.^129^ The availability of a complete SLE phase diagram confirms the eutectic nature of this system. Physicochemical properties including density, molar volume, and water content have been reported at 25 °C for the eutectic composition.^128^ Notably, this system exhibits good stability in water across the investigated compositions.^128,129^ It can be prepared either by conventional heating and stirring or *via in situ* formation, highlighting its versatility.^128,129^

Among organophosphorus-based systems, thymol has been combined with TOPO and triisobutylphosphine sulfide. In the thymol : TOPO system, the eutectic composition was identified at approximately 2.33 : 1 (*x*_th*y*_^E^ ≈ 0.7, *T*^E^ ≈ −13.15 °C) based on a full SLE phase diagram,^127^ although 1 : 1 mixtures are commonly studied.^108,127,250^ This system has been thoroughly characterized, with reported properties including refractive index,^250^ density, viscosity, and water content before and after saturation with water.^127^ It exhibits good water stability and is typically prepared *via* heating and stirring.^108,127,250^ Similarly, the thymol : triisobutylphosphine sulfide system shows a eutectic region around 1 : 1–1 : 1.5 (0.4 ≤ *x*_th*y*_^E^ ≤ 0.5), supported by SLE data.^251^ Experimental measurements of density, viscosity, and refractive index over a temperature range of 15–65 °C have been reported for the 1 : 1 composition, and the system is stable in water and prepared by heating and stirring.^251^

Thymol has also been combined with acidic extractants such as bis(2-ethylhexyl) phosphoric acid (D2EHPA). Although no SLE phase diagram has been reported for this system, several molar ratios (1 : 1, 2 : 1, and 3 : 1) have been investigated.^252^ Physicochemical properties such as density, viscosity, and melting point have been measured for the 2 : 1 composition at 25 °C.^252^ The system demonstrates good water stability and is prepared *via* heating and stirring.^252^

A distinct category includes systems formed with functional organic molecules such as 3-hydroxyhexanoate, butyl lactate, and methyl salicylate. These mixtures are typically prepared at a 1 : 1 molar ratio *via in situ* formation, without reported SLE phase diagrams or physicochemical characterization.^253^ Despite the lack of detailed thermophysical data, these systems have been reported to be stable in water, suggesting potential for practical applications where simple preparation and stability are required.

Polymeric and hybrid systems further extend the scope of thymol-based DESs. For example, thymol has been combined with poly (methacrylic acid-*co*-ethylene glycol dimethacrylate) at a 1 : 1 molar ratio to form a stable system, although no SLE or physicochemical data are available.^132^ This water-stable mixture is prepared *via* heating and stirring and represents an emerging class of eutectic-based functional materials.

Aromatic aldehyde-based systems, such as thymol : 4-methoxybenzaldehyde, thymol : 2,4,5-trimethoxybenzaldehyde, and thymol : 4-methylbenzaldehyde, have also been investigated.^246^ In the thymol : 4-methoxybenzaldehyde system, a wide range of molar ratios (from 5 : 1 to 1 : 5) has been explored,^246^ although no SLE phase diagram has been reported. Physicochemical properties including density and viscosity (for the 1 : 1 ratio at 20 °C) and melting points (for several compositions) have been measured.^246^ All these systems are reported to be stable in water and are prepared *via* heating and stirring.^246^ In contrast, for thymol : 2,4,5-trimethoxybenzaldehyde and thymol : 4-methylbenzaldehyde, only the 1 : 1 composition has been reported, without SLE or physicochemical data, although both systems exhibit water stability and are prepared similarly.^246^

Overall, thymol-based DESs with “other materials” display considerable diversity in terms of composition and properties. While some systems, such as thymol : water and thymol : TOPO, are well-characterized with complete SLE phase diagrams and detailed physicochemical data, many others remain insufficiently explored. A common feature across most systems is their preparation *via* simple heating and stirring, although *in situ* formation is also frequently employed, particularly for labile or application-specific mixtures. Water stability is generally favorable, especially for hydrophobic systems, although detailed quantitative studies are often lacking. These findings highlight both the versatility of thymol in forming eutectic mixtures with unconventional partners and the need for more systematic investigations into their phase behavior and thermophysical properties.

## 7. Applications of binary thymol-based DESs

In general, thymol-based DESs have been investigated for a wide range of emerging applications. These solvents have primarily been employed in extraction processes, a field that accounts for the largest number of published papers. Beyond extraction, thymol-based DESs have been explored in a range of emerging applications, such as CO_2_ capture, antibacterial and antifungal agents, fabrication of hydrophobic eutectogels, active extractants in continuous two-phase biomass processing, enhancement of dermal drug delivery, green solvents and structure-directing intermediates for sustainable nanomaterial synthesis, and multifunctional bioactive plasticizers in high-performance chitosan-based food packaging films. In Fig. 6, different applications of thymol-based DESs have been summarized.

**Fig. 6:**
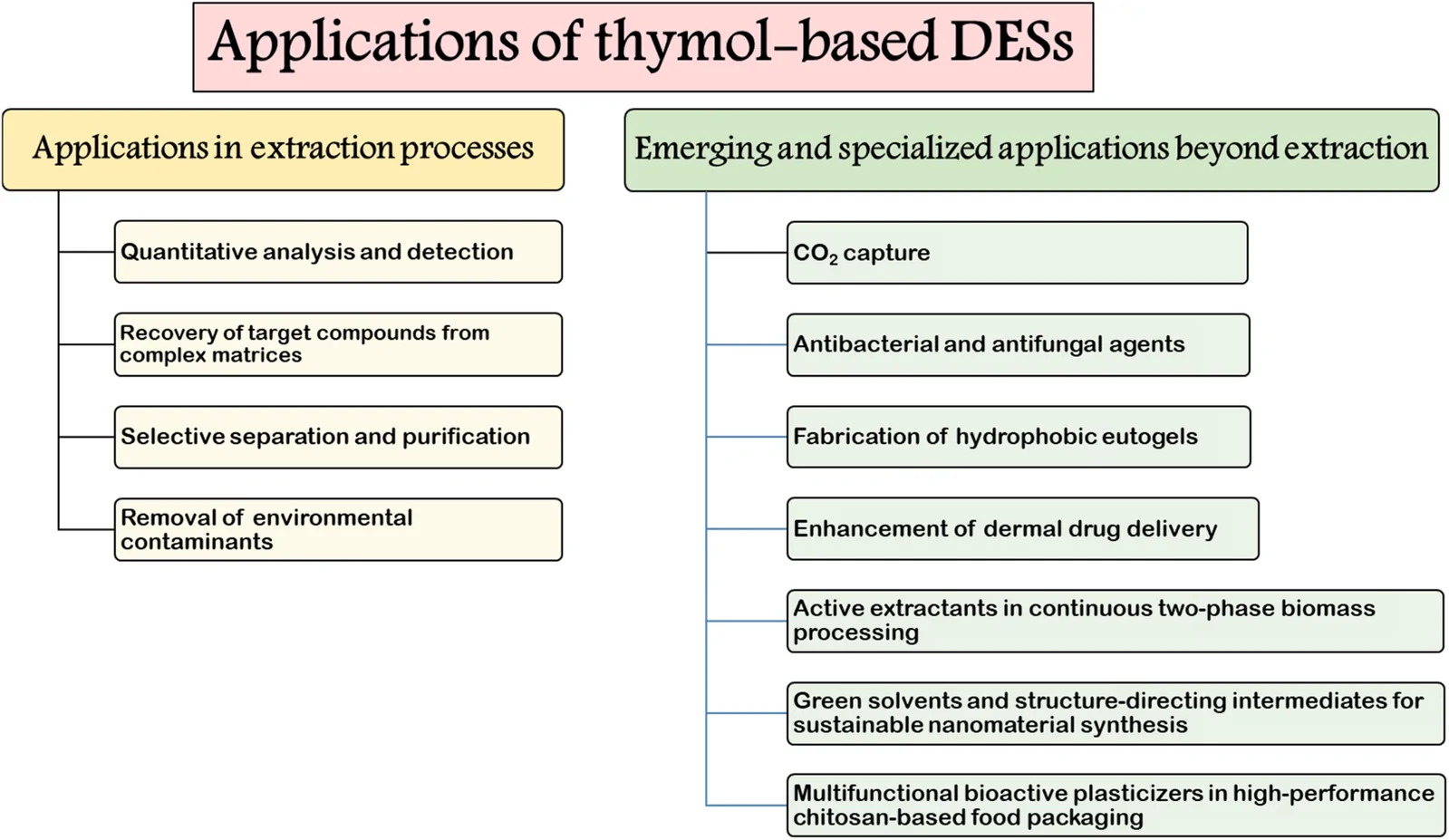
Different applications of thymol-based DESs.

### 7.1 Extraction processes

The primary applications of thymol-based DESs are in extraction processes, including liquid–liquid, vapor–liquid, solid–liquid, solid-phase, and membrane-based extractions, serving four main purposes: (1) quantitative analysis and detection, (2) recovery of target compounds from complex matrices, (3) selective separation and purification, and (4) removal of environmental contaminants. The remarkable efficacy of thymol-based DESs in extraction process can be largely attributed to their highly tunable physicochemical properties, including adjustable polarity, viscosity, density, mass transfer behavior, phase separation with water, hydrophobicity, strong hydrogen-bonding capacity and intricate intermolecular interaction networks. In addition, thymol-based DESs exhibit a wide range of molecular interactions, such as π–π stacking, electrostatic forces, van der Waals interactions, CH–π interactions, and hydrophobic effects, which collectively enhance the selective solubilization, transport, and stabilization of target analytes. These multifaceted properties also ensure excellent compatibility with various extraction and sorption techniques, including liquid–liquid extraction, solid-phase extraction, and microextraction approaches, thereby facilitating efficient, sustainable, and highly customizable strategies for different extraction applications.

A critical comparison of the reported extraction studies indicates that no single thymol-based DES composition provides universally superior performance; rather, the optimum composition is strongly dependent on the chemical nature of the target analyte and the extraction matrix. Nevertheless, several compositional trends can be identified. Thymol : fatty acid DESs, particularly thymol : decanoic acid and thymol : dodecanoic acid, have shown high extraction efficiencies for hydrophobic compounds such as bisphenol A, PAHs, PCBs, and organochlorine pesticides, where hydrophobic interactions, van der Waals forces, and π–π interactions between the aromatic thymol moiety and analytes promote favorable partitioning.^37,59,76,77^ For PAHs, thymol : camphor (1 : 1) outperformed thymol : decanoic acid and thymol : 10-undecylenic acid, illustrating that a more hydrophobic composition is not necessarily optimal and that the balance between hydrophobicity and specific interactions is crucial.^37^ Likewise, borneol : decanoic acid (1 : 3) provided superior extraction of warfarin, mainly because its composition generated an appropriate hydrophobic microenvironment while maintaining relatively low viscosity and favorable mass-transfer characteristics.^158^ Thymol : EG (2 : 1) was optimal for tricyclic antidepressants,^148^ whereas thymol : dodecanoic acid (1 : 1) maximized BPA enrichment ^59^, further demonstrating the importance of the HBA/HBD ratio. For relatively polar or ionizable analytes, thymol : ammonium salt systems, such as thymol : BTEACl and thymol : TBACl, can be advantageous because electrostatic interactions and ion pairing supplement hydrophobic partitioning.^214,265^ Overall, the highest extraction efficiencies are generally associated with DES compositions that provide an appropriate balance among polarity, hydrophobicity, viscosity, and analyte-specific interactions rather than maximizing any single property. Polarity primarily governs partitioning of polar analytes, whereas hydrophobicity favors nonpolar compounds; low-to-moderate viscosity facilitates diffusion and interfacial mass transfer; sufficiently low water solubility promotes phase separation and minimizes solvent loss; and an appropriate density difference from water facilitates phase disengagement. In addition, the availability of aromatic rings, hydrogen-bond donors/acceptors, ionic groups, and other functional moieties determines the strength and selectivity of π–π, hydrogen-bonding, electrostatic, van der Waals, and hydrophobic interactions. Importantly, these properties are strongly coupled: increasing the hydrophobic or aromatic character of a DES may improve analyte affinity but can simultaneously increase viscosity and decrease mass-transfer rates. Thus, extraction performance should be considered as a multidimensional structure–property–performance relationship, in which DES composition and molar ratio must be optimized to achieve the appropriate balance between analyte affinity, phase behavior, and mass-transfer kinetics rather than selected solely on the basis of extraction efficiency reported for a particular analyte.

#### 7.1.1. Analytical chemistry: quantitative analysis and detection

Among the various application domains of thymol-based DESs, analytical chemistry, specifically quantitative determination and detection, has garnered the most significant research interest. To date, over 100 peer-reviewed studies have documented their integration into analytical sample preparation and preconcentration workflows. This intense scholarly activity underscores the pivotal role of extraction and preconcentration in trace analysis, where analytical accuracy, sensitivity, and precision are largely dictated by the efficiency and selectivity of the sample preparation step. The superior performance of thymol-based DESs in extraction process attribute render them highly compatible with different microextraction and miniaturized techniques (such as dispersive,^55,266^ vortex-assisted,^222,267^ ultrasonic-assisted,^37,100,235,268^ air-assisted,^269^ pH-assisted,^50,59^ effervescence tablet-assisted^60^ and microwave-assisted^270^ liquid–liquid extraction techniques), which demand minimal extractant volumes, rapid equilibration, and high enrichment factors. A broad spectrum of analytes, including antibiotics,^47,49,55,104,105,225,240^ pharmaceuticals and miscellaneous drugs,^78,148,177,209,213,216,246^ polycyclic aromatic hydrocarbons (PAHs),^65,76,157,162^ pesticides,^71,119,133,151,198^ metals and metalloids,^58,98,136,217–219,265^ organic contaminants and dyes,^66,80,129,149,165,176,223,234,249^ phthalates and adipates^114,253^ and food additives and metabolites^61,134,156,162^ have been isolated by thymol-based DESs prior to instrumental analysis.

Methodologically, thymol-based DESs have been incorporated into nearly all primary extraction paradigms. Liquid–liquid extraction (LLE), particularly dispersive and microextraction variants, remains the most prevalent approach due to its simplicity, speed, and inherent compatibility with hydrophobic DES phases.^37,43,44,55,56,62,65,66,69,76,78,92,96,119,121,133,139,140,151–153,157,162,176,206,214,217,221,225,238,253,265,271^ However, these solvents have also demonstrated robust performance in solid-phase,^79,130,132,156,173,177,186,187,215,216^ membrane-based,^170–172,236,247,248^ solid–liquid,^235,238^ and vapor–liquid ^207^ extraction, extending their utility beyond conventional aqueous systems. The ability to adapt a single DES formulation across multiple extraction modes highlights the inherent versatility of thymol-centered eutectic systems.

#### 7.1.2. Bio-resource recovery: recovery of target compounds from complex matrices

Thymol-based DESs have emerged as highly versatile and sustainable media for the selective separation and recovery of high-value compounds from complex matrices. Their unique physicochemical profile enables the effective solubilization of non-polar and moderately polar targets that are often challenging to isolate using conventional aqueous or hydrophilic systems. By eliminating or minimizing solvent transfer into aqueous phases, these hydrophobic DESs provide superior selectivity and enrichment factors across a diverse array of solid and liquid matrices. The recovery of lipophilic nutraceuticals and phytochemicals represents one of the most successful applications of thymol-centered eutectic systems. In plant-based matrices, these solvents have demonstrated exceptional performance in extracting triterpenic acids, pigments, and alkaloids. For instance, a menthol : thymol (1 : 2) DES achieved a total triterpenic acid yield of 2.9 wt% from Eucalyptus globulus bark, surpassing the efficiency of traditional organic solvents.^183^ Similarly, thymol-based systems have been optimized for the recovery of ursolic acid from loquat leaves^124^ and have shown a dramatic 278% increase in triterpenic acid extraction from Carya cathayensis husks when coupled with ultrasound-assisted techniques.^101^ The stabilization and extraction of carotenoids and xanthophylls also highlight the advantages of thymol's antioxidant properties. Studies have reported high-efficiency recovery of lycopene from tomatoes,^75^ lutein from *Scenedesmus* sp.,^164^ and astaxanthin from Haematococcus pluvialis (60–75% recovery).^123^ Notably, thymol : dodecanoic acid enabled the recovery of 22.03 mg g^−1^ of fucoxanthin from microalgae, maintaining solvent stability and recyclability over seven consecutive cycles.^52^ Furthermore, thymol-based DESs have proven effective for nitrogenous compounds, such as isoquinoline alkaloids from Chelidonium majus, yielding up to 180% more coptisine than acidified methanol,^169^ and achieving 91% recovery of usnic acid from lichens.^163^

Thymol-based HDESs serve as potent extractants for biorefinery and fermentation-derived products. In the recovery of biofuels, thymol : 2,6-dimethoxyphenol systems have shown high distribution coefficients and selectivity for acetone, ethanol, and n-butanol from aqueous fermentation broths.^232^ For platform chemicals, thymol : decanoic acid achieved up to 96.9% recovery of furfural and 73.4% of 5-hydroxymethylfurfural (HMF) from hydrothermal process waters using countercurrent configurations.^83^ Additionally, thymol-based systems have successfully isolated organic acids, including volatile fatty acids (VFAs), lactic acid, and levulinic acid, from complex lignocellulosic hydrolysates and silage juices, often outperforming 1-octanol in terms of equilibrium extraction capacity and selectivity.^109,116,184^

Expanding beyond biomass, thymol-based DESs have been applied to polymer science and e-waste recycling. A thymol : vanillin DES facilitated the green extraction of polyhydroxyalkanoates (PHAs) from microbial biomass with 99% purity, offering a sustainable alternative to the hazardous chloroform benchmark.^245^ In the field of hydrometallurgy, a thymol : triisobutylphosphine sulfide (TIBPS) system enabled the selective, multistage recovery of Li(i), Cu(ii), Al(iii), and Fe(iii) from spent LFP battery leachates, with extraction efficiencies exceeding 99%.^251^

Recent advancements have integrated thymol-based DESs into membrane technologies, specifically for water treatment and pervaporation. In reverse osmosis (RO), thymol-acid DESs have been used as co-solvents during interfacial polymerization to modulate polyamide layer morphology, resulting in membranes with doubled water flux and enhanced chlorine resistance.^68,97^ In pervaporation, thymol-containing polymer-inclusion membranes (*e.g.*, using PEBAX (poly (ether-*block*-amide)) or PDMS (polydimethylsiloxane) supports) have improved butanol/water separation factors, although challenges regarding the long-term leaching of the DES from the membrane matrix remain a subject of ongoing research.^142,143^

#### 7.1.3. Separation science: selective separation and purification

Thymol-based DESs have been effectively employed for selective extraction in two main areas: metal recovery and biochemical/biomass applications. For metal recovery, thymol combined with long-chain carboxylic acids (C8–C18) selectively extracted Cu^2+^ from mildly acidic solutions, achieving high loading capacities (0.64 g L^−1^) and excellent selectivity over Co^2+^ and Ni^2+^, with separation factors above 500 and 154, and stable performance over multiple cycles.^36^ Thymol : aliquat 336 efficiently separated Co/Ni pairs from hydrochloric acid solutions *via* formation of [CoCl_4_]^2−^ complexes, optimized at an O/A phase ratio of 1 : 5.^231^ Thymol : lidocaine enabled selective extraction of Au(iii), Pd(ii), and Pt (iv) from acidic leach solutions, achieving capacities of 761.2, 98.3, and 299.3 g L^−1^, respectively, with stepwise selective separation and solvent regeneration.^141^ Thymol : 2-hexyldecanoic acid (HDA) selectively recovered thorium from radioactive waste leach solutions through a cation-exchange mechanism, attaining >98% Th extraction and loading capacities up to 3.80 × 10^3^ mg L^−1^.^126^ Thymol : TOPO DESs selectively extracted Pd^2+^ and Pt^4+^ from HCl solutions with high separation factors (*S*_PdFe_ ≈ 41, *S*_PdCu_ ≈ 140) and efficiently separated Sm^3+^ from aqueous leach solutions of spent SmCo magnets over Co^2+^*via* neutral extraction with nitrate coordination, with extraction efficiency influenced by the hydrogen bond donor structure.^127,250^

In biochemical and biomass applications, thymol : octanoic, decanoic, and dodecanoic acid DESs purified polyphenol oxidase (PPO) from fresh tea leaves, achieving 78.44% recovery and an 8.26-fold purification.^85^ Thymol : menthol DESs extracted lactic acid from microfiltered sweet sorghum silage press juice, improving recovery from 37.0% to 76.2% under controlled pH,^200^ and selectively separated organic acids and furfural from hydrolysis or fermentation aqueous mixtures, favoring more hydrophobic solutes such as levulinic acid and furfural.^185^

#### 7.1.4. Environmental remediation: removal of environmental contaminants

Thymol-based DESs have emerged as versatile green solvents for the removal and preconcentration of diverse pollutants from environmental matrices, including water, wastewater, oil-contaminated soils, and air. Based on the type of contaminants addressed, their applications can be categorized into: (i) pharmaceuticals and antibiotics, (ii) phenolic and nitrophenolic compounds, (iii) dyes, (iv) heavy metals and metalloids, (v) volatile organic compounds (VOCs), (vi) nanoplastics and microplastics, and (vii) petroleum hydrocarbons.

Specifically, thymol-based DESs, including acid-based mixtures^46,50,64^ and graphene-oxide functionalized composites,^108^ have demonstrated exceptional efficacy in removing pharmaceuticals and antibiotics from wastewater, often achieving extraction yields and recoveries exceeding 94–99% through strong intermolecular interactions. Similarly, mixtures with menthol,^45,117^ alkylammonium salts,^112^ and organic acids^117^ have shown outstanding performance in eliminating phenolic and nitrophenolic pollutants, frequently surpassing 90–99% efficiency due to highly selective hydrophobic and hydrogen-bonding interactions. Furthermore, ionic, non-ionic, and advanced nanohybrid or magnetic thymol-based composites exhibit remarkable versatility in dye remediation, reaching removal efficiencies over 98–99% for a broad spectrum of cationic and anionic dyes *via* synergistic electrostatic interactions and π–π stacking.^67,135,147,155,272,273^ Beyond organic pollutants, hydrophobic DESs such as thymol : decanoic acid effectively remediate toxic metal contamination, reducing arsenic (As^3+^) concentrations to levels well below WHO drinking water guidelines through carboxylic acid complexation and hydrophobic partitioning.^87^ Recent advancements also highlight the efficacy of thymol : 2,6-dimethoxyphenol in micro- and nanoplastic remediation, achieving over 95% extraction efficiency for PET and PS.^233^ In the field of resource recovery, thymol mixtures with lauric acid^88^ or menthol-coated magnetic sponges^120^ provide highly efficient and reusable solutions for hydrocarbon recovery and oil spill remediation. Finally, thymol : decanoic acid (1 : 1) serves as a sustainable alternative for VOC mitigation, utilizing vapor–liquid extraction to achieve high acetone absorption capacity while maintaining low water affinity.^57^

### 7.2 Gas capture: efficient CO_2_ sequestration

DESs have emerged as a promising class of green and customizable media for CO_2_ capture. They offer benefits, including low cost, simple preparation, reduced toxicity, and the possibility of using natural and renewable components.^6,274^ Since phenolate based ILs solvents have shown strong potential for CO_2_ capture,^275^ hydrophobic DESs incorporating thymol, are considered promising candidates for efficient CO_2_ absorption. In fact, thymol-based DESs exhibit remarkable structural versatility, functioning as HBDs, HBAs, or neutral phenolic molecules to enable a broad spectrum of CO_2_ absorption mechanisms ranging from physical dissolution to strong chemisorption. Terpenoid and nonionic phenolic-based hydrophobic DESs, formed with components such as menthol,^197^ carvone,^180^ cineole,^180^ camphor,^122^ and 2,6-xylenol,^197^ capture CO_2_ purely through physical dissolution; while effective at high pressures (30–40 bar), their capacity significantly diminishes at flue-gas-relevant pressures. In contrast, functional thymol : polyamine DESs, such as those incorporating [TETA]Cl or [TEPA]Cl, facilitate strong chemisorption *via* carbamate/carboxylate formation at free amine sites, with the [TEPA]Cl : thymol (1 : 3) system reaching an exceptional capacity of 1.355 mol mol^−1^ while maintaining hydrophobicity and low regeneration energy.^144^ Moderate chemisorption is achieved in thymol : amine systems (*e.g.*, with MEA or MDEA), where thymol acts as the HBA to balance thermal stability with amine reactivity, offering rapid absorption and high reversibility.^145^ Furthermore, anion-functionalized DESs derived from thymol-based phenolate anions (*e.g.*, [Et_4_N][Thy] and [DBUH][Thy]) provide tunable CO_2_ chemistry, where the choice of HBD, such as ethylene glycol or 4-methylimidazole, determines whether reaction occurs at the phenolate site alone or involves the formation of additional carbonate species.^276,277^

### 7.3 Biomedical use: antibacterial and antifungal applications

Thymol-based DESs emerge as potent antibacterial and antifungal agents, offering a sustainable and effective alternative to conventional antimicrobials. As a natural phenolic constituent of essential oils, thymol exerts intrinsic antimicrobial effects by disrupting microbial membranes, altering permeability, and inducing the leakage of critical intracellular components. When formulated as a DES, these transition into synergistic systems where the co-forming HBDs or HBAs enhance thymol's solubility, stability, and bioavailability. The inherent properties of these DESs, including low volatility, tunable viscosity, and adjustable acidity, allow for the precise tailoring of antimicrobial activity against specific bacterial or fungal pathogens. Furthermore, their natural origin and biodegradability align with green chemistry principles, minimizing environmental impact and toxicological concerns. Notably, thymol-based DESs have demonstrated robust activity against both Gram-positive and Gram-negative bacteria, as well as pathogenic fungi, including antibiotic-resistant strains, underscoring their vast potential across medical, food, and environmental sectors. Despite these compelling advantages, the application of thymol-based DESs in this field remains emerging and relatively underexplored, with key findings from the limited available literature.^131,202,203^ Syed *et al.*^202^ utilized thymol : menthol (8 : 2) nanoemulsions to achieve potent bactericidal activity against both Gram-positive and Gram-negative bacteria, particularly C. acnes, by leveraging the synergistic membrane-disrupting effects of terpene components and the increased surface area of 81 nm droplets. Similarly, Pale-Ezquivel *et al.*^131^ showed that a hydrophobic thymol : pyruvic acid (1 : 1) DES effectively inhibits the germination of coffee leaf rust (Hemileia vastatrix) by inducing cytoplasmic disorganization, with chitosan-coated nanoemulsions providing a controlled-release barrier on leaf surfaces. Furthermore, Zhang *et al.*^203^ developed a sustained-release system by immobilizing thymol : menthol (1 : 1) emulsion droplets within a cross-linked poly (HEA) gel, which extended the antimicrobial cycle up to 34 days for food preservation. Across these applications, the primary mechanism involves the disruption of microbial cell membranes, enzyme inhibition, and intracellular content leakage, with the DES framework stabilizing active components and reducing volatility compared to pure thymol.

### 7.4 Material science: fabrication of hydrophobic eutectogels

Recently, DESs have been extensively employed to formulate eutectogels, a novel class of gel electrolytes that provide environmentally friendly, flexible, and conductive matrices suitable for smart sensing, wearable electronics, and energy storage devices.^278–287^ Within this framework, thymol-based eutectogels emerge as a highly advantageous platform for advanced material design, effectively merging the intrinsic bioactivity and hydrophobicity of thymol with the tunable physicochemical properties of DESs. A primary strength of these eutectogels is their exceptional stability in underwater or high-humidity environments, which overcomes the performance degradation and water-sensitivity typically associated with conventional hydrogels and hydrophilic DES-based systems. Their dynamically cross-linked polymer architectures impart critical functionalities such as high stretchability, autonomous self-healing, and strong self-adhesion, while maintaining robust mechanical integrity, thermal stability, and ionic conductivity. Furthermore, their sustainable profile, characterized by recyclability and cost-effectiveness, positions them as eco-friendly alternatives for next-generation flexible electronics and wet adhesives. Despite this unique combination of underwater durability and multifunctionality, the development of thymol-based eutectogels remains an emerging field, with two foundational studies reported to date.^229,237^

Li *et al.*^229^ developed a multifunctional hydrophobic eutectogel (HEG) by performing in situ* photopolymerization of soft (EGPEA) and hard (IBA) acrylate monomers within a benzyltriethylammonium chloride (TEBAC) : thymol (1 : 2) DES. In this system, the thymol-based DES serves as both a conductive medium and a structural reinforcer, strengthening the polymer network through a suite of non-covalent interactions, including hydrogen bonding, π–π, and cation–π interactions. These supramolecular forces impart the material with exceptional characteristics, such as robust underwater adhesion to diverse substrates (*e.g.*, metals, ceramics, and PTFE), rapid autonomous self-healing in both air and water, and high mechanical toughness. Furthermore, the HEG maintains stable ionic conductivity for flexible strain and pressure sensing in high-humidity or aquatic environments and is fully recyclable through dissolution and re-polymerization, aligning with green chemistry principles. In a similar vein, Liang *et al.*^237^ reported a hydrophobic eutectogel utilizing a thymol : coumarin (2 : 1) DES integrated into a poly (ionic liquid)/polyacrylate framework. Classified as an “Eutectogels-S″ system, the non-polymerizable DES functions as a multifunctional solvent, plasticizer, and dynamic physical cross-linker, enabling rapid one-step *in situ* photopolymerization. The resulting material exhibits remarkable water resistance with negligible leakage, high optical transparency (∼92%), and outstanding mechanical toughness (∼512 kJ m^−3^). Key functional features include autonomous self-healing with up to 96.8% efficiency and stable ionic conductivity for reliable sensing of human motion and touch in both air and underwater environments. Moreover, the HEG is fully recyclable *via* solvent dissolution and recasting, providing a sustainable solution for flexible electronics and advanced underwater sensors.

### 7.5 Other emerging applications

In addition to the applications discussed in the preceding sections, thymol-based DESs have been explored, albeit to a more limited extent, in several emerging areas. These include their use as active extractants in continuous biphasic biomass processing,^212^ as functional enhancers in transdermal drug delivery systems,^204^ as green solvents and structure-directing media for the sustainable synthesis of functional nanomaterials,^190^ and as multifunctional bioactive plasticizers for high-performance chitosan food packaging films.^118^ These studies highlight the remarkable versatility of thymol-based DESs and their potential to expand into novel applications beyond conventional extraction and analytical contexts.

Kopp *et al.*^212^ utilized a thymol : carvacrol (1 : 1) DES as a multifunctional extractant and reaction stabilizer for the continuous production of 5-hydroxymethylfurfural (HMF) in biphasic microreactors. In this intensified process, the DES effectively partitions HMF from the aqueous phase *via* strong π–π stacking and hydrogen bonding, achieving extraction efficiencies of up to 96% and superior HMF yields (52 mol%) compared to conventional solvents such as methyl isobutyl ketone (MIBK). Crucially, the DES functions as a chemical suppressor of degradation pathways by extracting HMF before it undergoes side reactions to form humins, thereby significantly improving the carbon balance. With its low viscosity, minimal toxicity, and bio-based nature, this system serves as a sustainable and high-performance platform for the intensified synthesis of furan-based chemicals.^212^

In the field of pharmaceutical sciences, Czyrski *et al.*^204^ investigated a menthol : thymol DES as a potent permeation enhancer for the transdermal delivery of hydrophobic drugs like clotrimazole. In this context, the DES acts as a specialized lipid-modulating and plasticizing agent, specifically targeting the intercellular lipid domains of the stratum corneum (SC) rather than the protein-rich corneocytes. Advanced spectroscopic analyses (ATR-FTIR and solid-state ^13^C MAS NMR) revealed that the terpene-based DES disrupts van der Waals interactions within lipid lamellae, significantly increasing the molecular mobility of lipid acyl chains and cholesterol segments. While the DES does not directly alter keratin structure, it functions synergistically with propylene glycol (PG); by loosening the lipid barrier, the DES facilitates the deeper penetration of PG-solubilized drugs. This highlights the DES as a versatile, tunable tool for optimizing skin permeability through specialized intercellular pathways.

Furthermore, Sherlin *et al.*^190^ developed a high-performance electrochemical sensor for the real-time detection of hydroxychloroquine (HCQ) by leveraging a thymol : menthol-based DES as a multifunctional medium. The DES serves as a green, structure-directing solvent that enables the sustainable synthesis of crystalline alkali niobate (ANbO_3_, A = Na, K) nanoparticles with uniform dispersion and minimal aggregation. By facilitating the decoration of functionalized carbon nanofibers (f-CNF) with these nanoparticles, the DES-mediated process creates a hybrid nanocomposite with an increased surface area and efficient electron transport pathways. The resulting electrodes exhibit exceptional sensitivity and low detection limits, driven by reduced charge transfer resistance and strong π–π and hydrogen-bonding interactions with the analyte, demonstrating the power of thymol-based DESs in advancing sustainable nanotechnology for environmental monitoring.

Finally, thymol-based HDESs (specifically thymol : octanoic acid and thymol : menthol) have been successfully utilized as multifunctional green plasticizers and bioactive agents to enhance the properties of chitosan (CS) films**.**^118^ At low loadings (0.5–1.5 wt%), these DESs integrate into the chitosan matrix through an extensive network of non-covalent interactions, with thymol acting as the primary hydrogen bond donor. This molecular integration modulates the polymer's crystallinity and chain mobility, resulting in CS–DES composite films with significantly improved tensile strength, superior hydrophobicity, and enhanced UV-blocking capabilities compared to traditional glycerol-plasticized films. Moreover, the inherent bioactivity of the DES components imparts robust antibacterial and antioxidant properties to the matrix, providing a sustainable and high-performance strategy for active food packaging applications.

## 8. Thymol-based ternary DESs (TDESs)

Thymol-based ternary DESs (TDESs) represent an advanced evolution of conventional binary DESs, distinguished by the incorporation of a third component, typically an organic acid, alcohol, polyol, or amino acids, into a binary thymol-based mixture. This additional component fundamentally alters the hydrogen-bonding network, molecular packing, and thermodynamic profile of the solvent, enabling precise modulation of key physicochemical properties such as viscosity, density, melting point, pH, and conductivity. Compared to binary DESs, TDESs generally exhibit lower viscosity and melting points, enhanced fluidity, and improved solute solvation, which facilitate mass transfer, electrochemical performance, and process compatibility. The third component can also act as a structural modulator, disrupting crystalline packing and increasing free volume, thereby stabilizing the liquid phase at lower temperatures and widening the operational window for industrial applications. The tunability of TDESs extends to pH adjustment, ionic conductivity, and thermal stability, allowing the design of task-specific solvents for different applications. Experimental studies have demonstrated that the identity and molar ratio of the third component critically influence these properties; for example, polyols such as ethylene glycol reduce viscosity and enhance conductivity, whereas acids modulate pH and solubilization capacity. The development of thymol-based TDESs is primarily motivated by the limitations and trade-offs inherent to binary DESs. Although binary thymol-based DESs offer considerable compositional tunability, modification of the ratio or identity of only two components may not provide sufficient flexibility to simultaneously achieve the desired melting point, viscosity, polarity, solubility, conductivity, and chemical stability. Introducing a third component provides an additional degree of freedom for independently or synergistically tuning these properties and, in some cases, overcoming unfavorable characteristics of the parent binary DES.^42^ For example, a third component can weaken strong intermolecular interactions responsible for high viscosity, disrupt molecular packing and depress the melting point, or introduce additional functional groups that enhance the solubility or selective interaction with target compounds. Importantly, the objective is therefore not simply to replace binary DESs with ternary formulations, but to achieve a more favorable balance among competing properties for a specific application 42. This expanded compositional space can enable the simultaneous optimization of transport properties, phase behavior, solvation capacity, acidity/basicity, and application-specific functionality, which may be difficult to attain using binary systems alone. Nevertheless, the addition of a third component does not inherently guarantee superior performance; the benefits depend strongly on the chemical nature and concentration of the added component and must be evaluated against potential drawbacks such as increased formulation complexity, cost, and environmental impact. Thus, TDESs should be viewed as a rational strategy for overcoming specific limitations of binary thymol-based DESs and for developing more precisely tailored solvents rather than as universally superior alternatives.^288^

### 8.1 Types and composition of thymol-based TDESs

Compared with thymol-based binary DESs, which have been extensively investigated and prepared using a wide variety of components, studies on thymol-based TDESs remain scarce. Consequently, only a limited number of thymol-based TDESs have been reported to date, as summarized in Table 10.

**Table 10:** Thymol-based TDESs

TDESs	Ref.	TDESs	Ref.	TDESs	Ref.
TBABr : thymol : octanoic acid; (1 : 1 : 1), (1 : 2 : 1), (1 : 3 : 1), (1 : 1 : 2), (1 : 1 : 3), (1 : 1 : 4), (1 : 0 : 2), and (1 : 0 : 4)	289	ChCl : thymol : octanol (1 : 1 : 2), ChCl : thymol : benzyl alcohol (1 : 1 : 4), ChCl : thymol : octanoic acid (1 : 1 : 1), and ChCl : thymol : nonanoic acid (1 : 2 : 2)	42	Thymol : ethylene glycol : benzyl alcohol (2 : 2 : 1), thymol : ethylene glycol : octanol (1 : 1 : 1), thymol : decanoic acid : benzyl alcohol (1 : 1 : 1), and thymol : decanoic acid : octanol (1 : 1 : 1)	290
Thymol : PEG : acetic acid (1 : 2 : 2), thymol:PEG:acrylic acid (1 : 2:2), thymol:PEG:oxalic acid (1 : 2:1), and thymol:EG:acetic acid (1 : 2:2)	291	l-leucine : thymol : lactic acid (1 : 3 : 4)	292	Thymol : decanoic acid : di (2-ethylhexyl)phosphate	95
		Amino acid : thymol : lactic acid, 1 : 2 : 5		Binary thymol : 1-decanoic acid DES (1 : 1 w/w) and introducing 2% (w/w) DEHP	
		Amino acids: alanine, asparagine, glycine, histidine, isoleucine, l-leucine, l-lysine, methionine, phenylalanine, threonine, tryptophan, tyrosine, and valine			
ChCl : thymol : ethylene glycol (1 : 1 : 4)	293	Menthol : thymol : dodecanoic acid (1 : 1 : 1, 1 : 3 : 1, 3 : 5 : 1, 5 : 3 : 1, 5 : 5 : 3, 3 : 5 : 5)	294	Lauric acid : thymol : camphor (1 : 3 : 1)	295
ChCl : thymol : oleic acid (1 : 1 : 1, 1 : 1:2 and 1 : 2 : 2)	219	Heptanoic acid : thymol : formic acid (1 : 2 : 48)	296	Thymol : menthol : octanoic acid (1 : 1 : 1)	118

As can be seen from Table 10, reported thymol-based TDESs include systems based on quaternary ammonium salts (TBAB, ChCl),^42,219,289,293^ fatty acids (octanoic, nonanoic, decanoic, dodecanoic, lauric, oleic, heptanoic acids),^42,95,118,219,289,292,294–296^ alcohols and polyols (octanol, benzyl alcohol, ethylene glycol)^42,290,293^, polymeric compounds (PEG),^291^ amino acids (alanine, asparagine, glycine, histidine, isoleucine, l-leucine, l-lysine, methionine, phenylalanine, threonine, tryptophan, tyrosine, and valine)^292^, multifunctional organic acids (lactic, acetic, formic, oxalic, acrylic acids),^291,292,295,296^ terpenes (menthol and camphor)^118,294,295^ and auxiliary functional components such as di (2-ethylhexyl) phosphate.^95^

Across these systems, thymol plays a central role due to its phenolic structure and aromaticity. It mainly functions as a hydrophobic HBD, although in some cases it may also behave as a weak HBA, enabling strong hydrogen bonding, π–π interactions, and hydrophobic affinity toward aromatic and moderately polar analytes.^42,118,289–291,293–295^ Quaternary ammonium salts such as TBAB and ChCl typically act as strong HBAs, providing ionic character and stabilizing the eutectic structure.^42,219,289,293^ Fatty acids serve as HBDs that significantly reduce melting points and viscosity while increasing hydrophobicity and phase separation efficiency.^42,118,219,289,292,294–296^ Alcohols and polyols contribute to polarity adjustment, hydrogen-bond network extension, and improved mass transfer,^42,290,293^ whereas polymeric PEG reduces effective viscosity and enhances solvent stability and extraction capacity ^291^. Amino acids, acting mainly as HBAs, introduce biocompatibility and allow fine control over polarity and fluidity in NADES systems ^292^.

In all reported studies, the transition from binary to ternary DES formulations resulted in markedly improved physicochemical properties. Compared with corresponding binary DESs (*e.g.*, TBAB : thymol, ChCl : thymol, thymol : fatty acid, thymol : alcohol), thymol-based TDESs consistently exhibited lower viscosity, lower melting points, enhanced hydrophobicity, improved dispersion behavior, and greater structural tunability. These enhancements arise from synergistic hydrogen bonding and cooperative interactions among the three components, which are not attainable in simpler binary systems.

### 8.2 Applications of thymol-based TDESs

Thymol-based TDESs have been applied in analytical chemistry, separation science, electrochemistry, materials science, and food and environmental analysis, where their superior physicochemical properties translate into enhanced performance compared with binary DESs. In liquid–liquid extraction techniques, including D-LLE,^289,291,295,296^ air-bubbles assisted D-LLE ^42^, vortex-assisted emulsification LLE (VAE-LLE) ^290^, effervescence-assisted dispersive LLE (EAD-LLE) ^296^, and direct-immersion single-drop extraction (DI–SDE) ^219^, thymol-based TDESs have been successfully used for the extraction of phenols ^289^, antibiotics,^42,290,296^ pesticides,^291,292,294^ metal ions,^219,295^ and peptides.^95^ In nearly all cases, ternary systems outperformed their binary counterparts by providing higher enrichment factors, lower detection limits, faster extraction kinetics, and improved precision. These improvements were directly linked to reduced viscosity, enhanced dispersion, stronger analyte–solvent interactions (π–π, hydrophobic, and hydrogen bonding), and more efficient phase separation.^42,219,289–292,295,296^

Several studies demonstrated that thymol-based TDESs enable superior extraction of organic contaminants, such as nitrophenols,^289^ tetracyclines,^42,290^ fluoroquinolones,^296^ organochlorine pesticides,^294^ and organophosphorus pesticides,^292^ compared with corresponding binary DESs. In metal analysis, thymol-based TDESs achieved higher extraction efficiencies for thallium and zinc at lower pH values and shorter extraction times than binary systems.^219,295^ In electromembrane extraction, thymol-based TDESs provided improved selectivity and stability for peptide extraction from plasma compared with alternative eutectic compositions.^95^

Beyond extraction, thymol-based TDESs have been applied as functional media and modifiers. In electrochemical sensing, a ChCl : thymol : ethylene glycol TDES served as an active electropolymerization medium, yielding thicker, more uniform, and more conductive polymer films than aqueous solutions or binary DESs, thereby enhancing sensor sensitivity and stability.^293^ As surface modifiers for magnetic MOF-based sorbents, thymol-based TDESs produced more uniform coatings, reduced agglomeration, and improved enrichment performance compared with binary DES-modified materials.^294^

In polymer and packaging applications, thymol-based binary and ternary DESs have been employed as green plasticizers and bioactive agents in chitosan films.^118^ The ternary thymol : menthol : octanoic acid DES consistently outperformed binary DESs in terms of mechanical strength, hydrophobicity, barrier properties, and antibacterial and antioxidant activity, leading to improved food preservation performance.

## 9. Conclusions, challenges and future directions

This review underscores the substantial progress achieved in the design and understanding of thymol-based DESs, highlighting their emergence as a versatile and highly tunable class of functional liquids. Thymol, owing to its dual HBD/HBA capability and natural origin, serves as a flexible building block for a wide range of binary and ternary eutectic systems. The resulting compositional diversity, spanning carboxylic acids, alcohols, amines, terpenoids, quaternary ammonium salts, and phenolic derivatives, enables precise tuning of key physicochemical properties such as polarity, viscosity, density, and hydrophobicity. This adaptability, combined with generally simple and scalable preparation methods (*e.g.*, heating–stirring, sonication, and *in situ* formation), reinforces the practical appeal of thymol-based DESs for both laboratory and potential industrial applications.

From a fundamental perspective, thymol-based DESs exhibit complex and highly tunable intermolecular interactions, including hydrogen bonding, van der Waals forces, π–π interactions, and hydrophobic effects, which collectively dictate their thermophysical behavior. Their often hydrophobic nature and adjustable water immiscibility make them particularly attractive for separation and extraction processes. However, water stability remains strongly composition-dependent, with some systems displaying contradictory behavior, emphasizing the need for systematic and standardized investigations.

Despite these advances, several critical challenges continue to limit the rational design, reproducibility, and large-scale implementation of thymol-based DESs. One of the most pressing issues is the lack of systematic thermodynamic characterization. Complete solid–liquid equilibrium phase diagrams are available for only a limited number of systems, making it difficult to unequivocally classify mixtures as true deep eutectic systems and to distinguish them from non-ideal or shallow eutectics. Inconsistencies in reported eutectic compositions and temperatures further highlight the absence of standardized experimental protocols, undermining data reliability and cross-study comparability.

Equally important is the insufficient availability of comprehensive physicochemical datasets. While certain well-studied systems have been characterized in detail, many thymol-based DESs lack complete information on key properties such as viscosity, density, thermal stability, interfacial tension, dielectric behavior, and transport coefficients across relevant temperature and composition ranges. This data fragmentation restricts the development of predictive models and limits the ability to tailor DESs for targeted applications.

Stability-related challenges also remain significant. The behavior of thymol-based DESs in the presence of water is highly sensitive to composition and environmental conditions, with potential phase separation, structural disruption, or component leaching occurring in some systems. Moreover, long-term stability, aging effects, and the influence of impurities or repeated use cycles are rarely addressed, despite their importance for real-world applications. From a molecular standpoint, the complexity of intermolecular interactions in these systems is not yet fully understood, and the lack of unified theoretical frameworks further constrains predictive design.

Another notable limitation is the relatively narrow exploration of compositional space, particularly for ternary and multicomponent systems. Although binary DESs dominate the literature, more complex systems offer enhanced tunability and performance but remain underexplored, especially in terms of phase behavior and structure–property relationships.

In addition, while thymol-based DESs are often described as green solvents, their environmental impact and toxicity profiles are not yet comprehensively established. The overall safety of a DES depends on its full composition and behavior in real environments, and systematic ecotoxicological assessments and life-cycle analyses are still scarce. This gap poses challenges for regulatory acceptance and large-scale adoption.

Looking forward, several research priorities can be identified to advance the field. First, systematic thermodynamic characterization, including complete SLE phase diagrams, activity coefficients, and rigorous modeling using approaches such as COSMO-RS and SAFT, is essential for accurate classification and predictive design. Second, the establishment of standardized experimental protocols and the generation of high-quality, comprehensive physicochemical datasets will greatly enhance reproducibility and data usability. Third, deeper insights into water–DES interactions and stability mechanisms are needed, particularly for applications involving aqueous systems. Molecular-level investigations using advanced spectroscopic techniques, molecular dynamics simulations, and quantum chemical calculations will be crucial to elucidate structure–property relationships. Fourth, expanding the design space to include ternary and multifunctional systems, as well as bio-based and task-specific components, offers significant opportunities to enhance performance and introduce new functionalities. Integration with computational screening and machine learning tools can further accelerate discovery.

From an application and engineering perspective, greater emphasis should be placed on process integration, solvent recyclability, and scale-up. The incorporation of life cycle assessment (LCA) and techno-economic analysis (TEA) into early-stage research will be critical to validate the sustainability and economic viability of THY-DESs. Addressing challenges related to viscosity, phase behavior, and separation efficiency will also be essential for industrial deployment.

Expanding the application scope of thymol-based DESs beyond traditional extraction and separation processes into emerging areas such as advanced materials, energy systems, CO_2_ capture, and biomedical technologies represents an exciting frontier. The development of hybrid systems, including eutectogels, DES-based composites, and nano-structured materials, may further broaden their technological impact.

Despite the high potential of thymol-based DESs as sustainable solvents, their industrial application requires a more rigorous assessment of their thermodynamic properties, toxicity, and long-term stability. The bio-based origin of thymol does not necessarily guarantee environmental safety, as the performance of these solvents depends on composition, intermolecular interactions, water content, and operating conditions. Many thymol-based DESs still lack comprehensive thermodynamic characterization, including SLE phase diagrams, activity coefficients, and temperature-dependent physicochemical properties, which are essential for confirming DES formation and designing industrial processes.^30^ Their toxicity and environmental safety must also be systematically evaluated, as biological components do not necessarily guarantee low toxicity. Standard studies on cytotoxicity, biodegradability, and environmental toxicity are required.^297^ Furthermore, long-term stability under real-world conditions, including exposure to water, storage, recycling, and repeated use, has not yet been sufficiently investigated. Future studies should integrate thermodynamic modeling, toxicity assessment, stability evaluation, and life-cycle analysis to enable the reliable industrial application of thymol-based DESs.^30^

In conclusion, thymol-based DESs constitute a rapidly evolving and highly promising platform in green solvent science. However, unlocking their full potential requires coordinated efforts in thermodynamic understanding, property characterization, molecular-level insight, and process engineering. By addressing current challenges and embracing interdisciplinary approaches, thymol-based DESs are well-positioned to transition from promising laboratory systems to robust, sustainable, and application-ready technologies.

## Conflicts of interest

There are no conflicts to declare.

## Data Availability

No primary research results, software or code have been included and no new data were generated or analysed as part of this review.
